# Tailoring Nanomaterial Cross-Linkers through Lanthanide–Ligand Pairs: Guidance for Fine-Tuning the Structures and Properties of Luminescent Nanocomposite Hydrogels

**DOI:** 10.1021/acs.inorgchem.5c00130

**Published:** 2025-04-24

**Authors:** Yu-Chia Su, Li Chu Tseng, Wei-Tao Peng, Chao-Ping Hsu, Yi-Cheun Yeh

**Affiliations:** † Institute of Polymer Science and Engineering, National Taiwan University, Taipei 10617, Taiwan; ‡ Institute of Chemistry, Academia Sinica, Taipei 115201, Taiwan; § Department of Chemistry, Tunghai University, Taichung 40704, Taiwan; ∥ Physics Division, National Center for Theoretical Sciences, Taipei 106319, Taiwan

## Abstract

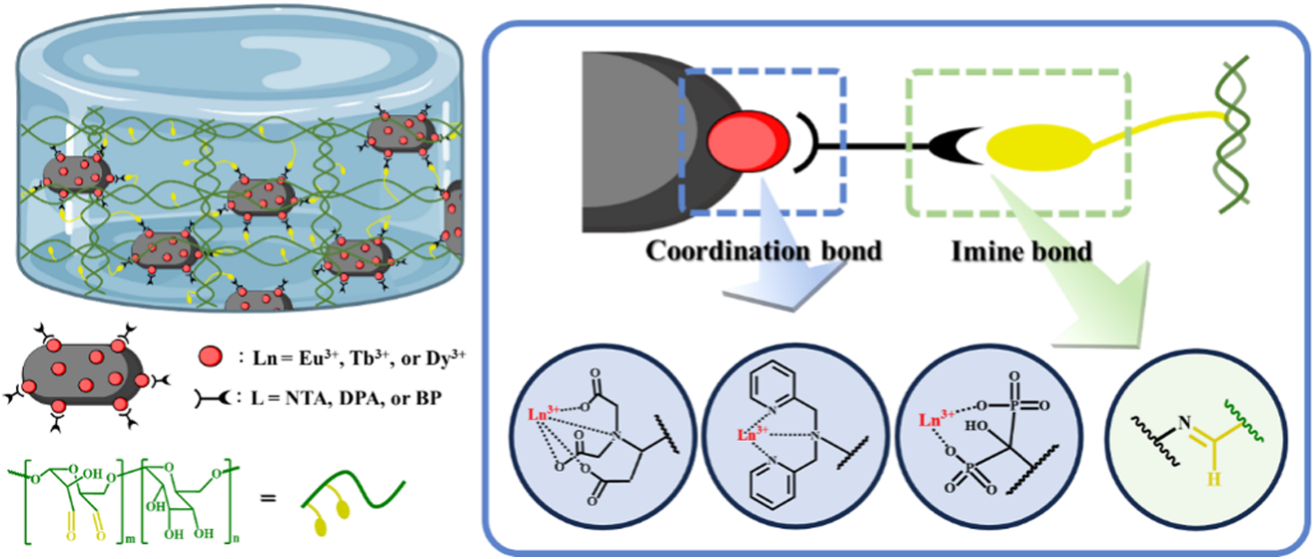

Integrating luminescent nanomaterials into hydrogels provides unique optical properties and improves their mechanical features for various applications. It is challenging but highly desirable to develop a versatile luminescent nanocomposite hydrogel system with tunable structures and properties to expand the potential uses of luminescent materials. Here, multiple amine-functionalized lanthanide-containing hydroxyapatites are synthesized as tailored nanomaterial cross-linkers to interact with polydextran aldehyde through imine bonds. The microstructure, gelation time, luminescence, rheological behavior, mechanical properties, thermal stability, degradation, and swelling capability of the luminescent lanthanide-containing nanocomposite hydrogels are systematically investigated. This study reveals that the strong binding affinity between surface metal ions and capping ligands of the nanomaterial cross-linkers contributes to the densest network and the highest mechanical properties of the nanocomposite hydrogels. In addition, these nanocomposite hydrogels possess dynamic features of self-healing, shear-thinning, and injectability, improving their suitability for advanced applications. The luminescent lanthanide-containing nanocomposite lyophilized hydrogels are also demonstrated in the differentiation of volatile organic compounds. Taken together, the adjustable microstructures and characteristics of this lanthanide-containing nanocomposite hydrogel system highlight its potential for offering guidance in producing diverse luminescent materials with definable performances across various fields.

## Abstract 2

Integrating luminescent nanomaterials into hydrogels provides unique optical properties and improves their mechanical features for various applications.

## Introduction

Lanthanide (Ln)-containing hydrogels have emerged as promising luminescent materials for applications of sensors,^1−3^ probes,^4,5^ imaging agents,^6^ photocatalysts,^7,8^ and light-emitting devices.^9^ Ln-containing hydrogels span a broad luminescence spectrum from ultraviolet to near-infrared and are characterized by a high color purity and long luminescence lifetime. Also, the lanthanide complexes into hydrogels at the molecular level prove to be easily achievable and enable the production of luminescent hydrogels characterized by uniform structures and stable performance. Recently, the increased interest in these Ln-containing hydrogels stems from their unique ability to change color in response to external stimuli through absorption (chromogenic) or emission (fluorochromic) processes.^10^ Nevertheless, the high water content of hydrogels often suppresses the luminescence of lanthanide complexes.^11^ Therefore, embedding lanthanide ions into nanomaterials to avoid interference by water molecules has become a promising strategy for preparing highly luminescent hydrogels. In addition, the nanomaterials can work as physical fillers or chemical cross-linkers in the polymeric matrix to improve the mechanical properties and stability of hydrogels.

Lanthanide ions have been incorporated into several nanomaterials (e.g., carbon nanotubes,^12^ metal–organic frameworks,^13−15^ nanoclay,^16,17^ and hydroxyapatite^18^) or forming upconversion nanoparticles (e.g., NaYF_4_:Ln^7,19,20^ and NaGdF_4_:Ln^5^) for synthesizing nanocomposite hydrogels. Cheng et al. developed a bilayered hydrogel of P(*N*-isopropylacrylamide-*co*-acrylamide) (P(NIPAm-*co*-AAm)) and poly(ethylene glycol) (PEG) containing multiwalled carbon nanotubes (MWCNTs) and NaYF_4_:Yb^3+^/Er^3+^ nanoparticles for simultaneous drug delivery and upconversion luminescence labeling.^20^ Li et al. developed luminescent nanocomposite hydrogels through *in situ* copolymerization of the acrylamide monomers and lanthanide-loaded clay nanosheets, where the nanocomposite hydrogels present outstanding stretchability, elasticity, and reversible luminescence on/off switch triggered by pH changes.^16^ Leu Alexa et al. synthesized nanocomposite hydrogels from gelatin methacryloyl (GelMA) and cerium-doped hydroxyapatite, where they also demonstrated the superior three-dimensional (3D) printability, structural integrity, and osteogenic capability of hydrogels for bone tissue regeneration.^18^ Recently, our group has reported an innovative lanthanide-containing nanocomposite hydrogel by embedding terbium-doped laponite (Tb^3+^@Lap) within the polymeric network of polyethylenimine-modified gelatin and polydextran aldehyde (PG/PDA) through dynamic covalent and noncovalent bonds.^21^ With shear-thinning and self-healing abilities, these hydrogels were suitable for electrospinning and 3D printing. Furthermore, these hydrogels act as efficient luminescent sensors for copper ions, demonstrating the potential for advanced sensing applications.

Despite the unique properties and broad applications of Ln-containing nanocomposite hydrogels,^22,23^ current Ln-containing nanocomposite hydrogels still face some challenges. For example, some Ln-containing nanocomposite hydrogels are susceptible to water-induced luminescence quenching due to the hydration of lanthanide ions.^11^ In addition, several Ln-containing nanocomposite hydrogels exhibit weak mechanical properties and poor self-recovery characteristics due to the uneven distribution of nanomaterials or the absence of chemical bonding between the nanomaterials and the polymers in the hydrogel network.^18,24^ Most importantly, it is highly desirable to develop a versatile luminescent nanocomposite hydrogel system with tunable structures and properties to reveal the structure–property relationship as well as expand the potential applications of luminescent materials.

Here, we hypothesize that utilizing tailorable lanthanide-containing nanomaterials as chemical cross-linkers for polymers will result in nanocomposite hydrogels with definable structures and properties to meet the key requirements for advanced applications. In particular, incorporating Ln-containing nanomaterials into hydrogels via dynamic bonding will greatly simplify the fabrication process through rapid and spontaneous cross-linking dynamics. While several surface-functionalized nanomaterials have been utilized as dynamic cross-linkers for the spontaneous generation of nanocomposite hydrogels,^25,26^ to the best of our knowledge, this approach has not yet been applied to Ln-containing nanocomposite hydrogel systems to enable their *in situ* formation.

**Scheme 1:**
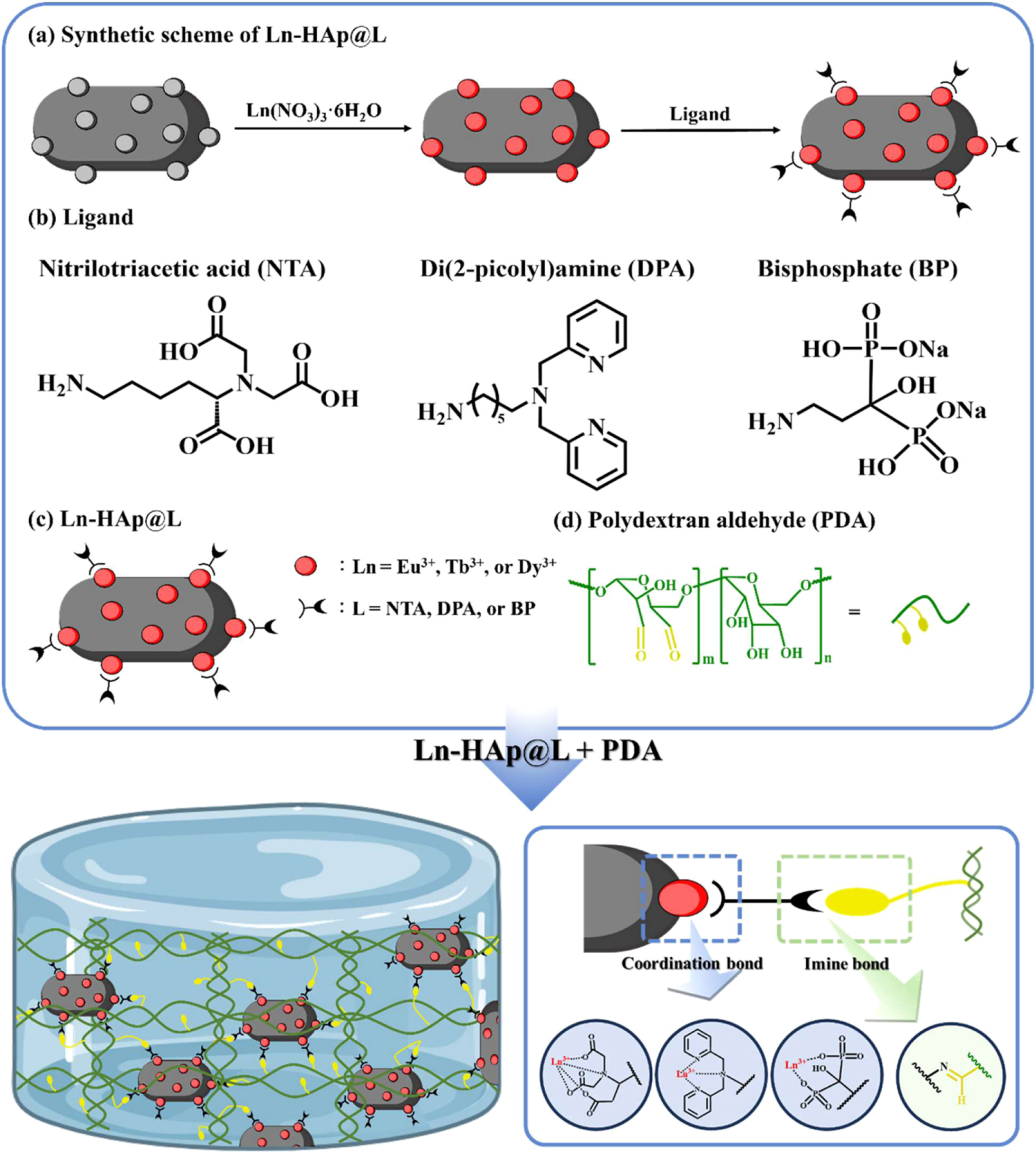
Schematic Illustration of Lanthanide-Containing Nanocomposite Hydrogel Preparation and Internal Chemistry (a) Synthetic scheme of surface-functionalized lanthanide-modified hydroxyapatite (Ln-HAp@L). Schematic illustrations of (b) chelate ligands, (c) Ln-HAp@L, and (d) PDA.

In this study, unlike other nanocomposite hydrogels made with nanomaterials that are difficult to further modify in terms of components or surface capping ligands,^27^ hydroxyapatite (HAp) is a tailorable nanomaterial platform that can be doped with lanthanides^28,29^ and covered with organic ligands,^30,31^ allowing it to be employed as an engineerable luminescent nanomaterial cross-linker for polymers. A series of Ln-containing nanocomposite hydrogels were fabricated by cross-linking polydextran aldehyde (PDA) with amine-functionalized Ln-containing HAp through dynamic imine bonds (Scheme 1). The obtained Ln-containing nanocomposite hydrogels displayed tunable microstructure and properties by fabricating HAp cross-linkers through selecting various lanthanide ions (i.e., europium **(**Eu^3+^), terbium (Tb^3+^), and dysprosium (Dy^3+^)) and capping ligands featuring amine terminals and chelating head groups (i.e., nitrilotriacetic acid (NTA), di(2-picolyl)amine (DPA), and bisphosphonate (BP)). These nanocomposite hydrogels also possessed superior dynamic networks to perform self-healing, shear-thinning, and injectability. The luminescent lanthanide-containing lyophilized nanocomposite hydrogels have also been shown to differentiate volatile organic compounds (i.e., acetic acid, ammonia, and formaldehyde) effectively by utilizing the luminescent fingerprints of the samples analyzed through linear discriminant analysis (LDA). Our studies demonstrate the critical role of the nanomaterial cross-linkers in engineering nanocomposite hydrogels, where the microstructures, mechanical strength, and dynamic features of the nanocomposite hydrogels are associated with the interactions between metal ions and capping ligands of the nanomaterial cross-linkers.

## Results and Discussion

### Characterizations of Surface-Functionalized Lanthanide-Containing Hydroxyapatites

Surface-functionalized Ln-containing hydroxyapatites were synthesized through a stepwise procedure (Scheme 1a). First, hydroxyapatites were doped with lanthanide ions (i.e., Eu^3+^, Tb^3+^, or Dy^3+^) through the precipitation method, forming Ln-HAp (i.e., Eu-HAp, Tb-HAp, or Dy-HAp).^32^ Subsequently, Ln-HAp were capped with ligands featuring amino terminus and chelating head groups [i.e., nitrilotriacetic acid (NTA), di(2-picolyl)amine (DPA), or bisphosphonate (BP)] to obtain Ln-HAp@L (i.e., Ln-HAp@NTA, Ln-HAp@DPA, or Ln-HAp@BP) (Scheme 1b). Therefore, nine types of surface-functionalized lanthanide-containing HAp were prepared in this study: Eu-HAp@NTA, Eu-HAp@DPA, Eu-HAp@BP, Tb-HAp@NTA, Tb-HAp@DPA, Tb-HAp@BP, Dy-HAp@NTA, Dy-HAp@DPA, and Dy-HAp@BP (Scheme 1c).

Scanning electron microscopy (SEM) revealed the rod-shaped morphology of pristine HAp, Ln-HAp, and Ln-HAp@L (Figure S1). Elemental mapping performed through energy-dispersive X-ray spectroscopy (EDS) was applied to further analyze the distribution of lanthanide ions within the HAp. Taking Eu-HAp@L as a representative sample, elements such as carbon (C), nitrogen (N), oxygen (O), and europium (Eu) were identified as Figure S2. Eu and N elements can be attributed to the doping of the Eu element and the associated ligand, respectively. The uniform dispersion of Eu and N elements shown in the SEM images also indicated a homogeneous distribution of lanthanide ions and ligands within the Eu-HAp@L.

**Figure 1:**
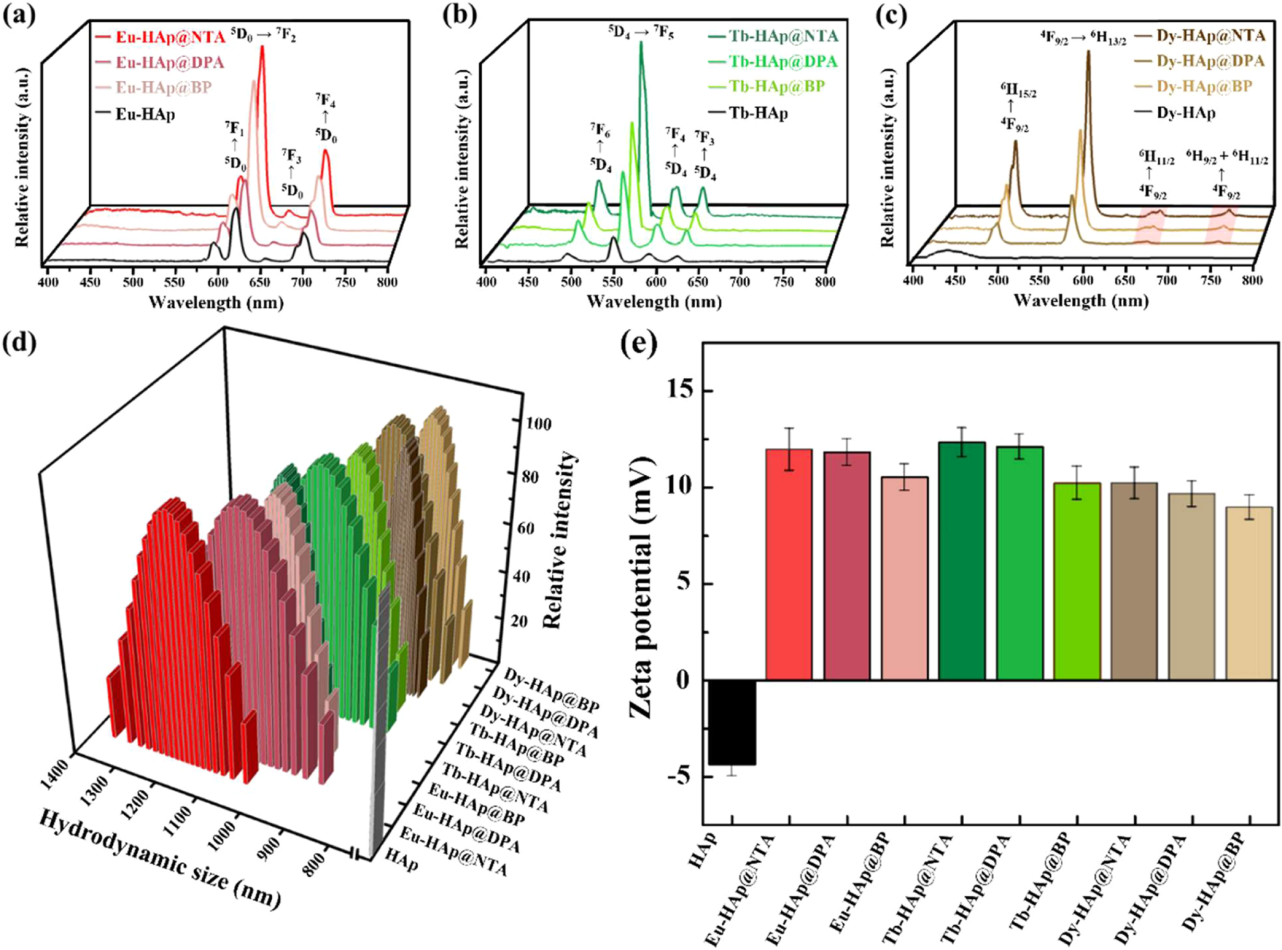
(a–c) Luminescence spectra of Ln-HAp and Ln-HAp@L. (d) Hydrodynamic sizes and (e) ζ-potentials of HAp and Ln-HAp@L.

The Ln-containing HAp displayed distinct luminescence under UV irradiation (Figure S3), showing Eu-HAp@L, Tb-HAp@L, and Dy-HAp@L with red-, green-, and yellow-green luminescence, respectively. The luminescence differences between these Ln-HAp@L can be uncovered by using a spectrometer to analyze their luminescence spectra and intensities after altering the type of lanthanide ions and capping ligands. Eu-HAp and En-HAp@L showed a luminescence peak at 616 nm, predominantly due to the ^5^D_0_ → ^7^F_2_ electronic transition, resulting in red light luminescence (Figure 1a).^33,34^ Additional peaks at 593, 652, and 702 nm corresponded to the ^5^D_0_ → ^7^F*_n_* transitions (*n* = 1, 3, and 4). Tb-HAp and Tb-HAp@L emitted primarily around 489, 543, 588, and 620 nm, correlating with the ^5^D_4_ → ^7^F_J_ transitions (*J* = 6, 5, 4, and 3), with the ^5^D_4_ → ^7^F_5_ transition being the most prominent for green luminescence (Figure 1b).^35,36^ Dy-HAp and Dy-HAp@L exhibited four main luminescence peaks, each linked to specific transitions from ^4^F_9/2_ to different ^6^H and ^6^F states, with the transition from ^4^F_9/2_ to ^6^H_13/2_ being the strongest (Figure 1c).^37^

It is well-documented that the luminescence efficiency of lanthanide complexes correlates significantly with the excitation energy levels of the coordinating ligands, known as the antenna effect.^38,39^ The introduction of ligands can effectively increase the energy transfer efficiency from the ligands to the lanthanide ions to enhance luminescence, which is attributed to the fact that ligands can absorb light energy and then transfer it to the lanthanide ions through intramolecular energy transfer or vibrational relaxation. Here, the luminescence efficiency of Ln-HAp@L was quantified by using photoluminescence quantum yield (PLQY), defined as the ratio of emitted photons to absorbed photons. An enhancement in PLQY was observed in functionalized HAp compared to pristine HAp. (Table S1). For instance, the PLQY value for Eu-HAp@NTA, Eu-HAp@DPA, and Eu-HAp@BP was measured at 1.91, 0.96, and 1.59, respectively, representing a substantial improvement compared to the unmodified Eu-HAp (0.62). The luminescence differences between the Ln-HAp@L can be further revealed using the Commission Internationale de l’Éclairage (CIE) system (Figure S4). The luminescence results indicated that the three ligands effectively enhance the energy absorption and subsequent transfer to the embedded lanthanide ions in the HAp matrix. Notably, NTA enhanced the PLQY of functionalized HAp more effectively than DPA and BP, suggesting that NTA facilitated more efficient energy transfer to the lanthanide ions.^40,41^ NTA features three carboxylic acid groups and one nitrogen atom that can coordinate with a lanthanide ion, resulting in a multidentate structure that promotes more stable and robust complex formation than DPA and BP. The chelation effect of NTA greatly enhances the stability of the lanthanide complexes while minimizing nonradiative energy losses that can occur in free solutions or poorly coordinated environments. Overall, the stable coordination geometry of the NTA-lanthanide complex is favorable for energy transfer processes, resulting in strong luminescent properties. We also examined the absorption spectra of Ln-HAp@L, revealing that the absorption intensity at 360 nm follows the order NTA > BP > DPA, irrespective of the lanthanide type (Figure S5). Given that the absorption intensity is correlated to the luminescence intensity of materials, Ln-HAp@NTA, with the highest absorption intensity at an excitation wavelength of 360 nm, showed the highest luminescence intensity compared to Ln-HAp@BP and Ln-HAp@DPA.

Electrospray ionization mass spectrometry (ESI-MS) was further used to analyze Ln-HAp@L and confirm the presence of the surface ligand on the HAp. Before ESI-MS analysis, Ln-HAp@L was immersed in a hydrochloric acid (HCl) solution to induce the cleavage of the metal–ligand bonds to facilitate the release of ligands from the metal ions on HAp. Taking Eu-HAp@L as a representative example, the ESI-MS spectra for Eu-HAp@NTA, Eu-HAp@DPA, and Eu-HAp@BP showed peaks with mass-to-charge ratios of 263.24, 299.36, and 274.63, respectively, confirming that these ligands were coordinated with the lanthanide ions on the HAp surface (Figure S6). On the other hand, inductively coupled plasma mass spectrometry (ICP-MS) was applied to quantify the amount of lanthanide ions in Ln-HAp and Ln-HAp@L, and the ninhydrin assay was utilized to determine the surface amine amount of Ln-HAp@L. The lanthanide concentrations in Ln-HAp and Ln-HAp@L ranged from 0.88 to 1.16 mmol/g, and the surface amine content of Ln-HAp@L was between 0.85 and 0.93 mmol/g (Table S2).

Dynamic light scattering (DLS) was used to provide valuable insights into the hydrodynamic size of Ln-containing HAp. The hydrodynamic size of Eu-HAp, Tb-HAp, and Dy-HAp was ∼710, 706, and 654 nm, respectively (Figure S7a). These hydrodynamic sizes were considerably larger than the pristine HAp (∼174 nm), as the hydration radius of the lanthanide ions was larger than that of the calcium(II) (Ca^2+^) ions.^42^ In addition, the hydrodynamic size of Eu-HAp@NTA, Eu-HAp@DPA, Eu-HAp@BP, Tb-HAp@NTA, Tb-HAp@DPA, Tb-HAp@BP, Dy-HAp@NTA, Dy-HAp@DPA, and Dy-HAp@BP was ∼1185, 1038, 953, 1048, 1021, 902, 855, 890, and 823 nm, respectively, showing that the incorporation of ligands into the Ln-HAp led to a significant increase in the hydrodynamic size of functionalized HAp (Figure 1d). This could be attributed to the presence of amino groups on the Ln-HAp@L surface, which enhanced the availability of binding sites for water molecules through hydrogen bonding. Zeta potential measurement was applied to reveal the surface charge of functionalized HAp. The zeta potentials of HAp, Eu-HAp, Tb-HAp, and Dy-HAp were ca. −4.33, 3.23, 3.52, and 3.55 mV, respectively (Figure S7b). These findings suggested that incorporating lanthanide ions into HAp modified the surface charge through ion substitution, where trivalent Eu^3+^ ions replaced divalent Ca^2+^ ions, introducing extra positive charges and increasing the overall positive surface charge of the HAp. Following the introduction of amine-containing ligands to the Ln-HAp surface, the surface charge of HAp increased to over 10 mV (Figure 1e), confirming the successful incorporation of amino groups on the Ln-HAp@L surface as the amine groups (–NH_2_) of the capping ligands can become protonated to generate a positively charged surface (–NH_3_^+^) on the Ln-HAp. The X-ray diffraction (XRD) patterns of HAp and Ln-HAp@L are shown in Figure S8. The XRD peaks of HAp were assigned to the hexagonal phase of HAp, with distinct intensities at 26, 33, 34, 35, and 40° (JCPDS file 09-0432). The nine patterns of the Ln-HAp@L were very similar to that of HAp, indicating that these Ln-doped HAp samples still maintain the main crystal structure of HAp.

Several characteristic chemical groups of pristine HAp and functionalized HAp were observed using Fourier transform infrared (FTIR) spectroscopy, showing phosphate (PO_4_^3–^) at 568 and 1057 cm^–1^, hydrogen phosphate (HPO_4_^2–^) at 600 cm^–1^, carbonate (CO_3_^2–^) at 1452 cm^–1^, and hydroxyl (OH^–^) at 3536 cm^–1^ (Figures S9–S11). In the comparison between the FTIR spectra of Eu-HAp and Eu-HAp@NTA, the carboxylate asymmetric vibration band shifts from 1340 to 1400 cm^–1^, indicating NTA was successfully coordinated with Eu^3+^ ions on Eu-HAp surface (Figure S9).^43^ The N–H band of the NTA also shifted from 1662 to 1634 cm^–1^ when NTA presented on the Ln-HAp surface, further confirming coordination bonding between the carboxylate groups and Eu^3+^ ions. Similarly, the FTIR spectra of Tb-HAp@NTA and Dy-HAp@NTA showed distinctive bands of carboxylate asymmetric vibration at 1417 and 1419 cm^–1^, respectively (Figure S9). For Ln-HAp@DPA, the pyridine vibration band shifted from 1521 to 1456 cm^–1^ was observed in the FTIR spectra of Eu-HAp@DPA, and the N–H band of DPA also shifted from 1682 to 1627 cm^–1^ after coordinated with Eu^3+^ ions. Similarly, for Tb-HAp@DPA and Dy-HAp@DPA, N–H bands of DPA at 1660 and 1693 cm^–1^ were observed, suggesting coordination with Tb^3+^ and Dy^3+^ ions, respectively (Figure S10). The FTIR spectra of Eu-HAp@BP showed an overlap between the phosphate groups, which complicated the observation of shifts in the phosphate group. However, a distinct shift in the N–H of BP from 1654 to 1626 cm^–1^ was clearly evident in the spectra of Eu-HAp@BP, and a similar shift was also noticed in the spectrum of Tb-HAp@BP and Dy-HAp@BP (Figure S11). Taken together, these FTIR spectral results provided valuable insights into the complex surface chemistry and structural modifications introduced into HAp, elucidating their functional characteristics.

X-ray photoelectron spectroscopy (XPS) was employed to analyze the surface of HAp and investigate the bonding between the ligands and lanthanide ions. Taking Eu-HAp@L as a representative example, the XPS spectrum of Eu-HAp@NTA revealed distinct peaks corresponding to Eu 3d, C 1s, N 1s, and O 1s (Figure S12).^44−47^ In the Eu 3d fine spectrum of Eu-HAp@NTA, two peaks were deconvoluted at 1138.05 and 1167.05 eV, attributed to the Eu^3+^ (3/2) and Eu^3+^ (5/2) species, respectively. For the C 1s spectrum of Eu-HAp@NTA, four distinctive peaks were observed at 284.80, 288.63, 289.90, and 290.46 eV, corresponding to C–C, C–N, C–O, and C = O/O=C=O, respectively. The N 1s spectrum of Eu-HAp@NTA showed three peaks at 399.45, 400.48, and 401.77 eV, corresponding to NH_2_/N–Eu, N–H, and N–C, respectively. The O 1s spectral band of Eu-HAp@NTA displayed five distinct peaks with binding energies of 530.22, 532.02, 532.08, 534.10, and 535.89 eV, which were attributed to NTA ligand fragments, such as O = C/O–Eu, O–C, O = C–O, and C–O–H, and the phosphate group (O–P) from HAp, respectively. These spectral analyses confirmed the presence of Eu doping on the surface of HAp and subsequent NTA coordination. Similarly, the Eu–N and Eu–O bands were observed in the spectra of Eu-HAp@DPA and Eu-HAp@BP, indicating that the ligands were coordinated with the Eu^3+^ ion of Eu-HAp.

### Formations, Luminescence, and Microstructures of Nanocomposite Hydrogels

Rheological analysis was utilized to systematically optimize the formation of PDA/Ln-HAp@L nanocomposite hydrogels. Both PDA and PDA/HAp presented a liquid-like state as the storage modulus (*G*′) was higher than the loss modulus (*G*″) under the continuous time sweep (Figure S13a). On the other hand, the *G*′ of the PDA/Eu-HAp sample was higher than its *G*″, indicating that a solid-like state was observed. This change is likely attributed to the interactions between the Eu^3+^ ions of Eu-HAp and PDA, as the *G*′ was quite low (∼27.3 Pa) and cannot form a stable shape in a gel state. When Eu-HAp@NTA was introduced to the polymeric matrix of dextran or PDA, both dextran/Eu-HAp@NTA and PDA/Eu-HAp@NTA presented a solid-like state based on the rheological results. Notably, PDA/Eu-HAp@NTA possessed a significantly higher *G*′ (∼1085 Pa) compared to dextran/Eu-HAp@NTA (*G*′ ∼ 37 Pa), which highlighted the contributions of the imine bond formation between the amines of Eu-HAp@NTA and the aldehydes of PDA to strengthen the PDA/Eu-HAp@NTA network. As a control experiment, Ln, an amine-terminated ligand, and PDA were mixed for hydrogel formation. However, the mixture resulted in a sol-like status with low mechanical stability, rather than producing robust hydrogels (Figure S14). Therefore, surface-functionalized Ln-HAp@L acted as a physical filler and chemical cross-linker to facilitate the hydrogel formation and also improve the mechanical properties of the hydrogels, which are crucial for constructing reliable and reproducible hydrogel-based applications.

A dose-dependent experiment was further applied to reveal the impact of the imine cross-links in the PDA/Eu-HAp@NTA network by varying the concentrations of Eu-HAp@NTA. The resulting *G*′ of PDA/Eu-HAp@NTA nanocomposite hydrogels with Eu-HAp@NTA of 2.5, 5, 7.5, and 10 wt % were ∼77, 488, 829, and 1085 Pa, respectively (Figure S13b). Therefore, the optimized condition for PDA/Eu-HAp@NTA hydrogel formation was using Eu-HAp@NTA of 10 wt % and PDA of 7 wt % to obtain a stable hydrogel network. The presence of imine cross-links in the PDA/Ln-HAp@L hydrogel network can be confirmed by FTIR analysis, showing a distinct peak of C = N at approximately 1600 cm^–1^ in the FTIR spectra of PDA/Ln-HAp@L nanocomposite hydrogels (Figure S15).

**Figure 2:**
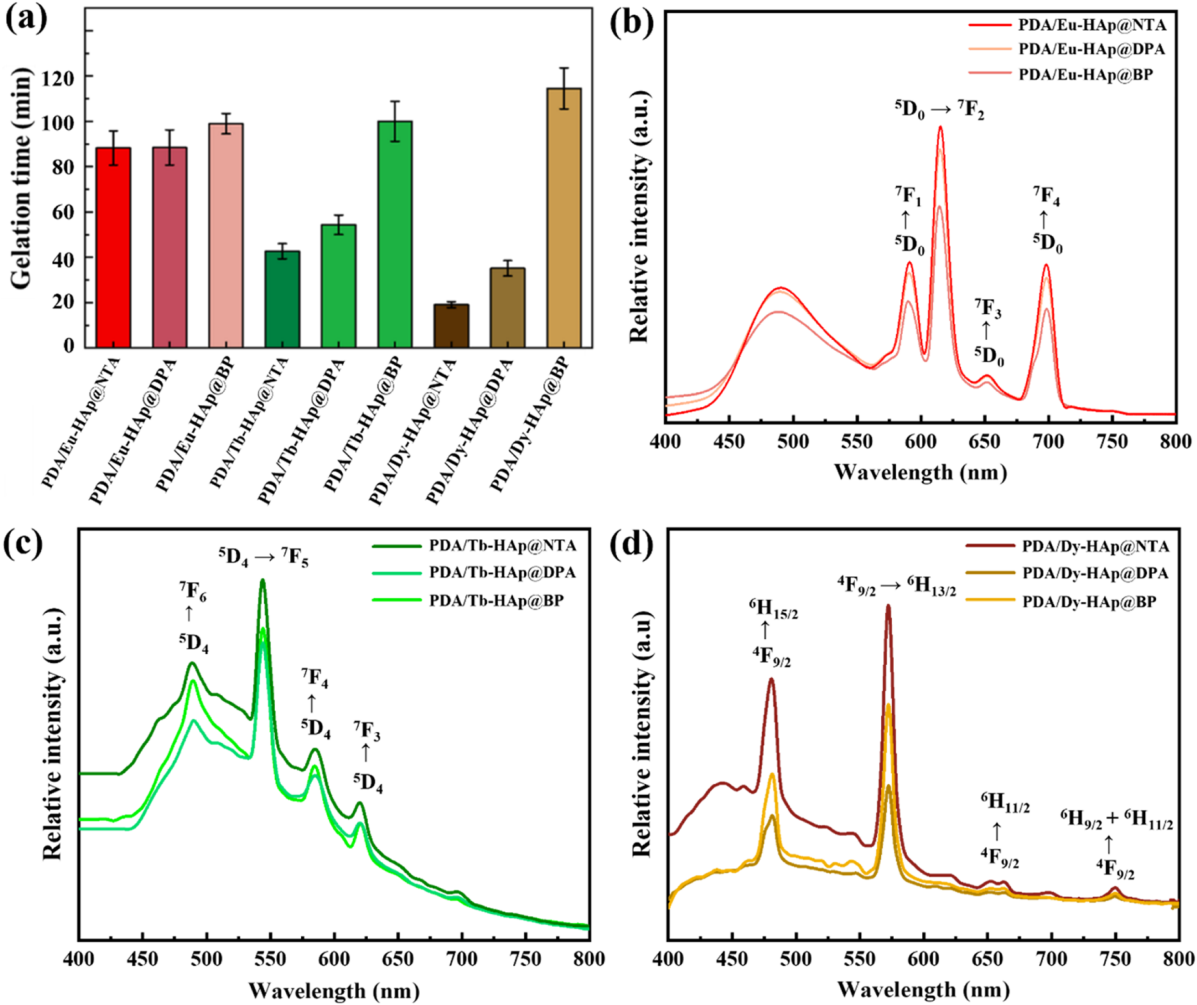
(a) Gelation times of the PDA/Ln-HAp@L hydrogels. Luminescence spectra of (b) PDA/Eu-HAp@L, (c) PDA/Tb-HAp@L, and (d)PDA/Dy-HAp@L hydrogels.

The gelation times of PDA/Ln-HAp@L nanocomposite hydrogels were compared through the oscillation time sweep by obtaining the gel points (cross points of *G*′ and *G*″) for each hydrogel. The results showed that PDA/Dy-HAp@NTA nanocomposite hydrogel presented the fastest gelation among the nine types of PDA/Ln-HAp@L nanocomposite hydrogels (Figures 2a and S16). In general, the gelation time of the metal–ligand coordination hydrogels is influenced by various factors, including the structure of the ligand,^48^ diameter of the metal ion,^49,50^ and metal–ligand stability.^51^ Here, fast gelation in the PDA/Dy-HAp@NTA hydrogels can be attributed to the rapid formation of a stable Dy-NTA coordination bond. The Dy^3+^ (*r* = 91.2 Å) ion possesses a smaller diameter compared to Eu^3+^ (*r* = 94.7 Å) and Tb^3+^ (*r* = 92.3 Å) ions,^52^ thus Dy^3+^ ions generated stronger bonding with coordination ligands to provide a stable amine-functionalized surface on HAp, and subsequently facilitating the formation of imine cross-links after interacting with the aldehydes of PDA. On the other hand, with the same Dy^3+^ ions in the Ln-HAp@L, the rapid gelation of the PDA/Dy-HAp@NTA hydrogel network also suggested that NTA exhibited a stronger coordination ability with lanthanide ions compared to DPA and BP.

The PDA/Ln-HAp@L hydrogels exhibited distinct luminescence under UV excitations (Figure S17), with PDA/Eu-HAp@L, PDA/Tb-HAp@L, and PDA/Dy-HAp@L showing red, green, and yellow-green luminescence, respectively. The luminescence properties of PDA/Ln-HAp@L hydrogels were also investigated (Figure 2b–d). The luminescence spectrum of the PDA/Eu-HAp@L hydrogels exhibited peaks at 591, 616, and 702 nm corresponding to the ^5^D_0_ → ^7^F*_j_* (*J* = 0–4) transitions of Eu^3+^ ions in Eu-HAp@L as well as a broad peak in the range of 400–500 nm associated with the PDA polymer (Figure S18). Similarly, the luminescence spectrum of the PDA/Tb-HAp@L hydrogels showed four distinct characteristic peaks at 488, 543, 583, and 621 nm, indicating transitions from the ^5^D_4_ to the ^7^F*_j_* levels (*J* = 6–3). In addition, PDA/Dy-HAp@L hydrogels exhibited four primary luminescence peaks (i.e., 481, 572, 657, and 450 nm), each associated with specific transitions from the ^4^F_9/2_ state to different ^6^H states. Therefore, incorporating Ln-HAp@L into the PDA matrix preserves their luminescence properties within the PDA/Ln-HAp@L nanocomposite hydrogels.

The PLQY of Ln-HAp@L hydrogels was also quantified and compared with those of their wet and dry state. The wet Ln-HAp@L hydrogels consistently showed a lower PLQY than the lyophilized Ln-HAp@L hydrogels (Table S3), which should be due to water interfering with the ligand-to-metal energy transfer. However, the reduction percentage in luminescence observed in the Ln-HAp@L hydrogel ranged from 15.4 to 26.3%, which was lower than that of other Ln-containing hydrogels that are more susceptible to water interference and showed a more significant decrease in luminescence when comparing their wet and dried states.^53^ Therefore, embedding the lanthanide ions within HAp can effectively reduce water interference to provide sufficient luminescence in the PDA/Ln-HAp@L hydrogels for luminescence-based applications (e.g., sensing and imaging).

**Figure 3:**
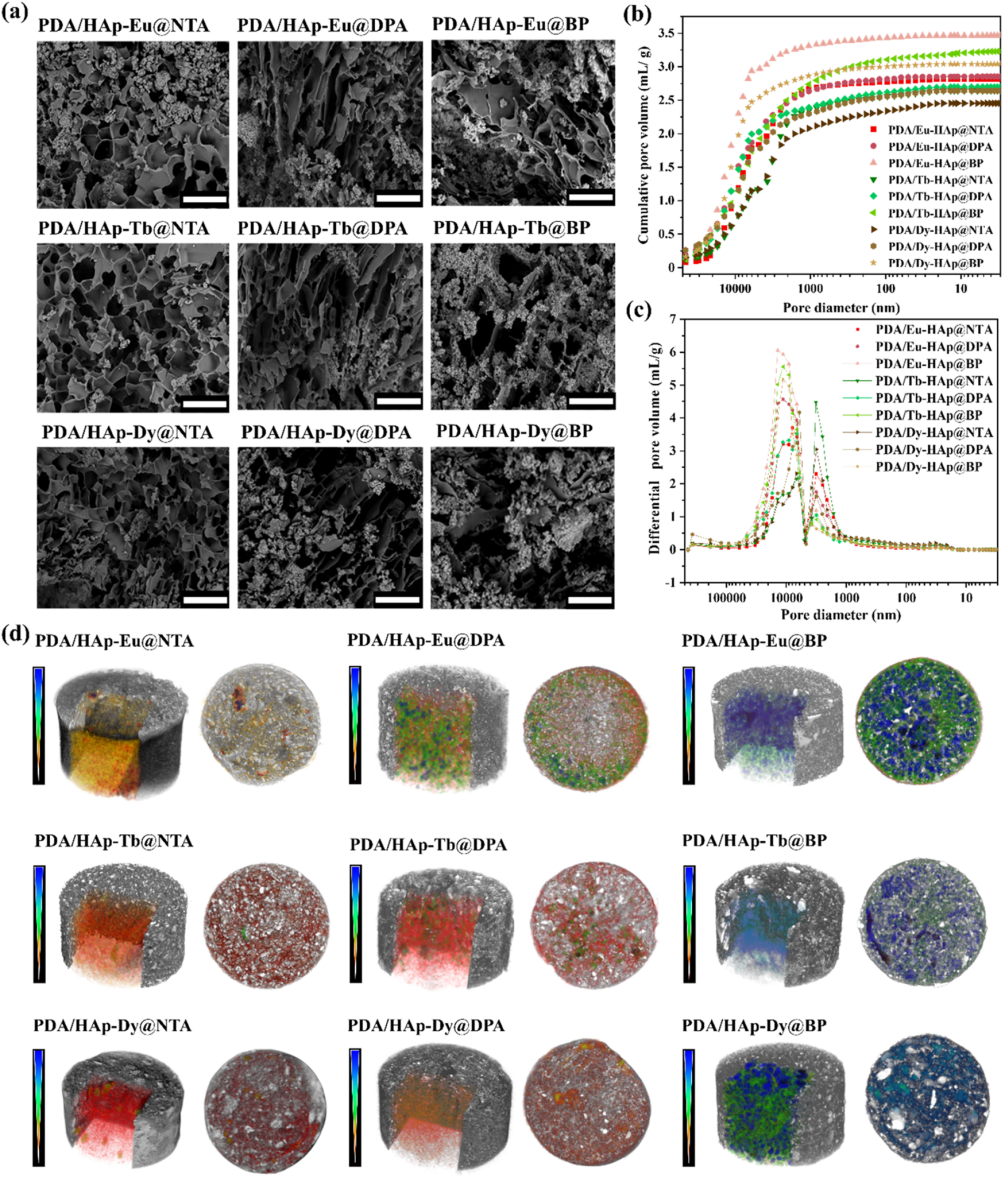
(a) Representative SEM images of PDA/Ln-HAp@L nanocomposite hydrogels. Scale bar = 100 μm. Pore size distributions were presented as (b) cumulative pore volume and (c) differential pore volume (d*V*/d*l*ogD) of PDA/Ln-HAp@L nanocomposite hydrogels. (d) Micro-CT images and cross-sectional views of PDA/Ln-HAp@L nanocomposite hydrogels. The pore sizes of nanocomposite hydrogels were indicated by using a color scale, with higher values on the color bar exhibiting larger pore sizes. It should be noted that this scale represents the relative measurements.

The porous microstructures of the PDA/Ln-HAp@L hydrogels were revealed under SEM (Figure 3a), and elemental mapping was also applied to confirm the presence and homogeneous distribution of lanthanide ions within the PDA/Ln-HAp@L hydrogel matrix (Figure S19). Mercury intrusion porosimetry (MIP) and microcomputed tomography (micro-CT) were further used to analyze the pore size distribution and porosity of the nanocomposite hydrogels (Figure 3b–d and Tables S4–S5). The results showed that the PDA/Ln-HAp@NTA hydrogel exhibited a smaller average pore diameter compared to that of PDA/Ln-HAp@DPA and PDA/Ln-HAp@BP hydrogels. For example, in the micro-CT analysis, the pore size in PDA/Eu-HAp@NTA hydrogel was 46.5 μm, which was smaller than that of PDA/Eu-HAp@DPA (54.0 μm) and PDA/Eu-HAp@BP (54.3 μm) hydrogels. On the other hand, for hydrogels with the same chelate ligand, PDA/Dy-HAp@L hydrogels showed smaller pore sizes than those in PDA/Eu-HAp@L and PDA/Tb-HAp@L hydrogels. With the micro-CT analysis, the pore size in PDA/Dy-HAp@NTA hydrogel was 27.7 μm, which was smaller than that of PDA/Tb-HAp@NTA hydrogel (44.3 μm) and PDA/Eu-HAp@NTA hydrogel (46.5 μm). In particular, micro-CT also revealed that all PDA/Ln-HAp@L nanocomposite hydrogels exhibited a greater proportion of open porosity than closed porosity, suggesting this structural configuration can enhance the swelling capabilities of hydrogels as open pores with open faces facilitate fluid flow and penetration.^54^ The small average pore diameter of the PDA/Dy-HAp@NTA hydrogel should be related to a stronger interaction between Dy^3+^ ions and ligands, which contributed to the smaller radius of Dy^3+^ ions compared to Eu^3+^ and Tb^3+^. Among these nine types of hydrogels, PDA/Eu-HAp@BP hydrogel exhibited the largest pores, which could be attributed to the presence of short and rigid alkyl structure –NH_2_-(CH)– in the BP ligands to reduce the cross-linking efficiency between Eu-HAp@BP and PDA.

### Rheological and Mechanical Properties of Hydrogels

The impact of chelate ligands and lanthanide ions on the rheological properties of the nine types of PDA/Ln-HAp@L hydrogels was systematically investigated using oscillatory time sweep and strain sweep. In the oscillatory time sweep, all PDA/Ln-HAp@L hydrogels presented rheological stability over time (Figure S20). Oscillation strain sweep revealed the rheological properties of the PDA/Ln-HAp@L hydrogel network; for hydrogels with the same lanthanide ion, the PDA/Ln-HAp@NTA hydrogel exhibited the largest *G*′ compared to PDA/Ln-HAp@DPA and PDA/Ln-HAp@BP hydrogels (Figure S21 and Table S6). For example, the *G*′ of PDA/Eu-HAp@NTA hydrogel was 1265.97 ± 39.49 Pa, which was higher than the *G*′ of PDA/Eu-HAp@DPA (*G*′ = 757.26 ± 46.19 Pa) and PDA/Eu-HAp@BP (*G*′ = 39.02 ± 2.84 Pa) hydrogels (Table S6). On the other hand, for hydrogels with the same coordination ligand, PDA/Dy-HAp@L hydrogel showed a higher *G*′ than PDA/Eu-HAp@L and PDA/Ln-HAp@L hydrogels. For example, the *G*′ of the PDA/Dy-HAp@NTA hydrogel was 8588.26 ± 565.02 Pa, which was higher than the *G*′ of PDA/Eu-HAp@NTA (*G*′ = 1265.97 ± 39.49 Pa) and PDA/Tb-HAp@NTA (*G*′ = 3464.19 ± 169.74 Pa) hydrogels (Table S6).

It was noticed that PDA/Eu-HAp@BP hydrogel was extremely soft, which was likely attributable to a short alkyl structure of BP that restricts flexibility during the cross-linking process. In contrast, NTA, having a long alkyl side chain with flexible moieties as well as electron-withdrawing carboxylate chelating groups, presented the ability to promote the formation of more imine bonds within the network to enhance the rheological properties of the hydrogels. To demonstrate the effect of ligand flexibility, *N*′,*N*′-bis(pyridin-2-ylmethyl)ethane-1,2-diamine (sDPA) with a dipyridine structure and a short and rigid skeleton was synthesized, which had the same chelating groups to the DPA ligand. The sDPA ligand was incorporated into Eu-HAp to generate Eu-HAp@sDPA (with an amine amount of 0.89 mmol/g) and then subsequently mixed with PDA to form PDA/Eu-HAp@sDPA hydrogel. The PDA/Eu-HAp@sDPA hydrogel showed a liquid-like appearance compared to the more solid-like PDA/Eu-HAp@DPA hydrogel (Figure S22a). The rheological results also showed that the *G*′ of PDA/Eu-HAp@sDPA hydrogel (∼50 Pa) was significantly lower than that of PDA/Eu-HAp@DPA hydrogel (∼1100 Pa) (Figure S22b). Therefore, this result highlights the critical role of ligand flexibility in contributing to cross-linking the hydrogel network, where a flexible chain is needed to provide sufficient cross-linking density.

**Figure 4:**
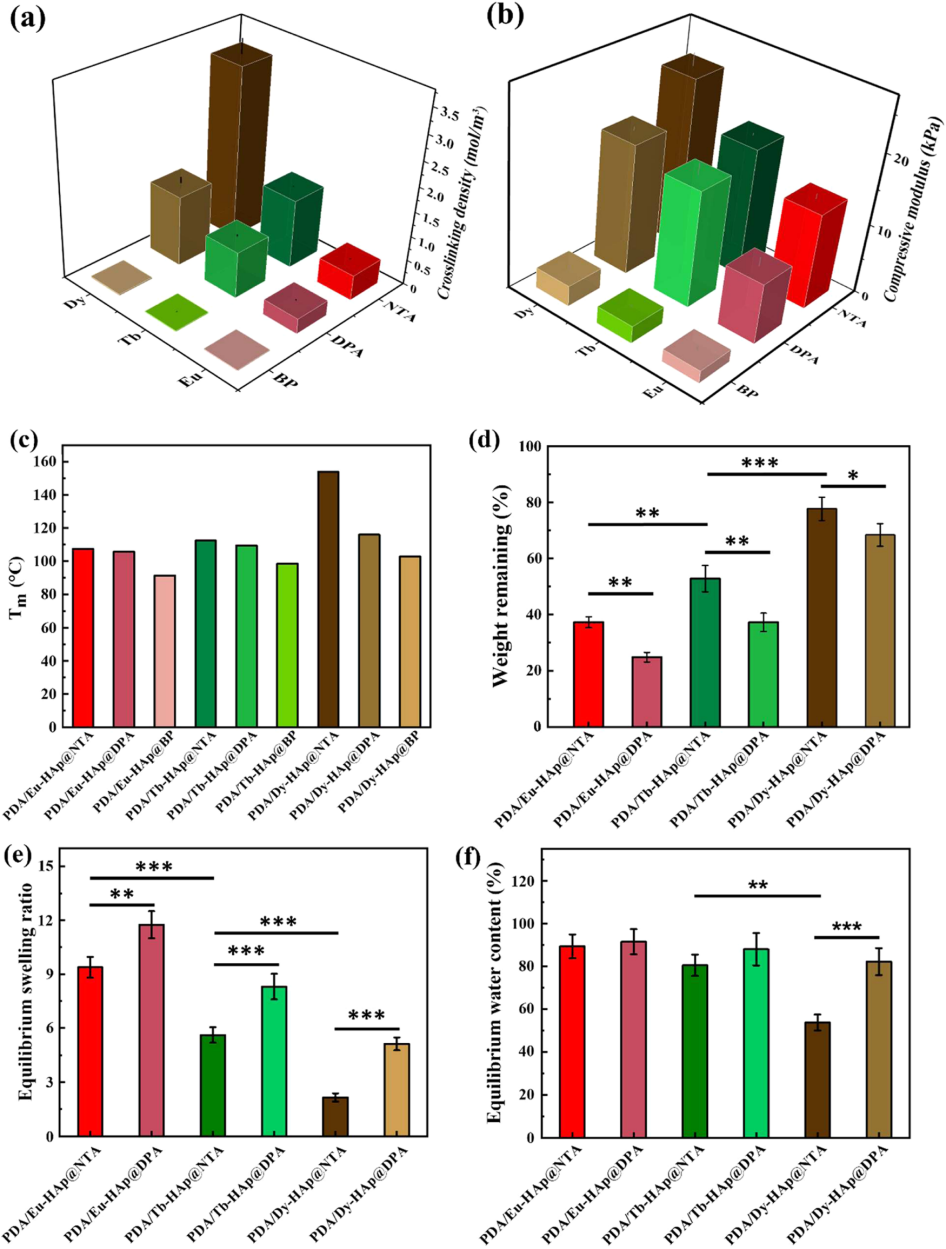
(a) Cross-linking densities and (b) compression moduli of PDA/Ln-HAp@L hydrogels. (c) *T*_m_ values of PDA/Ln-HAp@L hydrogels. (d) Weight remaining of PDA/Ln-HAp@L hydrogels after immersion for 24 h. (e) Equilibrium swelling ratio and (f) equilibrium water content of PDA/Ln-HAp@L hydrogels after immersion for 2 h. Significance was set at *p* < 0.05 with *, **, or *** indicating *p* < 0.05, 0.01, or 0.001, respectively.

The cross-linking density of the PDA/Ln-HAp@L hydrogels can be calculated using the theory of rubber elasticity: *G*′ = vRT, where *G*′ represents for storage modulus in Pa, v is the cross-linking density in moles of elastically effective network chains per m^3^, R is the gas constant (8.314 J K^–1^ mol^–1^), and *T* is the temperature (298 K).^21,55^As an example, the cross-linking densities of the PDA/Eu-HAp@NTA, PDA/Eu-HAp@DPA, and PDA/Eu-HAp@BP hydrogels were determined to be ∼0.51, 0.31, and 0.02 mol/m^3^, respectively (Figure 4a and Table S6). Additionally, the flow points (the strain at which *G*′ equals *G*″) for PDA/Eu-HAp@NTA, PDA/Eu-HAp@DPA, and PDA/Eu-HAp@BP hydrogels were ∼42, 24, and 11%, respectively. This phenomenon indicated that hydrogels with higher cross-linking densities exhibited more rigid structures, subsequently fracturing under larger deformations. Notably, PDA/Dy-HAp@L hydrogels generally performed higher cross-linking densities and flow points than the PDA/Eu-HAp@L and PDA/Tb-HAp@L hydrogels.

The mechanical properties of PDA/Ln-HAp@L hydrogels were investigated by using the compression test (Figures 4b and S23, Table S7). The PDA/Dy-HAp@NTA hydrogel presented the highest mechanical strength of ∼24 kPa among the nine types of hydrogels. Conversely, PDA/Ln-HAp@BP hydrogels showed poorer mechanical properties (∼1.76 kPa for PDA/Eu-HAp@BP) compared to the PDA/Ln-HAp@NTA and PDA/Ln-HAp@DPA hydrogels. Thus, the compression moduli of PDA/Ln-HAp@L hydrogels presented a trend similar to the *G*′ of hydrogels observed in the rheological studies. Both rheological and compression analyses offer valuable insights into the cross-linking characteristics and mechanical properties of PDA/Ln-HAp@L hydrogels, highlighting the importance of selecting suitable ligands and lanthanide ions to optimize the properties of hydrogels.

### Stability and Swelling Tests of Hydrogels

The thermal stability of the PDA/Ln-HAp@L hydrogels was evaluated by using differential scanning calorimetry (DSC) (Figures 4c and S24). The crystal melting temperatures (*T*_m_) of PDA/Ln-HAp@L hydrogels were correlated to the types of the Ln-HAp@L cross-linked hydrogel network, showing the *T*_m_ in the range of 90–155 °C. Among the hydrogels, the PDA/Dy-HAp@NTA hydrogel exhibited the highest *T*_m_ (154.0 °C), while the PDA/Eu-HAp@BP hydrogel showed the lowest *T*_m_ (91.5 °C). In addition to the thermal stability, the hydrogels were immersed in the aqueous solution to reveal their construct integrity. In general, the imine cross-links in the hydrogel network show a pH-dependent hydrolytic behavior. Specifically, imine bonds are cleaved easily under acidic conditions (pH< 5), whereas under neutral to slightly basic conditions (pH> 9), the hydrolysis rate is considerably reduced.^56^ It was noticed that PDA/Ln-HAp@BP hydrogels with large pore sizes and insufficient cross-linked networks were highly unstable in aqueous solution as they collapsed in water within seconds (Figure S25). The immersion results showed that PDA/Dy-HAp@L hydrogels exhibited a degradation rate slower than that of the PDA/Eu-HAp@L and PDA/Tb-HAp@L hydrogels (Figure 4d). Additionally, the PDA/Ln-HAp@NTA hydrogels also showed a lower degradation rate than the PDA/Ln-HAp@DPA hydrogels. Specifically, PDA/Dy-HAp@NTA hydrogel retained the highest structural integrity of 80% after immersion for 24 h, while the PDA/Eu-HAp@DPA hydrogel showed the least structural retention of only 25%. Both DSC and immersion tests suggested that hydrogels with a higher cross-link density possess more rigid structures, which increased their thermal stability and reduced their degradation rates. Dextran and HAp used in this study are biodegradable materials, and the imine cross-linking chemistry used in our system also allows the nanocomposite hydrogels to be degraded over time under physiological conditions. Thus, a detailed life cycle assessment of nanocomposite hydrogels will be considered for future work to provide a more fundamental understanding of their environmental impact and sustainability.

The swelling behaviors of the PDA/Ln-HAp@DPA and PDA/Ln-HAp@NTA hydrogels were examined by submerging the lyophilized hydrogels in water at 37 °C. The results showed that all six hydrogels reached equilibrium within 2 h of immersion (Figures 4e,4f and S26). The PDA/Eu-HAp@DPA hydrogel showed the highest swelling ratio (11.8) and water content (91.5%) after immersion for 2 h, while the PDA/Dy-HAp@NTA hydrogel exhibited the lowest swelling ratio (2.2) and water content (53.7%). These swelling results reflected the intricate interplay between the pore size and swelling behavior of the hydrogels. PDA/Dy-HAp@NTA hydrogel possessed the smallest pore size to resist water infiltration, presenting poor swelling ability and preserving the structural integrity of PDA/Dy-HAp@NTA hydrogel during immersion.

### Computational Investigations of Lanthanide–Ligand Complexes

The dynamic cross-linking networks in the PDA/Ln-HAp@L systems are established through Ln···L interactions at the surface of Ln-HAp and the imine bonds at the cross-linking points formed by the aldehydes on PDA and the amines at the terminal of chelating ligand. As one of the key components to determine the cross-linking density and strength that are readily tunable leading to a broad range of mechanical properties, density functional theory (DFT) calculations were performed to elucidate the Ln···L interactions in the hydrogels.

**Scheme 2:**
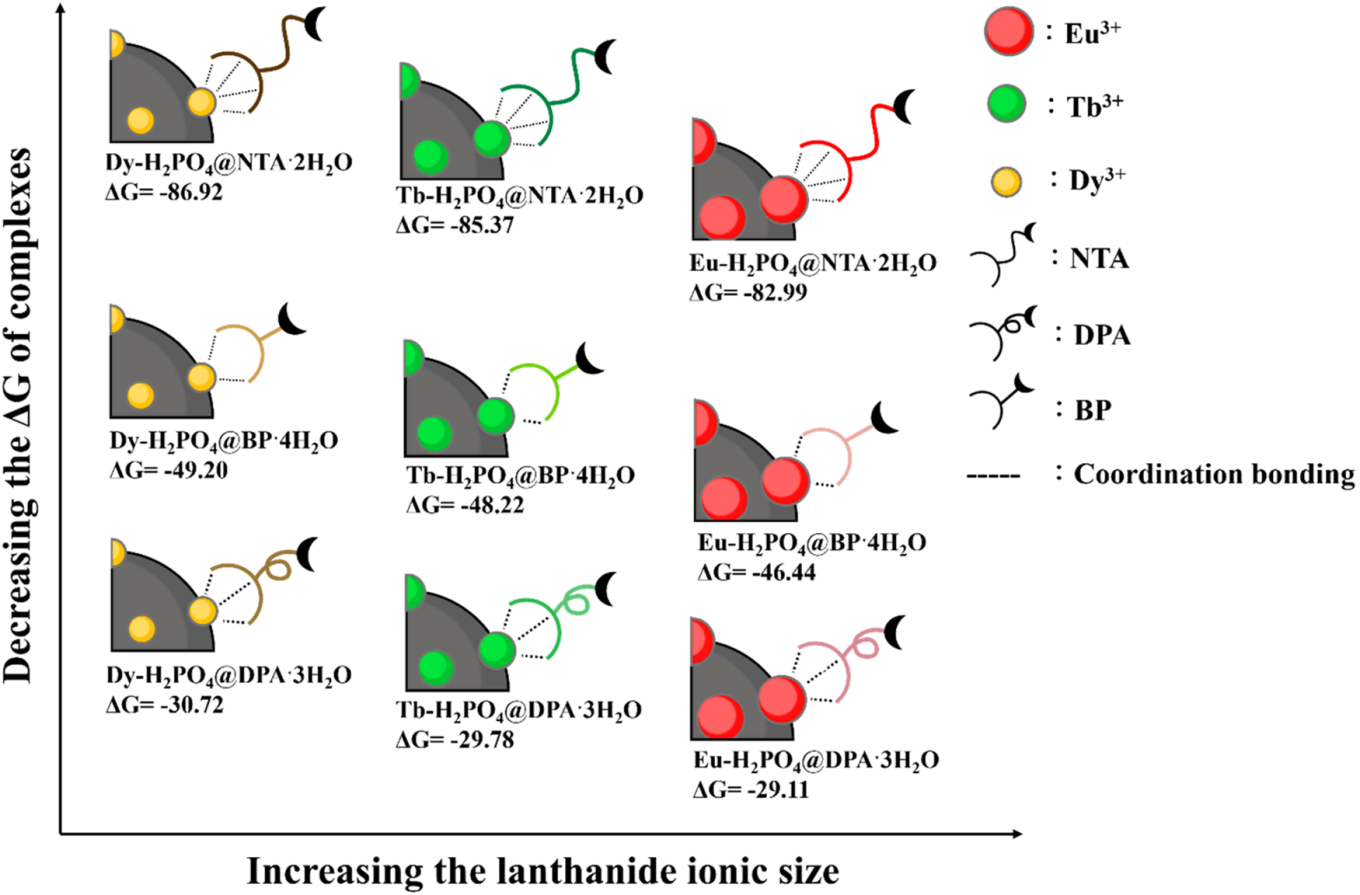
Illustrations of the Relative Calculated Δ*G* of the Ln-H_2_PO_4_@L·*x*H_2_O Complexes on the HAp Surface and Their Correlation with the Lanthanide Ionic Size

Here, the Ln···L model systems were constructed with a 1:1 ratio of the lanthanide ions (i.e., Eu^3+^, Tb^3+^, or Dy^3+^) and chelating ligand (i.e., NTA, DPA, or BP), with the dihydrogen phosphate (H_2_PO_4_^–^) groups from HAp and the surrounding water molecules (H_2_O) also coordinated to lanthanides to achieve an eight-coordination structure, forming the formula Ln-H_2_PO_4_@L·*x*H_2_O (Figure S27). The alkyl side chain of the ligands has been replaced with a methyl group to replicate the electronic effects on the complexation core while minimizing potential disruptions from conformational flexibility and allowing for a focused analysis of the Ln···L interaction. Details of the model systems and calculation methods can be found in the Computational Methods section. The DFT-optimized structures of the nine Ln@L systems are shown in Scheme 2, and detailed structural information is included in the Supporting Information (Table S8).

The DFT calculation revealed that the NTA exhibited the strongest Gibbs binding free energy (Δ*G*) toward lanthanide ions among the three ligands **(**Scheme 2), such as Dy^3+^···NTA (−86.92 kcal/mol) > Dy^3+^···BP (−49.20 kcal/mol) > Dy^3+^···DPA (−30.72 kcal/mol), supporting the observed enhanced mechanical strength in the experiments (Figure 4a,b and Tables S6–S7). On the other hand, the calculated Δ*G* of BP and DPA ligands toward lanthanide ions did not align with the order of the strength of the observed mechanical properties, in which BP exhibited a stronger Ln···L bonding (lower binding energy) but a weaker mechanical strength and smaller cross-linking density (Figure 4a,b and Tables S6–S7). This highlights the importance of another characteristic, i.e., the length of the alkyl side chain of the ligands (see Scheme 1b for the ligand structures), in the cross-linking efficiency. The short side chains of the BP ligands at the surface of a Ln-HAp@BP nanoparticle limit the nanomaterial’s ability to effectively reach multiple aldehyde functional groups of PDA (the cross-linking points), diminishing the feasibility of constructing dense cross-linking networks in the hydrogels. Moreover, the conformational flexibility of the ligands with longer side chains offers additional degrees of freedom to accommodate tensile/compressive strain, contributing to improved mechanical properties compared to rigid short side chains with only one C–C bond in BP. The importance of the length of the side chain has been confirmed by sDPA, where a shorter side chain in the ligand was used for hydrogel construction. and the *G*′ of PDA/Eu-HAp@sDPA (∼50 Pa) was much lower than that of PDA/Eu-HAp@DPA (∼1100 Pa) (Figure S22b).

While keeping the ligand constant, the calculated Δ*G* showed the binding strength Dy^3+^···L (−86.92 kJ/mol) > Tb^3+^···L (−85.37 kJ/mol) > Eu^3+^···L (−82.99 kJ/mol) (Scheme 2), which is consistent with the observed trend in the mechanical strength of the materials (Figure 4a,b, and Tables S6–S7). However, the calculated Δ*G* differences were relatively small. This suggests the need to consider other factors to fully explain the larger mechanical property variations observed with different lanthanide ions. All in all, the Ln···L interactions, along with the imine bonds, compose the cross-linking networks in the hydrogels, playing a significant role in determining the mechanical properties. However, other characteristics, such as side chain lengths of the ligand, have to be taken into account for a complete picture.

Taken together, nine types of Ln-HAp@L and PDA/Ln-HAp@L nanocomposite hydrogels with different structures and properties were demonstrated here by simply varying the types of lanthanide ions and capping ligands (Tables S10–S12). Particularly, incorporating the lanthanide ions into the Ln-HAp@L cross-linkers can significantly minimize water interference, maintaining their luminescent properties within the PDA/Ln-HAp@L nanocomposite hydrogels. Additionally, the luminescence spectrum can be modified by altering the lanthanide types.

**Scheme 3:**
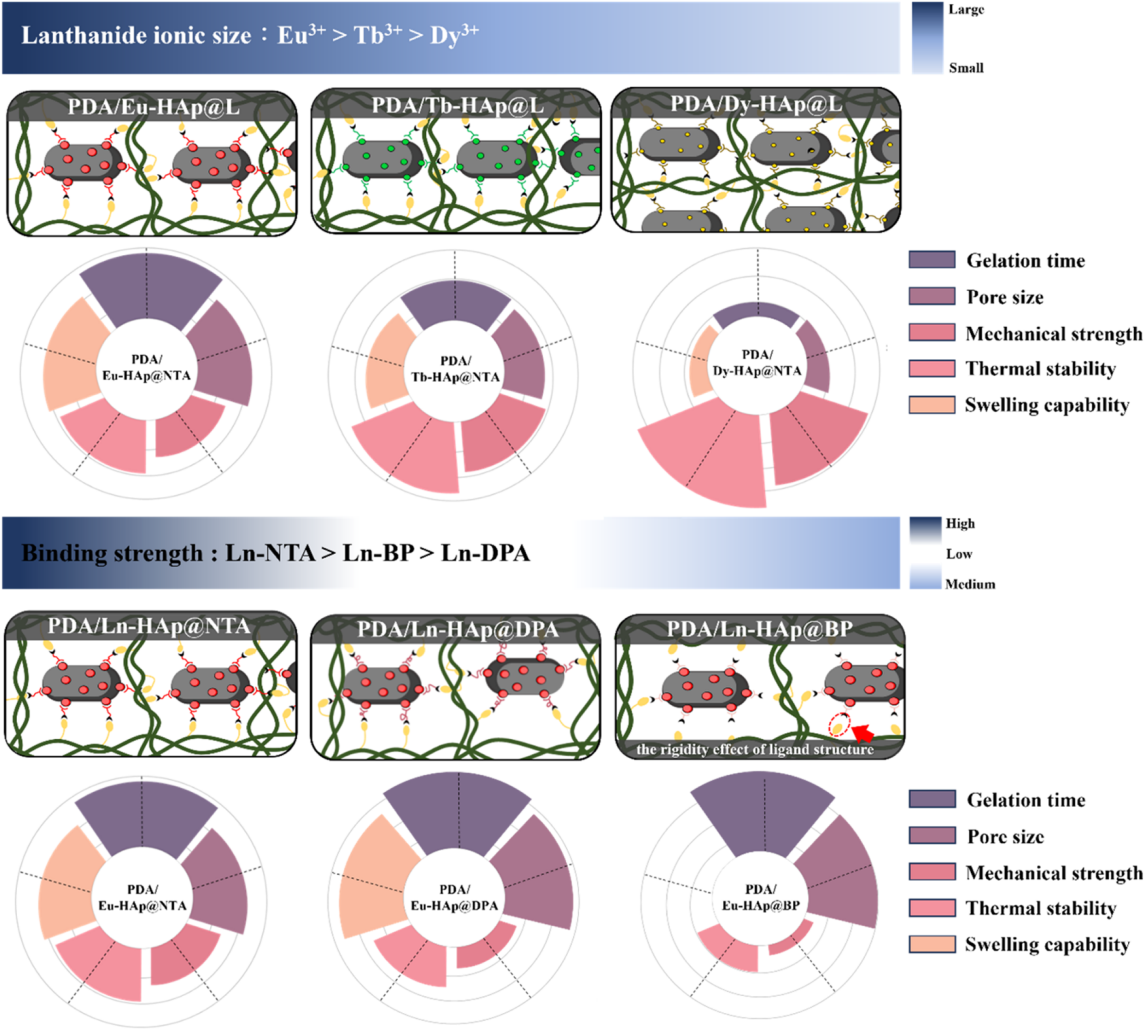
Schematic Illustrations of the Influence of Ln-HAp@L on the Microstructures and Properties of PDA/Ln-HAp@L Hydrogels

The role of different lanthanide ions and surface capping ligands in the gelation time, pore sizes, mechanical strength, thermal stability, and swelling capability of the PDA/Ln-HAp@L hydrogels is schematically summarized in Scheme 3. The radial diagrams are also provided to quantitatively illustrate the relative performance of PDA/Ln-HAp@L hydrogels. For the effect of lanthanide ions, PDA/Dy-HAp@L hydrogels exhibited faster gelation time, smaller pore size, better mechanical strength, higher thermal stability, and lower swelling ratio compared to PDA/Tb-HAp@L and PDA/Eu-HAp@L hydrogels. These superior properties of PDA/Dy-HAp@L hydrogels should be due to the stronger coordination between Dy^3+^ ions and ligands, which is favored by the smaller ionic radius of Dy^3+^ ions compared to Eu^3+^ and Tb^3+^ ions. The improved stability of Dy-ligand coordination creates a stable amine-functionalized surface on HAp, increasing the number of imine cross-links within the network.

As for the surface chelating ligands, PDA/Ln-HAp@NTA hydrogel exhibited faster gelation time, smaller pore sizes, better mechanical strength, higher thermal stability, and lower swelling ratio compared to PDA/Ln-HAp@DPA and PDA/Ln-HAp@BP hydrogels. This mechanical enrichment of PDA/Ln-HAp@NTA hydrogels is probably due to the lower Gibbs energy of the Ln-NTA complex as the overall stability of the Ln-L complexes showed the binding strength trend of Ln-NTA > Ln-BP > Ln-DPA based on the computational simulation. Therefore, the Ln-NTA complex generates a stable amine-functionalized surface on HAp to promote the formation of a larger number of imine bonds at the PDA-HAp interface. Conversely, the short and rigid alkyl side chain of BP prevents effective cross-linking between PDA and Ln-HAp@BP, resulting in larger pores and poorer mechanical properties of PDA/Ln-HAp@BP hydrogels. Overall, among the nine types of PDA/Ln-HAp@L hydrogels, the strong binding affinity between Dy^3+^ ions and NTA, as well as the flexible amine-terminated side chain of NTA, facilitated the cross-linking between Dy-HAp@NTA and PDA, generating the densest network, highest mechanical strength, and best stability in the PDA/Dy-HAp@NTA hydrogel. These results emphasize that both the size of the lanthanide ions and the structural properties of the chelating ligand are critical parameters for tuning the properties of the luminescent hydrogels. Therefore, our work demonstrates a promising approach to fine-tune the lanthanide–ligand pairs on the nanomaterial cross-linkers to develop nanocomposite hydrogels with precisely controlled structural, mechanical, and optical properties.

DFT calculation was performed to further explain the experimental results of the PDA/Ln-HAp@L hydrogels at the molecular level. DFT results showed that the Dy-NTA complex possessed a binding energy of −47.5 kcal/mol, which was significantly lower than the values calculated for the Eu--NTA (−32.1 kcal/mol) and Tb-NTA (−30.7 kcal/mol) complexes. This quantitative difference in binding energies supported the experimental observation that the Dy-NTA pair led to a higher cross-linking density determined by the rheological data, showing a cross-linking density of 3.46 mol/m^3^ for PDA/Dy-HAp@NTA hydrogel compared to only 0.51 mol/m^3^ for PDA/Eu-HAp@NTA hydrogel. The stronger binding strength of the Dy-NTA complex correlated well with the observed fast gelation kinetics and the improved mechanical properties of the corresponding hydrogels. Therefore, the computational models suggested that the binding strength and stability of the lanthanide–ligand complex played crucial roles in determining the mechanical features of the hydrogel network. This synergy between experimental and theoretical results reinforces the critical importance of tailoring metal–ligand interactions to design advanced nanocomposite hydrogels.

### Self-Healing, Shear-Thinning, and Injectable Behaviors of Hydrogels

**Figure 5:**
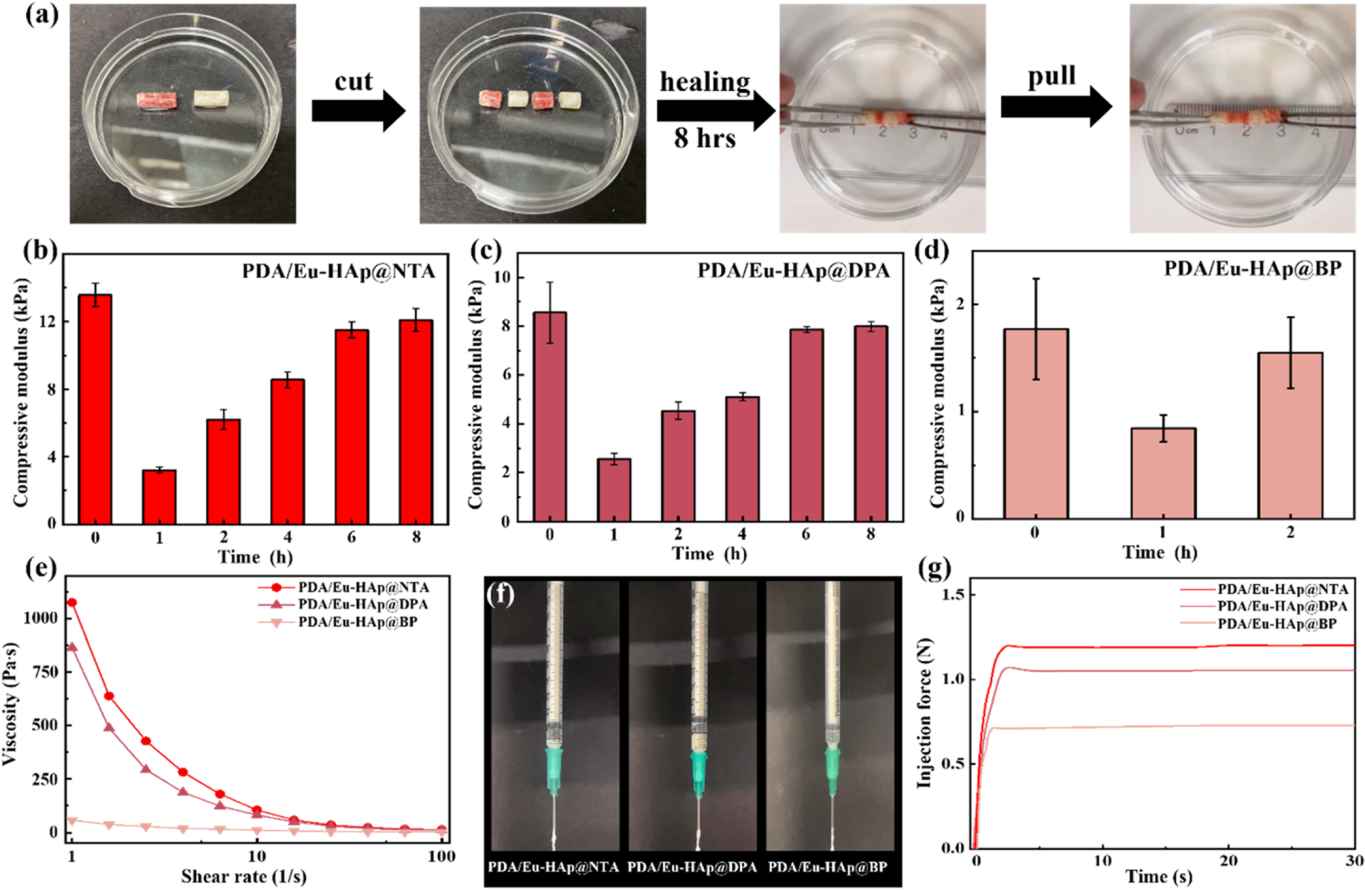
Self-healing, shear-thinning, and injectable behaviors of PDA/Eu-HAp@L hydrogels. (a) Demonstration of the self-healable PDA/Eu-HAp@NTA hydrogel and (b–d) the compressive modulus of hydrogel before and after healing for different periods. (e) Continuous flow sweeps of PDA/Eu-HAp@L hydrogels. (f) Demonstrations of the injectability of PDA/Eu-HAp@L hydrogels. (g) Injection force profiles of PDA/Eu-HAp@L hydrogels.

The self-healing, shear-thinning, and injectable features of PDA/Ln-HAp@L hydrogels were also demonstrated here as dynamic imine cross-links were formed at the interface between HAp and PDA within the network. PDA/Eu-HAp@L hydrogels served as representative samples to assess the recovery capability of PDA/Ln-HAp@L hydrogels through a pulse deformation process, alternating between low (1%) and high (500%) strain during a continuous step strain sweep (Figure S28). At high strain, the *G*″ exceeded the *G*′, indicating the polymer network disruption. When the strain amplitude was reduced back to 1%, *G*′ and *G*″ quickly returned to their initial values, showing the self-healing properties of PDA/Eu-HAp@L hydrogels. PDA/Eu-HAp@NTA hydrogel was further selected as a representative example to demonstrate the self-healing behavior of the hydrogel in macroscale, showing that the two pieces of cut PDA/Eu-HAp@NTA hydrogel were reunited after 24 h (Figure 5a). A compression test revealed that the recovery efficiency of the PDA/Ln-HAp@L hydrogels was time-dependent (Figures 5b–d and S29). For instance, the recovery efficiency of the PDA/Eu-HAp@NTA hydrogel was 45.7, 63.0, 84.7%, and 88.7% recovery after 2, 4, 6, and 8 h, respectively (Figure 5b). It was also noticed that PDA/Eu-HAp@DPA and PDA/Eu-HAp@BP hydrogels presented a faster recovery time compared to PDA/Eu-HAp@NTA hydrogel, suggesting enhanced mechanical strength of the cross-linked network may impede self-repair mechanisms.^57^

The rheological properties, particularly viscosity behavior, are pivotal in determining the suitability of hydrogels for injection-related applications, such as drug delivery and 3D printing. Here, PDA/Eu-HAp@L hydrogels presented shear-thinning behavior as their viscosities decreased once the shear rate increased. (Figure 5e) The power law index was further used to quantitatively analyze this shear-thinning behavior with the equation τ = κγ*^n^*, where τ is the shear stress, κ is the consistency index, γ is the shear rate, and n is the flow behavior index.^58^ Power law fluids can be divided into three different types of fluids based on the value of their flow behavior index. Pseudoplastic fluids (*n* < 1) exhibit shear-thinning properties where the viscosity decreases as the shear rate increases. Newtonian fluids (*n* = 1) maintain a constant viscosity regardless of changes in the shear rate. Dilatant fluids (*n* > 1) exhibit shear-thickening properties where the viscosity increases with increasing shear rate. The power law indices of the PDA/Eu-HAp@NTA, PDA/Eu-HAp@DPA, and PDA/Eu-HAp@BP hydrogels were 0.005, 0.004, and 0.297, respectively, confirming their inherent shear-thinning properties (Figure S30). The lower the power law index, the more shear-thinning the liquid was. The PDA/Eu-HAp@BP hydrogel showed favorable shear-thinning characteristics, however, its low compressive modulus (∼1.2 kPa) indicated weak mechanical properties, rendering it unsuitable for injection applications. In contrast, the PDA/Eu-HAp@DPA hydrogel, with its superior shear-thinning behavior and adequate mechanical performance (∼8.6 kPa), emerged as a more suitable candidate for applications.

The injectability of PDA/Eu-HAp@L hydrogels was also demonstrated visually, showing the smooth edges and continuous flow when the hydrogels were injected through the syringe with no signs of breakage (Figure 5f). The continuous flow sweeps, shear stress–shear rate curves, and demonstrations of the injectability of PDA/Tb-HAp@L and PDA/Dy-HAp@L hydrogels are also shown in Figure S31. An inversion test further highlighted the time-dependent shear-thinning behavior of the viscoelastic material. The prepared hydrogel did not flow under gravity even after 24 h, an essential property for ensuring injectability under applied yield stress while maintaining shape at defect sites (Figure S32). The injectability of PDA/Eu-HAp@L hydrogels was further compared by measuring the force required for extrusion, where the hydrogels were loaded into a syringe with an 18G needle and extruded at a flow rate of 1 mL/min. The injection forces for the PDA/Eu-HAp@NTA, PDA/Eu-HAp@DPA, and PDA/Eu-HAp@BP hydrogels were 1.20, 1.05, and 0.73 N, respectively (Figure 5g). These injection results were correlated to the microstructures and mechanical properties of the hydrogels, showing that a higher force was needed to extrude the PDA/Eu-HAp@NTA hydrogel with a dense and strong network. Overall, the dynamic imine cross-linked hydrogel network possessed self-healing, shear-thinning, and injectable properties highly desirable for manufacturing (e.g., 3D printing) and minimally invasive therapy.

### Differentiation of Volatile Organic Compounds Using PDA/Eu-HAp@NTA Lyophilized Hydrogels

Lanthanide-containing luminescent materials have been widely used in detecting volatile organic compounds (VOCs), such as tetrahydrofuran (THF),^59^ ammonia (NH_3_),^60^ and formaldehyde (FA).^61^

**Figure 6:**
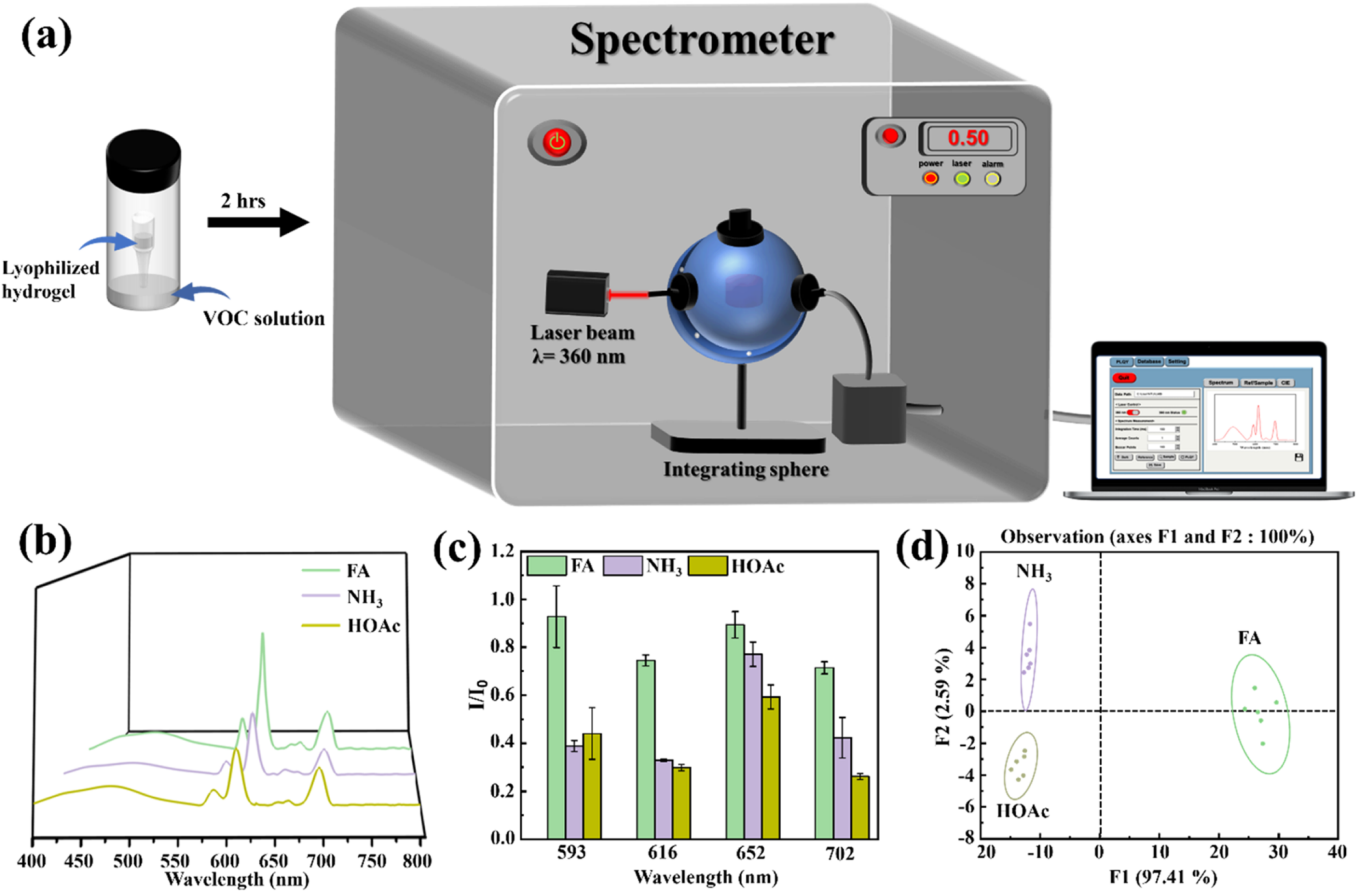
(a) Schematic illustration of sensing VOCs using the PDA/Eu-HAp@NTA lyophilized hydrogel. (b) Luminescent spectra of the PDA/Eu-HAp@NTA lyophilized hydrogel for sensing different VOCs in the gas phase (λ_ex_= 360 nm). (c) Signal patterns of the luminescent intensity variety (*I*/*I*_0_) of the PDA/Eu-HAp@NTA lyophilized hydrogel for sensing VOCs. (d) Canonical score plot for VOC sensing of the PDA/Eu-HAp@NTA lyophilized hydrogel obtained from LDA.

Given that VOC sensing was conducted using the luminescent changes of hydrogels, the PDA/Eu-HAp@NTA hydrogel was selected due to its superior PLQY compared to others. PDA/Eu-HAp@NTA hydrogels were freeze-dried to create lyophilized hydrogels to differentiate the VOCs. The PDA/Eu-HAp@NTA lyophilized hydrogel was placed in a vial containing different VOCs (i.e., ammonia (NH_3_), acetic acid (HOAc), and formaldehyde (FA)), and the luminescence changes of the lyophilized hydrogel were analyzed using a spectrometer (Figure 6a). The results showed that the luminescence of the PDA/Eu-HAp@NTA lyophilized hydrogel diminished after exposure to VOCs (Figure 6b), creating distinct response patterns for each VOC (Figure 6c). Notably, by analyzing the intensities of the luminescent peaks with linear discriminant analysis (LDA), it was demonstrated that the three VOCs could be distinguished from one another (Figure 6d).

The luminescence intensity of PDA/Eu-HAp@NTA lyophilized hydrogel is considerably quenched in the presence of acid vapors as protons could break the chelation between Eu^3+^ and NTA and impair the sensitization of NTA to Eu^3+^.^60^ The quenching of the luminescence of the PDA/Eu-HAp@NTA lyophilized hydrogel upon exposure to FA^61^ and NH_3_^62^ can be attributed to competing coordination interactions that interfere with the antenna effect essential for luminescence. Specifically, FA or NH_3_ molecules are likely to compete with NTA for the coordination sites on the Eu^3+^ ions, leading to the displacement of NTA and subsequent failure of the antenna effect, which, in turn, quenches luminescence. To confirm whether FA and NH_3_ can replace the NTA of the PDA/Eu-HAp@NTA lyophilized hydrogel during sensing, the lyophilized hydrogel was immersed in the NTA solution to determine if its luminescence could be restored. The results indicated that after sensing FA or NH_3_, immersing the PDA/Eu-HAp@NTA lyophilized hydrogel in the NTA solution significantly increased the luminescence (Figure S33). This increase demonstrated that the Eu^3+^-NTA coordination was reversible, implying that the PDA/Eu-HAp@NTA lyophilized hydrogel sensor can be regenerated by allowing Eu^3+^ ions to recoordinate with an excess of NTA. Thus, these findings highlight the reversible coordination dynamics of the Eu^3+^-NTA complex, supporting the recycling capability of this lyophilized hydrogel sensor and offering a promising path for sustainable luminescent sensing materials.

To examine the behavior of the PDA/Eu-HAp@NTA lyophilized hydrogel in the presence of other common VOCs, it was also exposed to chloroform (CHCl_3_), dichloromethane (DCM), acetone, ethyl acetate (EA), ethanol (EtOH), carbon disulfide (CS_2_), and toluene, where the results also showed a decrease in luminescence intensity (*I*/*I*_0_) (Figure S34). As a result, the lyophilized PDA/Eu-HAp@NTA hydrogel generally exhibited decreased luminescence when detecting VOCs. The gas molecules might interfere with the coordination of the lanthanides within the polymeric network, diminishing their luminescence effectiveness. Therefore, the ability to attenuate water-induced luminescence quenching by embedding lanthanide ions in a robust HAp nanomaterial matrix enhances the reliability of the resulting nanocomposite hydrogels for VOC detection.

Compared to other hydrogel-based VOC sensors that lack regenerable networks,^16,63^ these imine-cross-linked PDA/Ln-HAp@L hydrogels offer the promise for VOC sensing with their self-healing ability to regenerate and be reused in dynamic settings as well as their injectability enables them to conform to various environments. Overall, the appropriate luminescence and excellent dynamic features (i.e., self-healing and injectability) of the PDA/Ln-HAp@L hydrogels make them promising candidates for advanced VOC sensing applications.

## Conclusions

Our study focuses on the design principles that utilize HAp as a versatile platform for lanthanide–ligand pairs. The amine-terminated Ln-HAp@L can effectively engage in spontaneous cross-linking of aldehyde-functionalized PDA through imine bonds, facilitating the formation of nanocomposite hydrogels through dynamic covalent chemistry. Specifically, the lanthanide–ligand pairs attached to the HAp surface can be customized by selecting different lanthanides and ligands, which enables the generation of varying binding affinities and luminescence characteristics of the coordination pairs. This flexibility allows for precisely modulating the structures and properties of the resulting PDA/HAp nanocomposite hydrogels. In particular, the PDA/Ln-HAp@BP hydrogels were relatively unstable compared with the PDA/Ln-HAp@NTA and PDA/Ln-HAp@DPA hydrogels. Among the stable six hydrogel systems, the PDA/Eu-HAp@NTA hydrogel demonstrated the longest gelation time of ∼88 min, the lowest cross-link density of 0.51 mol/m^3^, and the smallest compressive modulus of ∼14 kPa. In contrast, the PDA/Dy-HAp@NTA hydrogels exhibited the shortest gelation time of ∼19 min, the highest cross-link density of 3.46 mol/m^3^, and the largest compressive modulus of ∼24 kPa. Our findings indicate that the Dy-NTA pair on the HAp surface, with the small size of Dy and the multidentate feature of NTA, achieves enhanced luminescence and improved cross-linking efficiency within the hydrogel network. This research concludes the importance of strategically selecting both the metal–ligand pairs as well as the interfacial cross-linking chemistry between nanomaterial cross-linkers and polymers at the molecular level to optimize the performance of luminescent nanocomposite hydrogels.

Taken together, the established relationship between metal–ligand pairs and features of nanocomposite hydrogels can serve as guidance for further exploration of luminescent materials and can be potentially used by other researchers to predict the optimum. The PDA/Eu-HAp@NTA lyophilized hydrogels were also demonstrated in differentiating VOCs under LDA. This versatile luminescent PDA/Ln-HAp@L nanocomposite hydrogel system potentially enhances the utility of lanthanide-based luminescent materials across diverse applications by tuning their structural, optical, and mechanical properties to match specific functional requirements, driving innovative advancements in materials science and biomedicine.

## Materials and Methods

### Materials

Hydroxyapatite within the size range of 50–70 nm was purchased from Sigma-Aldrich. Europium(III) nitrate hexahydrate (Eu(NO_3_)_3_·6H_2_O, 99%), terbium(III) nitrate pentahydrate (Tb(NO_3_)_3_·5H_2_O, 99%), and dysprosium(III) nitrate hydrate (Dy(NO_3_)_3_·*x*H_2_O, >99%) were supplied by Morrchem. 2,2′-((5-Amino-1-carboxypentyl)azanediyl)diacetic acid (NTA), carbon disulfide, and pamidronate disodium salt hydrate (BP) were purchased from BLD Pharmatech. 2-(Bromomethyl)pyridine hydrobromide and ninhydrin (99%) were purchased from Alfa Aesar. *N*-Boc-1,6-diaminohexane hydrochloride, *N*-Boc-ethylenediamine hydrochloride, sodium carbonate (Na_2_CO_3_), and potassium carbonate (K_2_CO_3_) were purchased from Fisher Scientific. Trifluoroacetic acid was purchased from Fluorochem. Polydextran (Mw ca. 500 kDa), sodium metaperiodate (NaIO_4_, 98%), diethylene glycol (99%), hydroxylamine hydrochloride (99%), chloroform (CHCl_3_), ethyl acetate, methanol, ethanol, toluene, and dichloromethane (DCM) were purchased from Duksan.

### Characterization Techniques

The ^1^H nuclear magnetic resonance (NMR) spectrum of PDA was measured using a Bruker AVIII HD 400 NMR (samples were dissolved in D_2_O), and the Fourier transform infrared (FTIR) spectra of PDA were obtained from a PerkinElmer Spectrum Two. GPC measurements were performed on an Enshine SUPER CO-150 system equipped with an RI-2031 detector by using HPLC-grade water as the eluent. HPLC Column TSKgel G5000PW 1000 Å as a chromatographic column, and the calibration curve used to calculate *M*_n_ was manufactured from 5,000 to 800,000 standards. The GPC results were calculated using the Chromatography software W3100. Dynamic light scattering (DLS) analysis was recorded on 90Plus (Brookhaven). ζ-Potential was measured by a Malvern Zetasizer Nano with a 633 nm wavelength laser. The FTIR spectra were obtained from a PerkinElmer Spectrum Two. The SEM was carried out on a TM-3000 (Hitachi). An accelerating voltage of 15 kV was used for SEM. The SEM images were analyzed using ImageJ software, and the average particle size was determined based on 30 different particles, with a rod shape. X-ray photoelectron spectroscopy (XPS) was analyzed using the ULVAC PHI 5000 Versa Probe. Mechanical properties were tested using a Materials Testing System (AGS-X-table type, Shimadzu) equipped with a 10 N load cell. The rheological characteristics of hydrogels were measured by using a rheometer (AR 2000EX, TA Instruments) equipped with a 20 mm parallel plate. In the oscillation time sweep, the strain was set at 1.0%, angular frequency 10 rad/s at 25 °C.

### Syntheses and Characterizations of *N*′,*N*′-Bis(pyridin-2-ylmethyl)hexane-1,6-diamine (DPA) and *N*′,*N*′-Bis(pyridin-2-ylmethyl)ethane-1,2-diamine (sDPA)

*N*-Boc-1,6-diaminohexane hydrochloride and N-boc-ethylenediamine hydrochloride were used to synthesize DPA and sDPA, respectively. *N*-Boc-1,6-diaminohexane hydrochloride (2.29 mL, 9.6 mmol) (or N-boc-ethylenediamine hydrochloride (1.87 mL, 9.6 mmol)) was slowly added to a stirring solution of 2-(bromomethyl)pyridine hydrobromide (5.0 g, 19.8 mmol) and Na_2_CO_3_ (4.46 g, 42.1 mmol) in methanol (50 mL) at room temperature. The mixture was stirred continuously for 24 h. After completion, the reaction mixture was filtered to separate the solid byproducts, and the methanol in the filtrate was removed under reduced pressure. The remaining material was redissolved in dichloromethane and subjected to column chromatography with ethyl acetate/*n*-hexane (1:1) as the eluent, yielding the product as a yellow oil. This product (2.0 g) was dissolved in dichloromethane (20 mL), and trifluoroacetic acid (TFA, 5 mL, 65 mmol) was added at 0 °C. The reaction mixture was stirred overnight at room temperature. The solvent was then evaporated under reduced pressure, and the resulting crude product was washed with ether to obtain the target product.

^1^H NMR spectrum of DPA (600 MHz, D_2_O): δ 8.59 (d, 2H), 8.42–8.36 (m, 2H), 7.92 (d, 2H), 7.85–7.79 (m, 2H), 4.16 (s, 4H), 2.92–2.69 (m, 2H), 2.65–2.45 (m, 2H), 1.52–1.24 (m, 4H), 1.19–0.83 (m, 4H) (Figure S35a). The electrospray ionization (ESI) mass spectrum of DPA is shown in Figure S35b. ^1^H NMR spectrum of sDPA (600 MHz, D_2_O): δ 8.59 (d, 2H), 8.44–8.37 (m, 2H), 7.92 (d, 2H), 7.87–7.80 (m, 2H), 4.14 (s, 4H), 3.24–3.08 (m, 2H), 2.98–2.85 (m, 2H) (Figure S36a). ESI mass spectrum of sDPA is shown in Figure S36b.

### Synthesis and Characterization of Polydextran Aldehyde

Polydextran (10 g, 0.02 mmol) was first dissolved in deionized water (250 mL). Sodium periodate (4.75 g, 22.21 mmol) was added to the dextran solution, and the mixture was stirred in the dark at room temperature for 24 h for the polydextran oxidation. The reaction was quenched by adding diethylene glycol (30 mL, 316.62 mmol) and stirring for 30 min. The solution was then dialyzed against Milli-Q water for 3 days using a membrane with a 12–14 kDa MWCO. Finally, the product was freeze-dried at −80 °C and 10 mTorr, resulting in polydextran aldehyde (PDA) with an oxidation degree of approximately 27.2%, as determined by hydroxylamine hydrochloride.^25^ The molecular weight of PDA was 178 300 g/mol (PDI = 1.20) determined by gel permeation chromatography (GPC).

### Synthesis of Functionalized Lanthanide-Containing Hydroxyapatite

Lanthanide-containing hydroxyapatite (Ln-HAp@L) was prepared using a two-step process.^64^ First, hydroxyapatite (0.5 g) with a particle size of 60 ± 10 nm and lanthanide nitrate (Ln(NO_3_)_3_) (0.5 mmol) were refluxed in deionized water (20 mL) for 24 h, followed by cooling and separation of the precipitate via centrifugation. Next, the obtained Ln-HAp was mixed with a ligand—*N*,*N*-bis(carboxymethyl)-l-lysine (NTA), *N*′,*N*′-bis(pyridin-2-ylmethyl)hexane-1,6-diamine (DPA), and sodium (3-amino-1-hydroxy-1-phosphonopropyl)phosphonate (BP) (0.5 mmol each)—in deionized water (20 mL) and heated at 80 °C for 24 h. After cooling, the product was again centrifuged to collect the precipitate, which was then lyophilized in a freeze-dryer (UNISS FDM-2).

### Preparation of Nanocomposite Hydrogels

Ln-HAp@L and PDA were separately dispersed in distilled water at concentrations of 20 and 14 wt %, respectively. The solutions of Ln-HAp@L and PDA were subsequently combined in equal volumes (200 μL). The mixture was then vigorously stirred to ensure a homogeneous dispersion of the hydrogels. Subsequently, the resulting mixture was left undisturbed for 24 h to ensure proper gel formation before further use.

### Swelling and Degradation Behaviors of Nanocomposite Hydrogels

The hydrogels were prepared freshly, and their weights (w_1_) were recorded. Subsequently, the hydrogels were immersed in DI water. To ensure consistent swelling, the hydrogels were placed in a precisely controlled incubator set at a temperature of 37 °C and a shaking speed of 100 rpm. After treatment, the hydrogels were carefully extracted from DI water. The excess liquid on the hydrogel surface was carefully wiped away to ensure accurate measurements, and then it was weighed (w_2_). The swelling ratio of the hydrogels was calculated using the following equation.
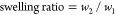

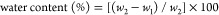


The degradability of hydrogels was determined by immersing the lyophilized hydrogels (∼19 mg, w_o_) in DI water. (1.0 mL), and then the hydrogels were lyophilized to measure mass (w_D_) after immersion.
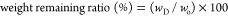


### Mechanical and Rheological Measurements of Nanocomposite Hydrogels

The compression speed was set to 1 mm/min, and the tests were conducted at room temperature. The force–displacement data during the loading response are recorded by stress–strain curves, and Young’s modulus is calculated as the slope of the stress–strain curve from 10 to 20%. The rheological properties of hydrogels were carried out by a rheometer (AR 2000EX, TA Instruments) with a 20 mm parallel plate. In the oscillation strain sweep test, the strain was swept from 0.1 to 1000% strain for 1 Hz at 25 °C with 10 points per decade. In the continuous flow sweep test, the shear rate was set between 0.1 and 100 s^–1^ for 1 Hz at 25 °C with 10 points per decade, illustrating the shear-thinning property. The self-healing property was demonstrated by the cyclic strain time sweep test under the low strain of 1.0% and high strain of 500% alternatively.

The force required to inject the hydrogels was measured by using a materials testing system equipped with a 10 N load cell. For this experiment, 500 μL of each hydrogel sample was filled into a 1 mL syringe (diameter 4.78 mm, BD). An 18G needle was then attached to the syringe, which was positioned over a Petri dish. The load cell moved downward until it came into contact with the syringe plunger. A compressive force was then applied at a traverse speed of 1.0 mm/min until the entire volume of the hydrogel was expelled from the syringe. During the process, a force–displacement curve was generated to analyze the dynamics of the injection force.

### Thermal Properties of Nanocomposite Hydrogels

Differential scanning calorimetry (DSC) was performed with a TA Instruments Q-20 instrument to evaluate the thermal properties of the hydrogels. The analysis was performed at a heating rate of 10 °C/min in a temperature range from −30 to 200 °C under a nitrogen flow of 50 mL/min.

### Microstructures of Nanocomposite Hydrogels

The hydrogels were prepared and subjected to lyophilization using a freeze-dryer (UNISS FDM-2) under conditions of −80 °C and 10 mTorr. The resulting lyophilized hydrogel samples were then examined for their microstructure using a Hitachi TM-3000 tabletop scanning electron microscope (SEM). The pore size analysis of the freeze-dried hydrogel was measured by a mercury porosimeter (Micromeritics AutoPore IV 9520). The experiments were performed at low pressures (345 kPa) and high pressures (414 MPa). This method was used to obtain information about pores larger than 3.6 nm. Micro-CT was employed to investigate the internal porous structure of the hydrogels. A 200 μL sample of fully lyophilized hydrogel was subjected to X-ray micro-CT imaging (Skyscan 1076). This technique allowed for a detailed, slice-by-slice analysis of the hydrogel’s microstructure.

### Computational Methods

Density functional theory (DFT) was employed to study the binding free energies between the three lanthanide ions (Eu^3+^/Tb^3+^/Dy^3+^) and the three ligands (NTA/DPA/BP) forming nine model systems. For each of the nine model systems, a 1:1 ratio of the ligand and lanthanide ion was considered. A methyl group was used to replace the alkyl side chain of the ligands without significantly affecting the complexation core. A dihydrogen phosphate (H_2_PO_4_^–^) molecule was chosen to represent the HAp surface environment, where its two P=O bonds offer the chelating ability with a coordination number of 2 in our model systems. To achieve the desired coordination environments for the central lanthanide ion (i.e., the coordination number of 8), varying numbers of water molecules (2/3/4) were used along with NTA/DPA/BP ligands, meanwhile capturing the first solvation shell of the complex in the hydrogels. Due to the high number of coordinating sites and ligands, numerous possible binding geometries exist. To efficiently explore these geometries, the Architector package, a new 3D structure generator for metal–ligand complexes developed by Taylor et al.^65^ was employed alongside our chemical intuition. All the generated complex structures (Ln-HAp@[L+H_2_O]), the complexes without NTA/DPA/BP ligands (Ln-HAp@H_2_O), and the NTA/DPA/BP ligands (L) alone were then optimized at PBE0-D3/6-31+G(d,p) level of theory,^66^ and the large-core MWB effective core potential (ECP) was incorporated for the lanthanide ions (MWB52 for Eu^3+^, MWB54 for Tb^3+^, and MWB55 for Dy^3+^).^67,68^ The lowest-energy structure identified for each model was used to represent the corresponding Ln-HAp@L system for a further investigation of its Ln···L interaction. Vibrational frequency calculations were performed to ensure the structures were at their equilibrium geometry and to obtain zero-point energies (ZPE) and thermodynamic information. Single-point energy calculations of the lowest-energy structures were then performed at the PCM/PBE0-D3/6-311++G(d,p)/ECP level of theory for more accurate energy estimations.^66−69^ The calculations include a PCM model with the water dielectric constant to account for the solvation effects.^70^ The binding energy (*E*_b_) between the lanthanide ion and the ligand was calculated using the following equation:
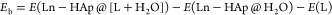


As mentioned above, the ZPE and thermal corrections were obtained from the gas phase frequency calculations and applied to the single-point energies to calculate the Gibbs binding free energies (Δ*G* in Table S8). All the DFT calculations were performed with the Gaussian 16 package.^71^

### VOC Sensing

PDA/Eu-HAp@NTA lyophilized hydrogel was placed in custom-made gas sensing molds to investigate their VOC sensing capabilities. Various VOCs (1 M, 1 mL) were volatilized for 2 h to create gaseous environments for testing. The luminescence spectra of the lyophilized hydrogel samples were measured by using an LSLS-QY instrument, and the data were further analyzed with linear discriminant analysis (LDA) using XLSTAT 2024 software.

### Statistical Analysis

All measurements were repeated three times, and the error bars in the figures represent the standard deviation. A *t*-test was used to analyze whether the differences among the data were statistically significant. Significance was set at *p* < 0.05 with *, **, or *** indicating *p* < 0.05, 0.01, or 0.001, respectively.

## Supporting Information Available

SEM images, SEM-EDS elemental mapping, photographs of hydrogel, CIE chromaticity coordinates of hydrogel, UV–visible spectra, ESI-MS spectra, hydrodynamic sizes, ζ-potentials, XRD patterns, FTIR spectra, XPS spectra, modulus of hydrogel, luminescence spectrum, compressive stress–strain curve, DSC, equilibrium swelling ratio of hydrogel, equilibrium water content of hydrogel, structure of Ln-H_2_PO_4_@L·*x*H_2_O, shear stress–shear rate curves of hydrogel, demonstrations of the injectability of hydrogel, demonstrations of inversion test of hydrogel, ^1^H NMR spectra, PLQY values of nanoparticle and hydrogel, Ln and amine amount of nanoparticle, MIP analyses of hydrogel, micro**-**CT analyses of hydrogel, rheological analyses of hydrogel, optimized structures (*xyz* coordinates) from DFT calculations, and Δ*G* of the Ln-H_2_PO_4_@L·*x*H_2_O complexes calculated from the computational simulation (PDF)
