# Supplementary material for: Tailoring Nanomaterial Cross-Linkers through Lanthanide–Ligand Pairs: Guidance for Fine-Tuning the Structures and Properties of Luminescent Nanocomposite Hydrogels
Source: Inorg Chem. 2025 Apr 24;64(17):8601–19. doi: 10.1021/acs.inorgchem.5c00130 (PMC12056695; doi:10.1021/acs.inorgchem.5c00130)
Supplement: Supplementary file 1 — ic5c00130_si_001.pdf [file ic5c00130_si_001.pdf]

Supporting information

Tailoring nanomaterial crosslinkers through lanthanide-ligand pairs: guidance for fine-tuning the structures and properties of luminescent nanocomposite hydrogels

Yu-Chia Su[a], Li Chu Tseng[b], Wei-Tao Peng[b,c], Chao-Ping Hsu[b,d], and Yi-Cheun Yeh[a*]

[a] Institute of Polymer Science and Engineering, National Taiwan University, Taipei 10617, Taiwan.

[b] Institute of Chemistry, Academia Sinica, Taipei 115201, Taiwan

[c] Department of Chemistry, Tunghai University, Taichung 40704, Taiwan

[d] National Center for Theoretical Sciences, Physics Division, Taipei 106319, Taiwan

* Corresponding author. E-mail address: yicheun@ntu.edu.tw (Y.-C. Yeh)

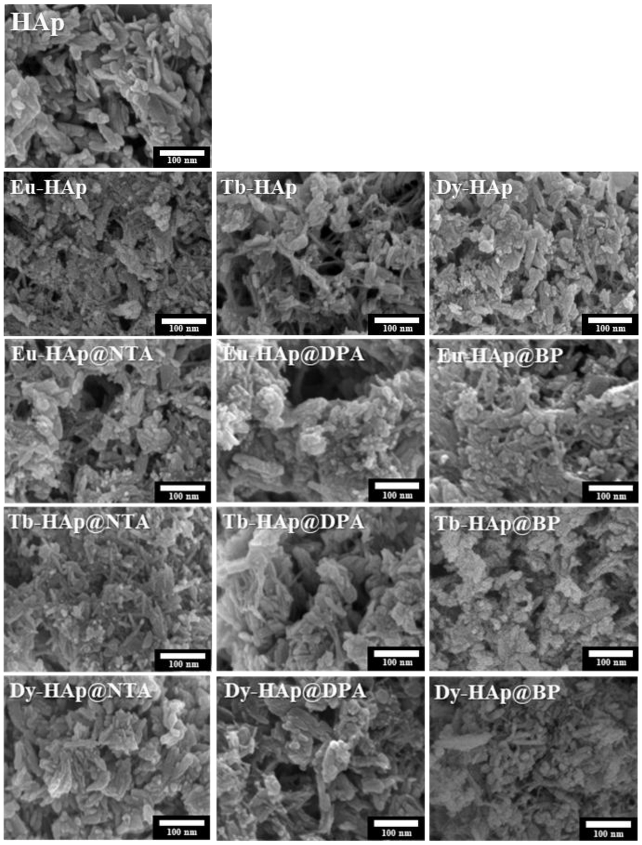

Figure S1. Representative SEM images of HAp, Ln-HAp, and Ln-HAp@L.

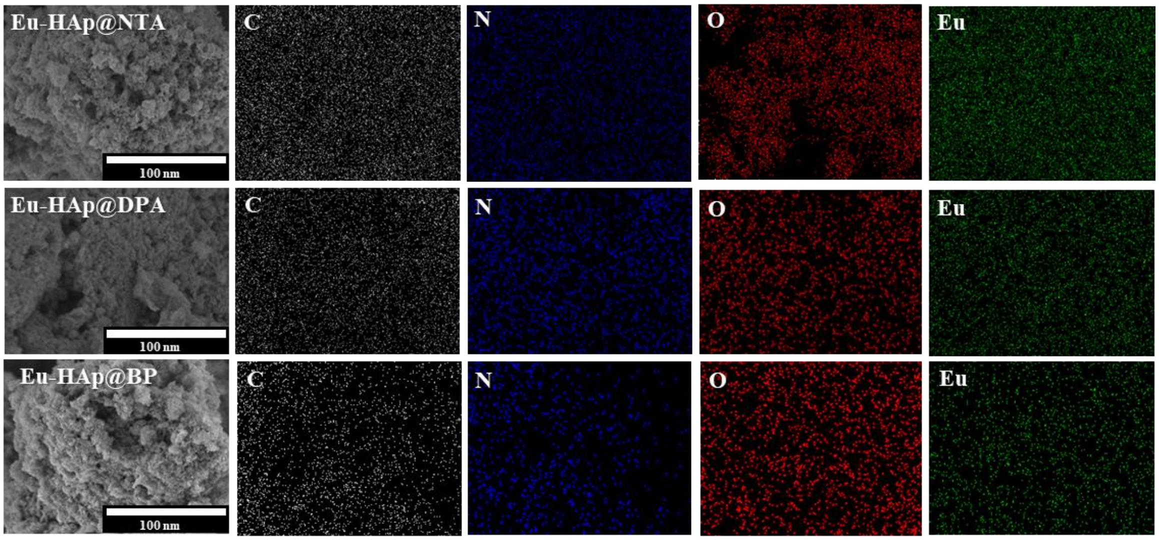

Figure S2. SEM-EDS elemental mapping of Eu-HAp@L.

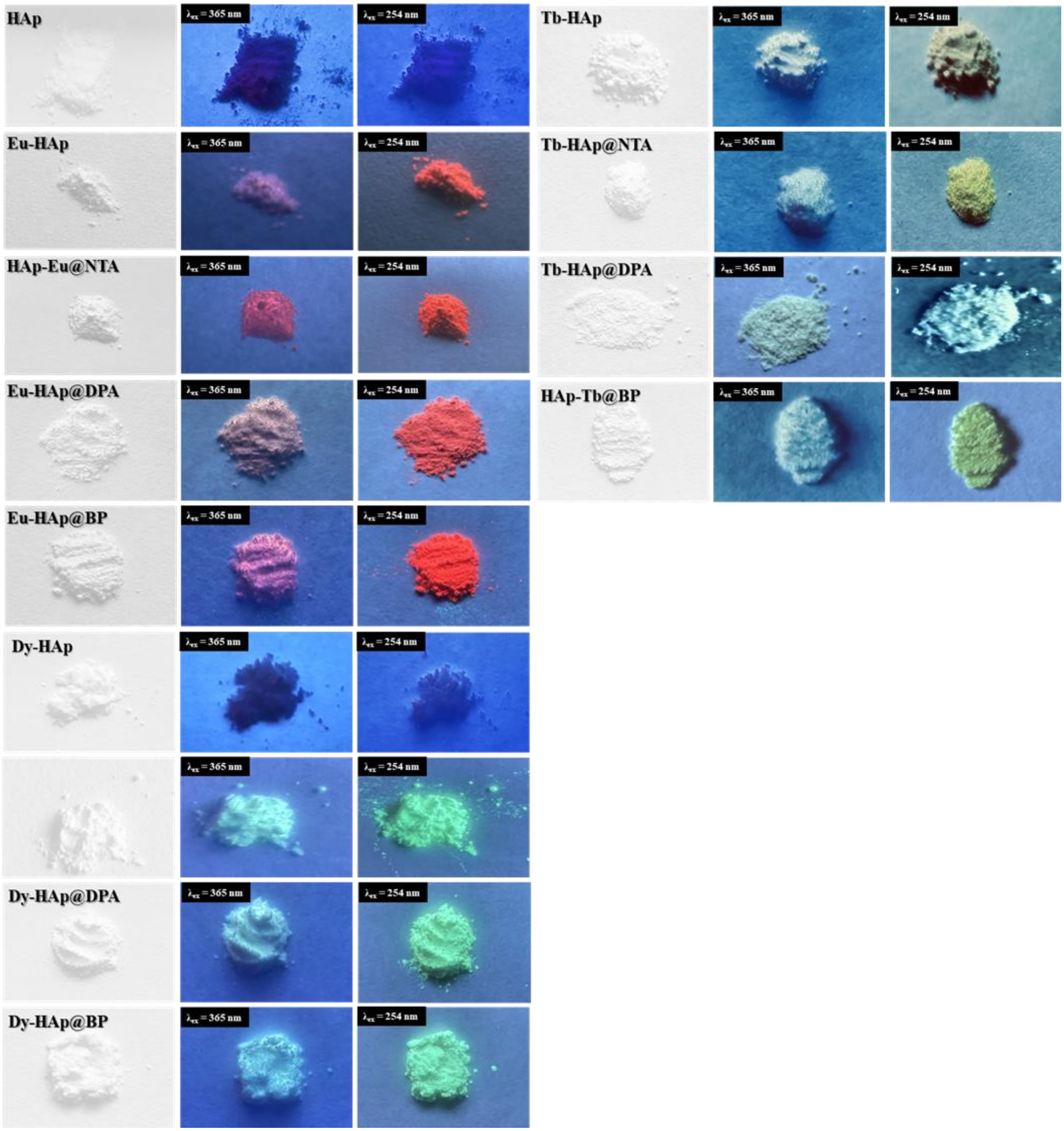

Figure S3. Photographs of Ln-HAp@L nanoparticles under UV excitations.

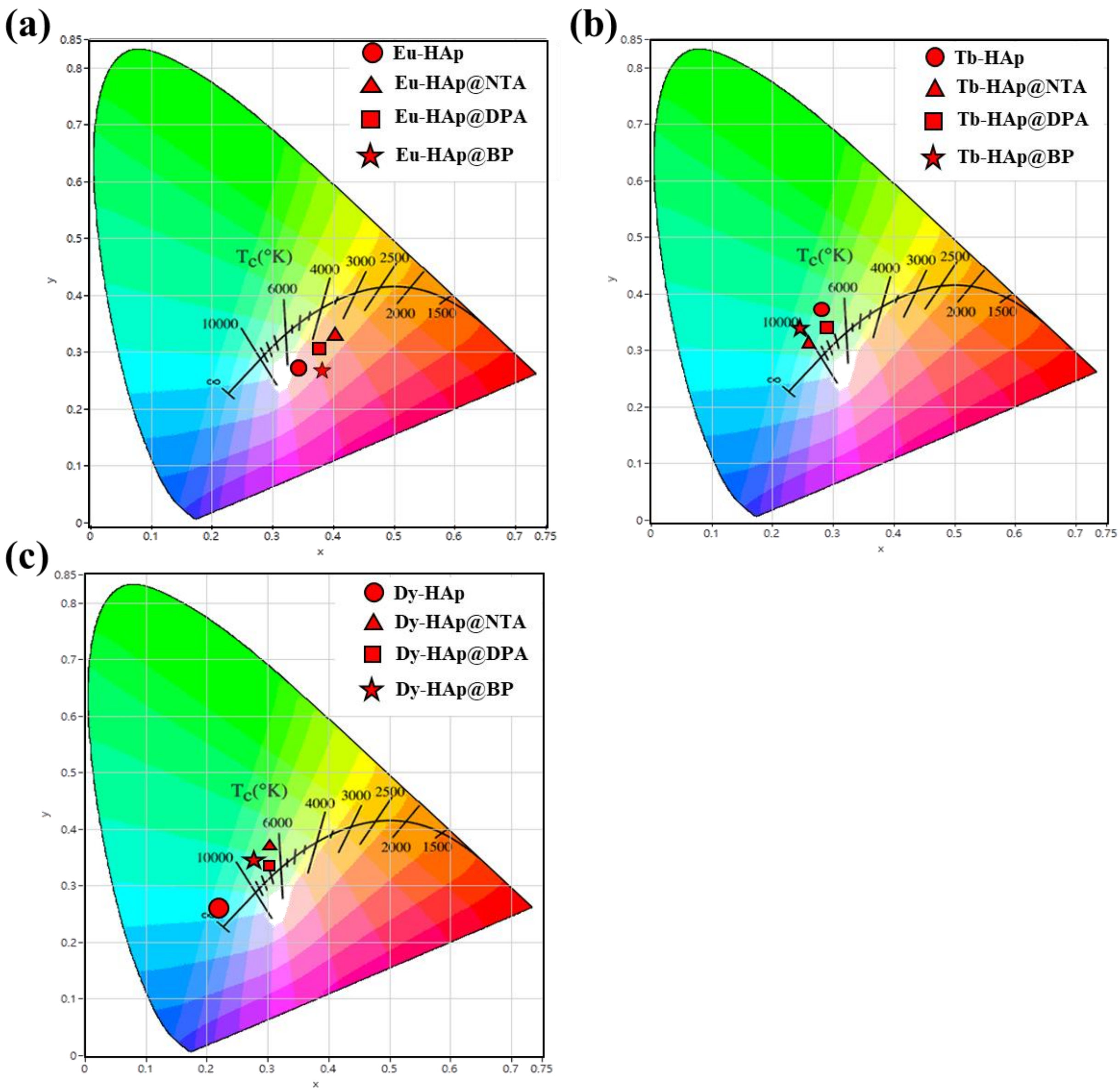

Figure S4. CIE chromaticity coordinates of Ln-HAp and Ln-HAp@L.

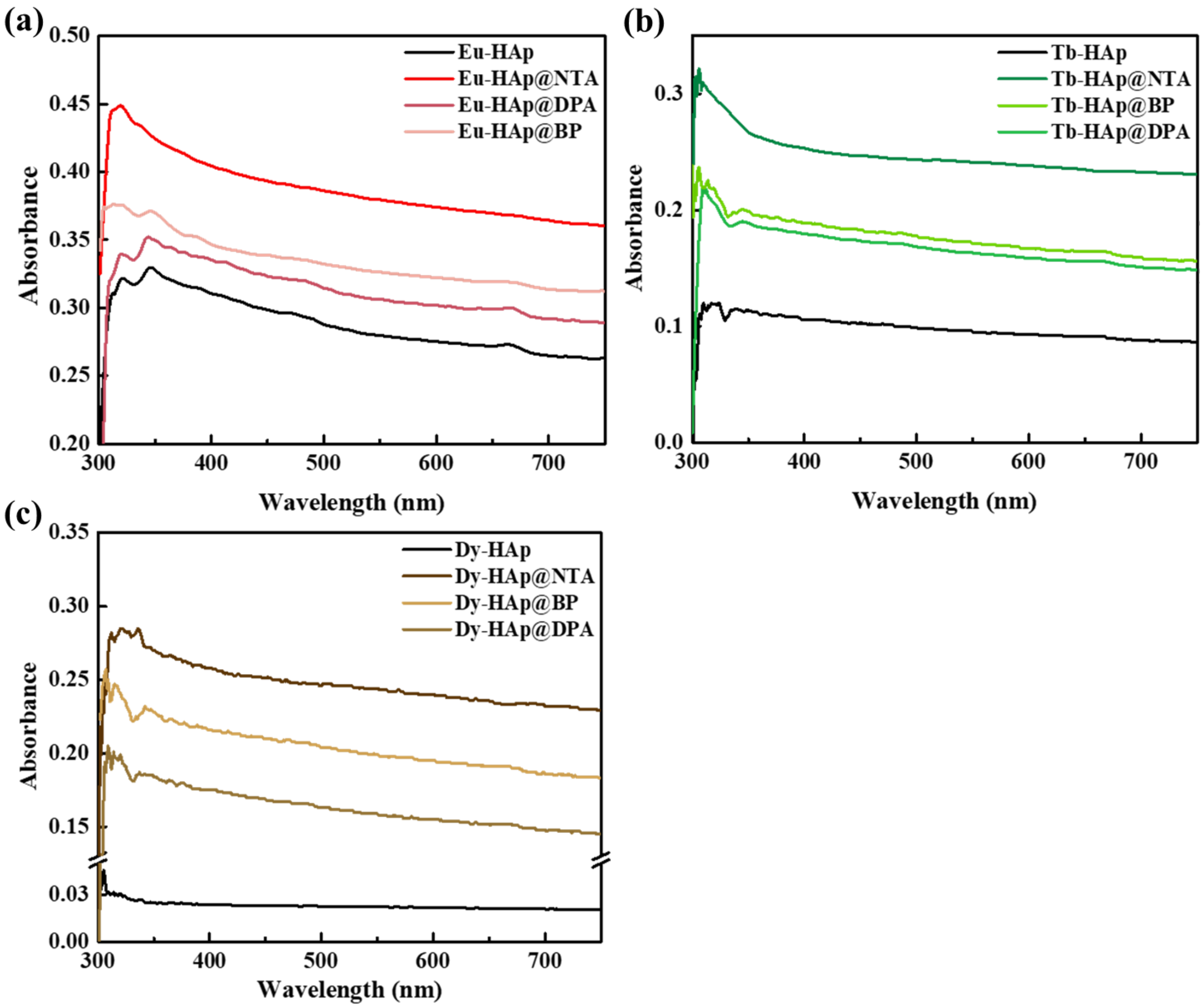

Figure S5. UV-visible spectra of Ln-HAp and Ln-HAp@L.

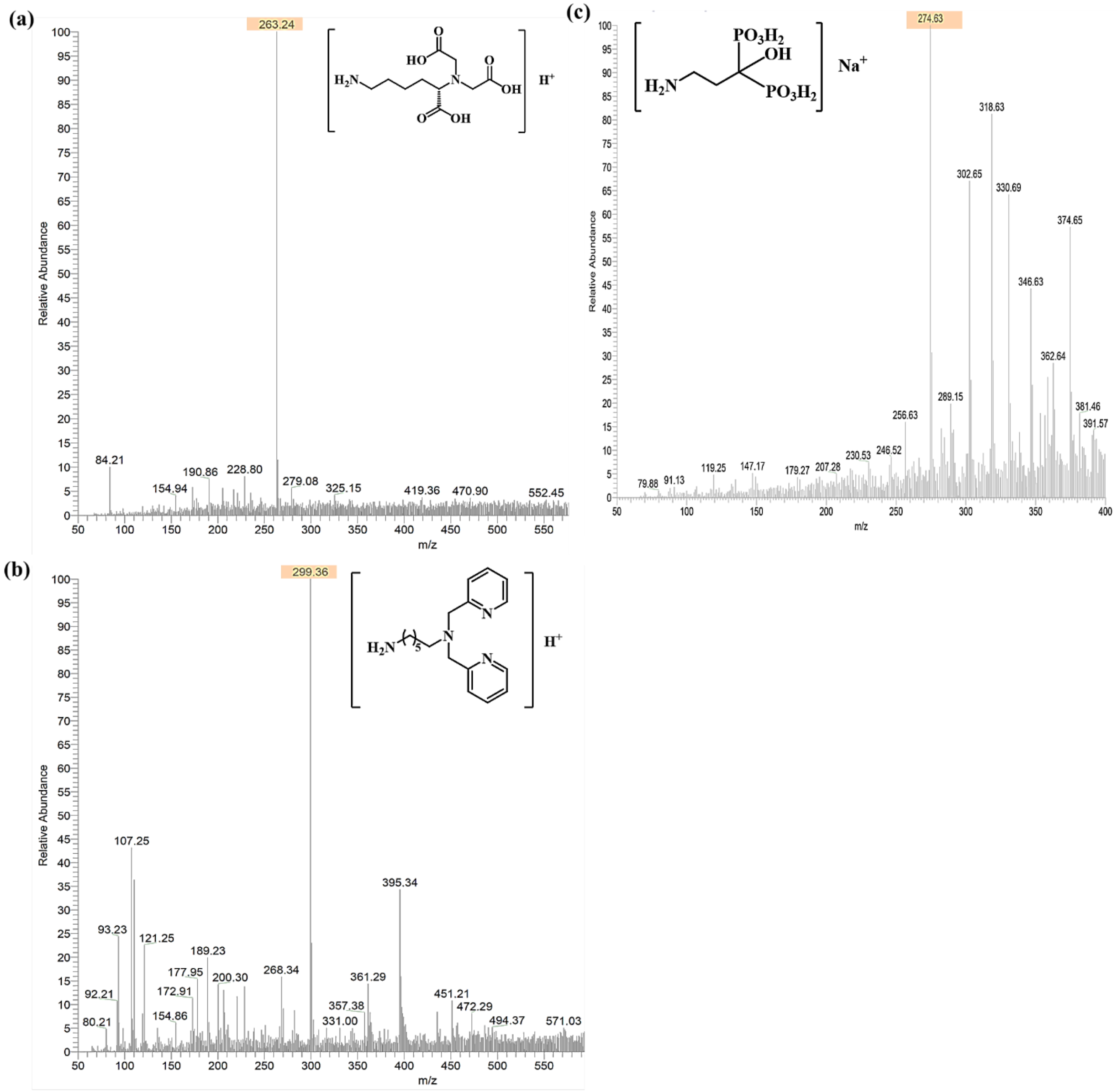

Figure S6. ESI-MS spectra of Eu-HAp@L after being immersed in the HCl solution.

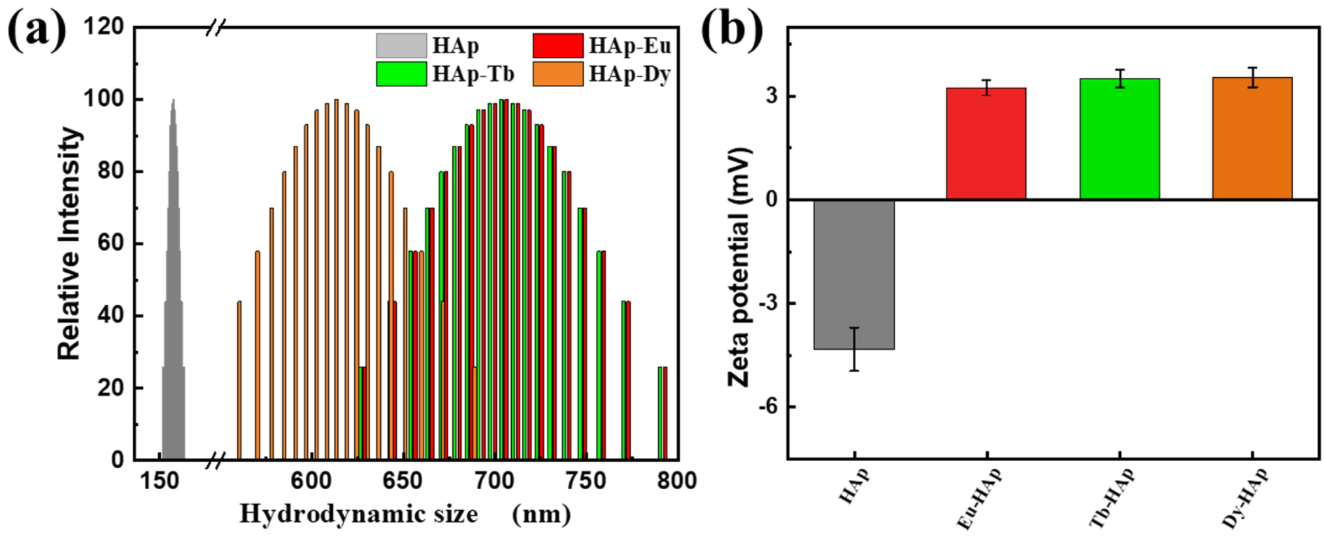

Figure S7. (a) Hydrodynamic sizes and (b) zeta potentials of HAp and Ln-HAp.

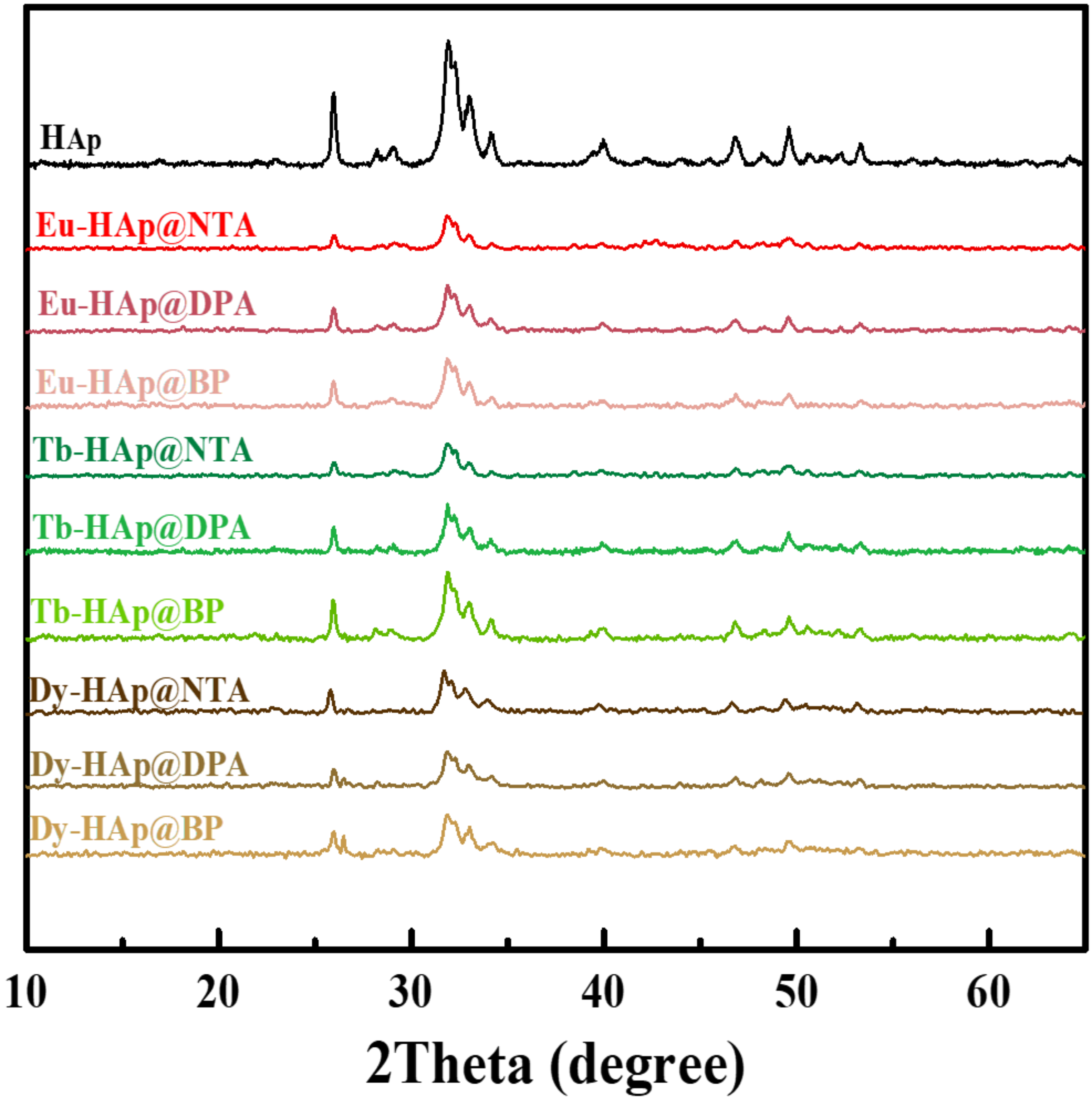

Figure S8. XRD patterns of HAp and Ln-HAp@L.

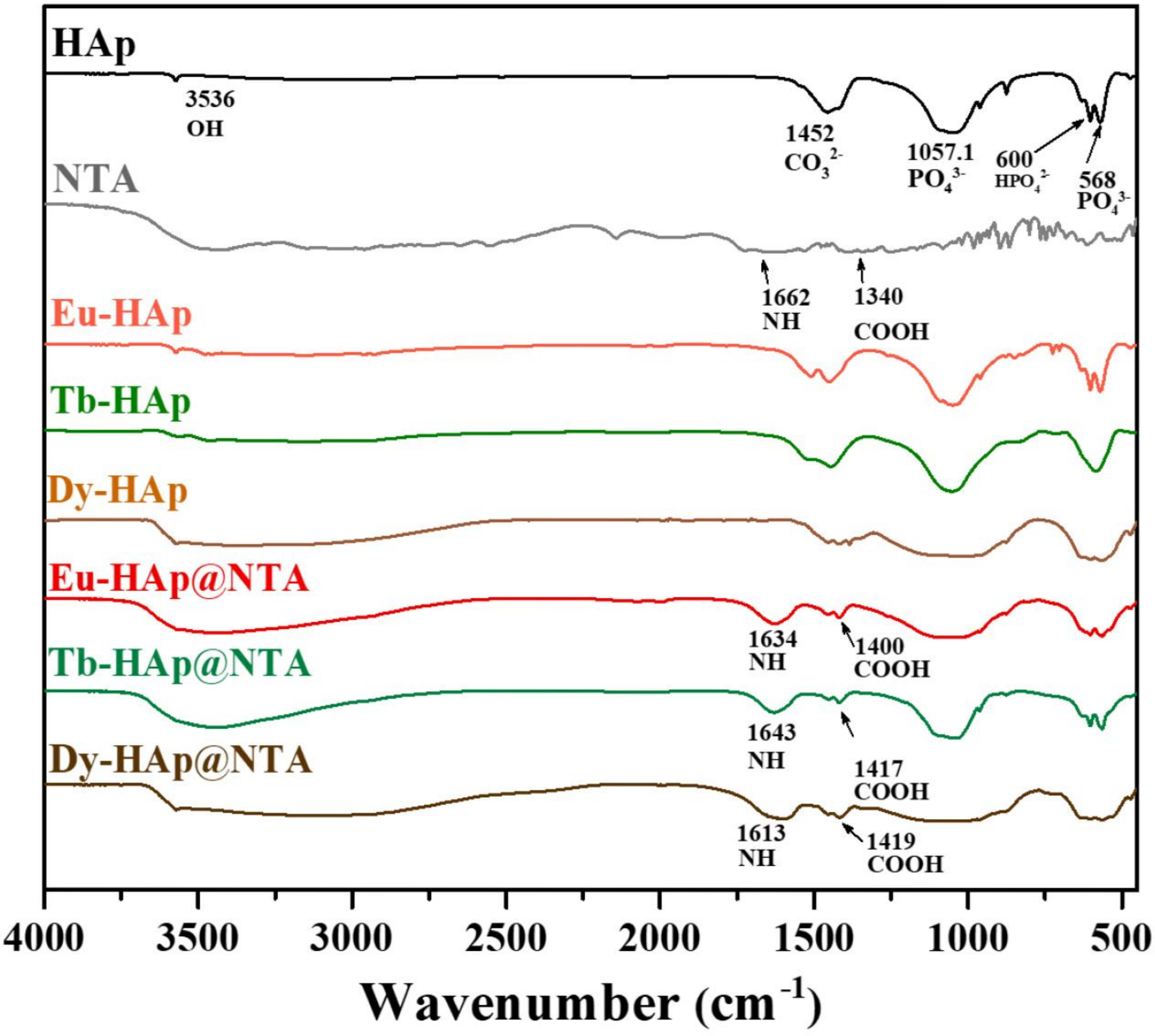

Figure S9. FTIR spectra of HAp, NTA, Ln-HAp, and Ln-HAp@NTA.

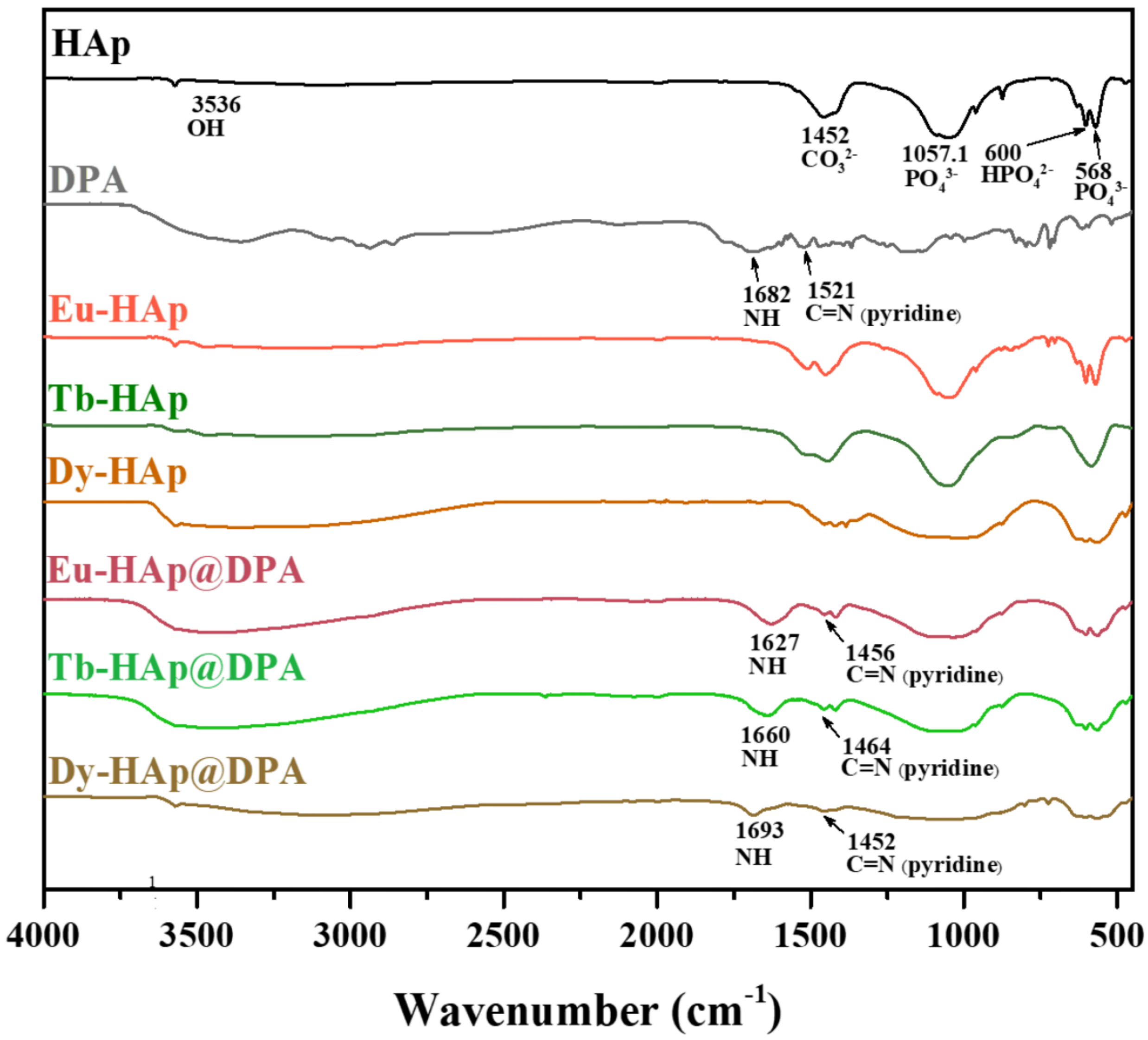

Figure S10. FTIR spectra of HAp, DPA, Ln-HAp, and Ln-HAp@DPA.

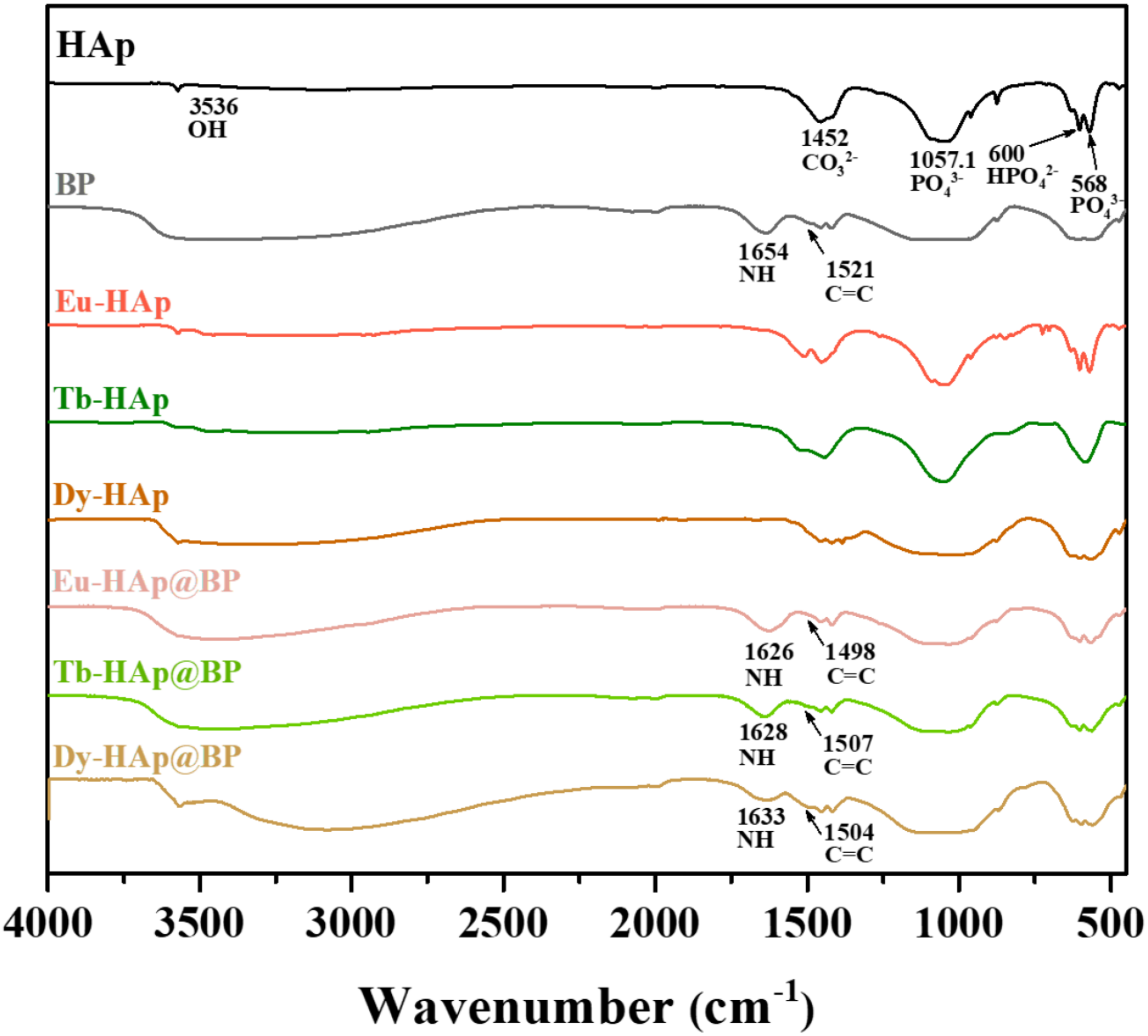

Figure S11. FTIR spectra of HAp, BP, Ln-HAp, and Ln-HAp@BP.

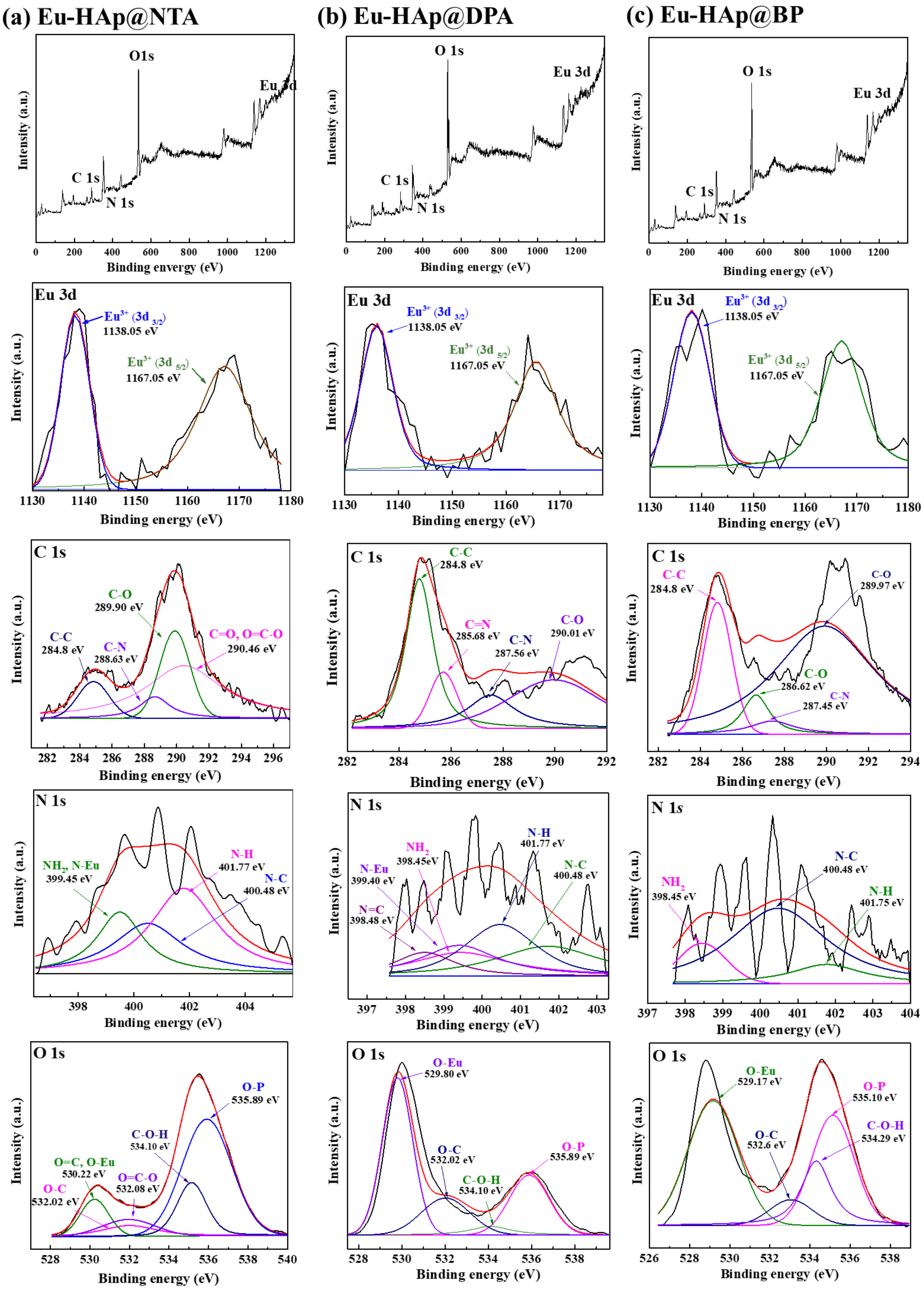

Figure S12. XPS spectra of (a) Eu-HAp@NTA, (b) Eu-HAp@DPA, and (c) Eu-HAp@BP.

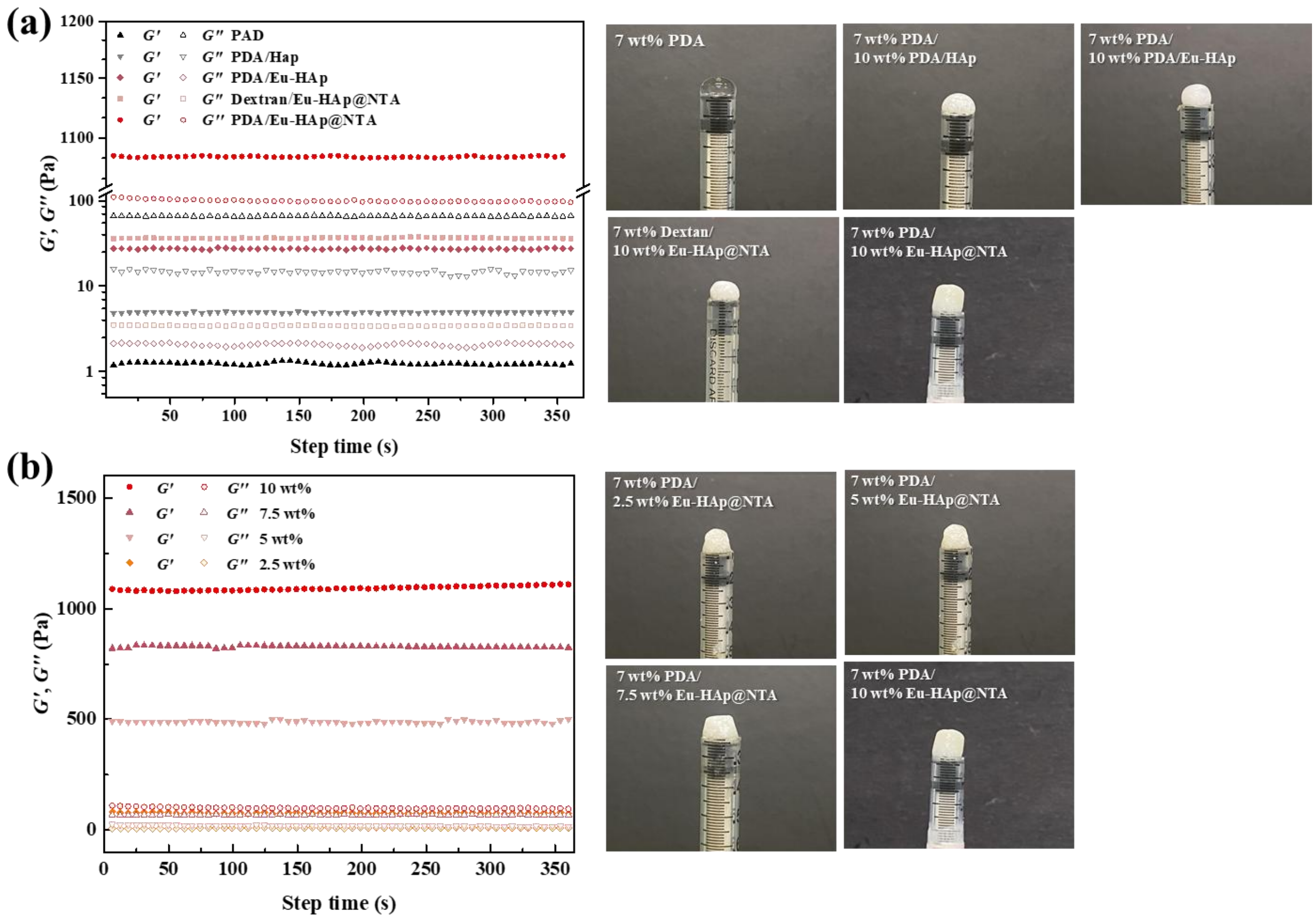

Figure S13. (a) Continuous time sweeps and photos of PDA, PDA/HAp, PDA/Eu-HAp, Dextran/Eu-HAp@NTA, and PDA/Eu-HAp@NTA samples. (b) Continuous time sweeps and photos of PDA/Eu-HAp@NTA hydrogels prepared with different wt% of Eu-HAp@NTA.

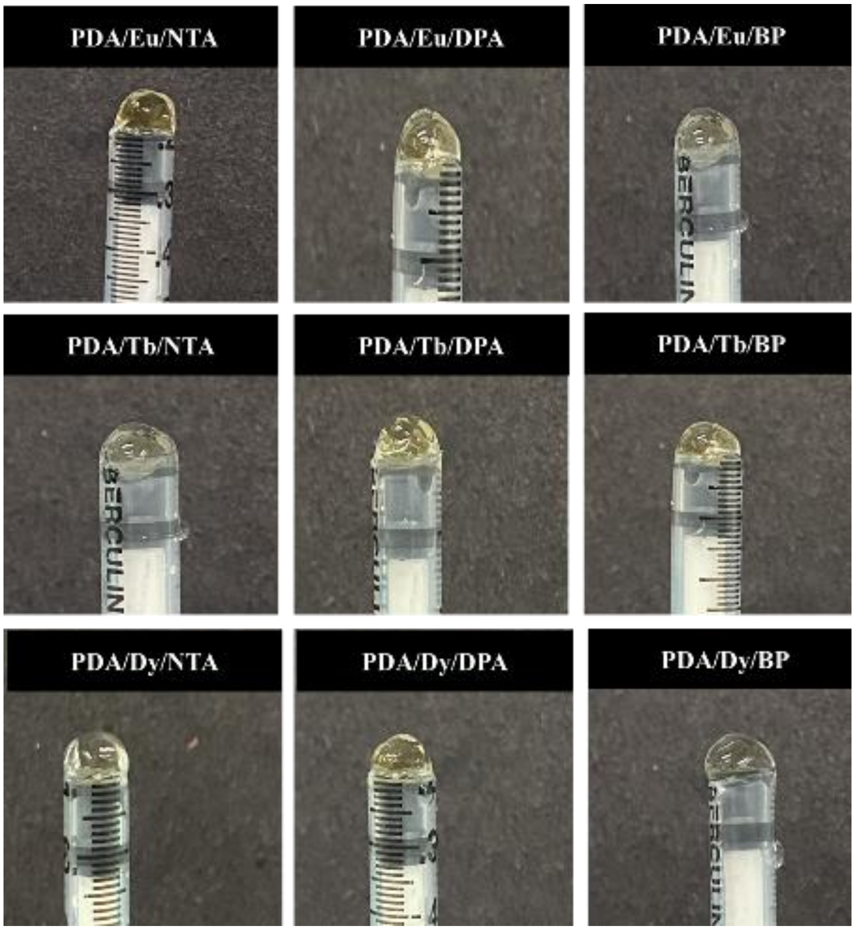

Fig S14 Photos of PDA/Ln/L mixtures of PDA (7 wt%), $Ln(NO_3)_3 \cdot 6H_2O$ (5wt%), and amine-terminated ligand (5wt%).

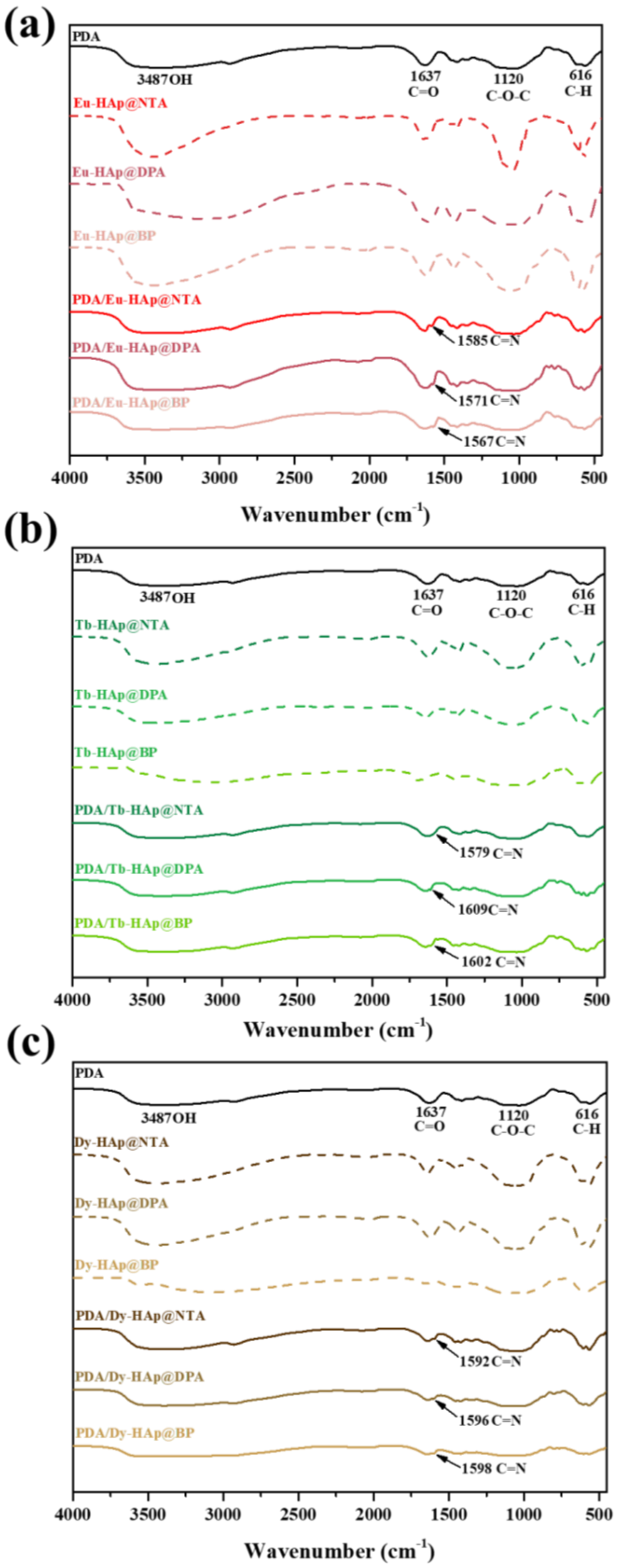

Figure S15. FTIR spectra of PDA, Ln-HAp@L, and PDA/Ln-HAp@L.

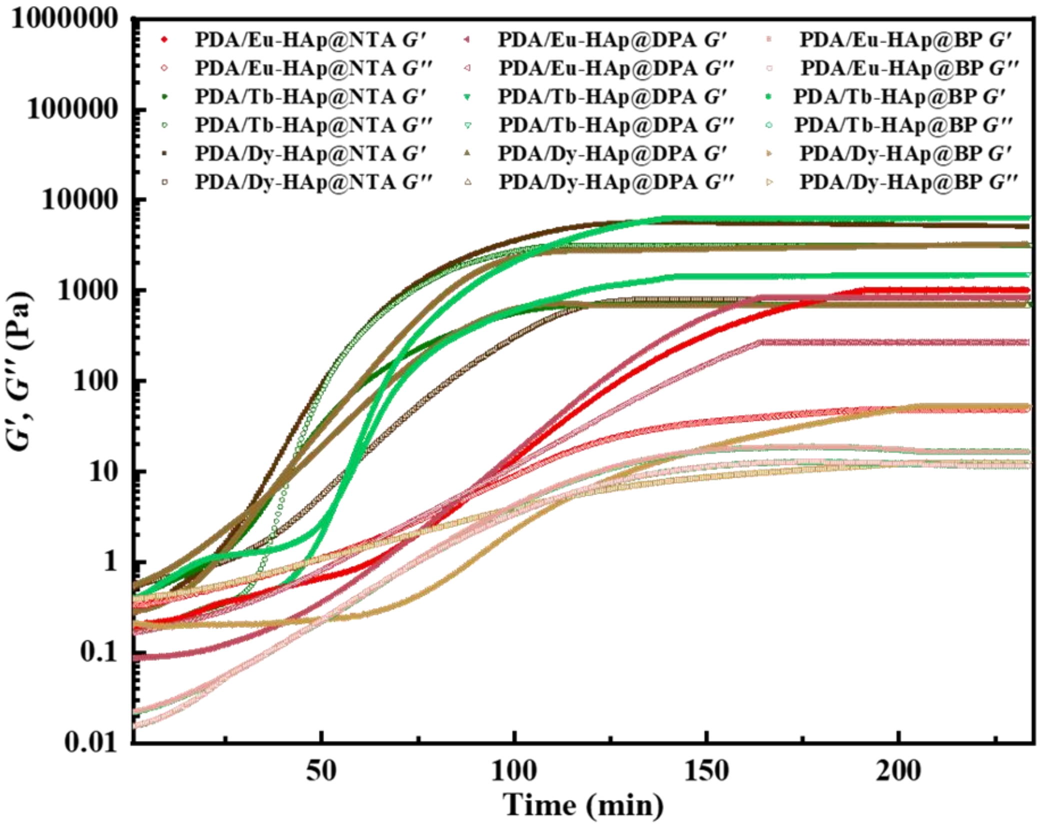

Figure S16. Continuous-time sweeps of PDA/Ln-HAp@L hydrogels.

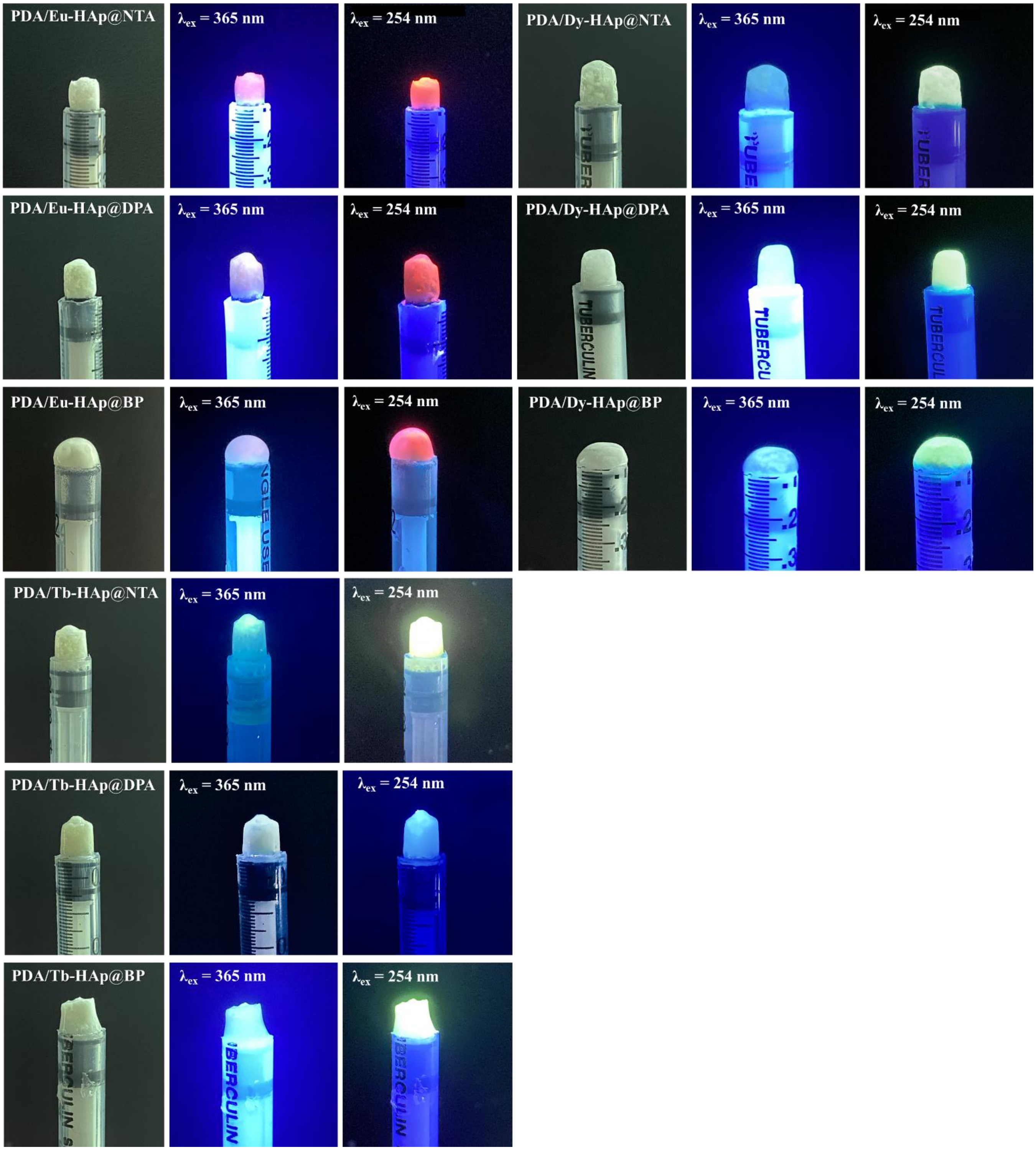

Figure S17. Photographs of PDA/Ln-HAp@L hydrogels under UV excitations.

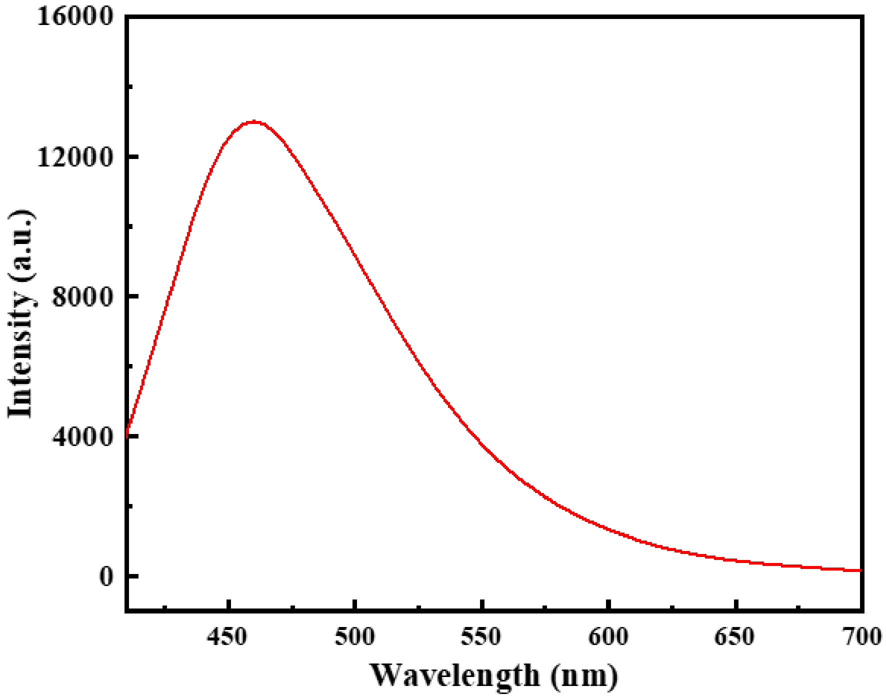

Figure S18. Luminescence spectrum of the PDA hydrogel, where PDA was crosslinked with ethylenediamine.

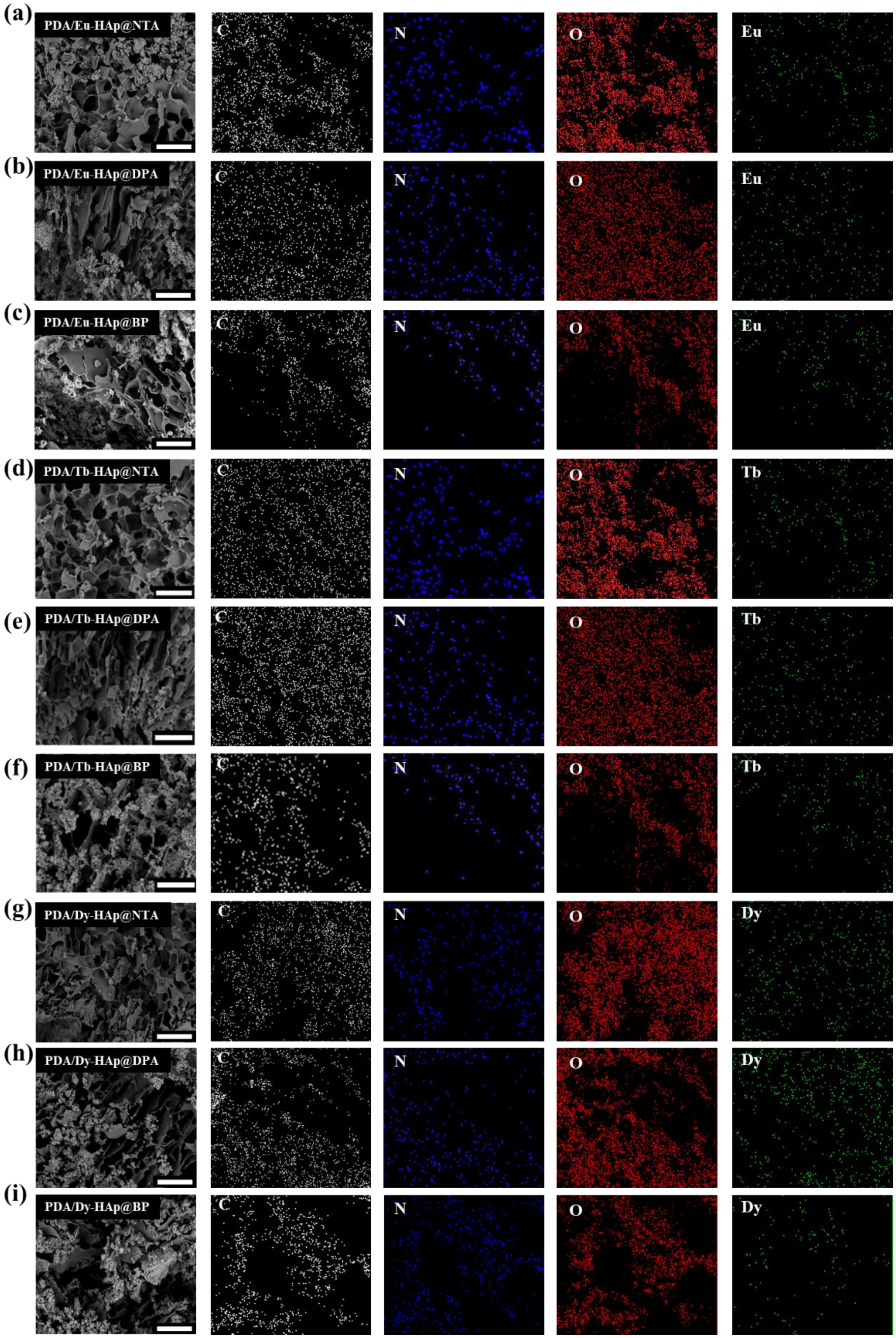

Figure S19. SEM-EDS elemental mapping of PDA/Ln-HAp@L hydrogels. Scale bar= 100 µm.

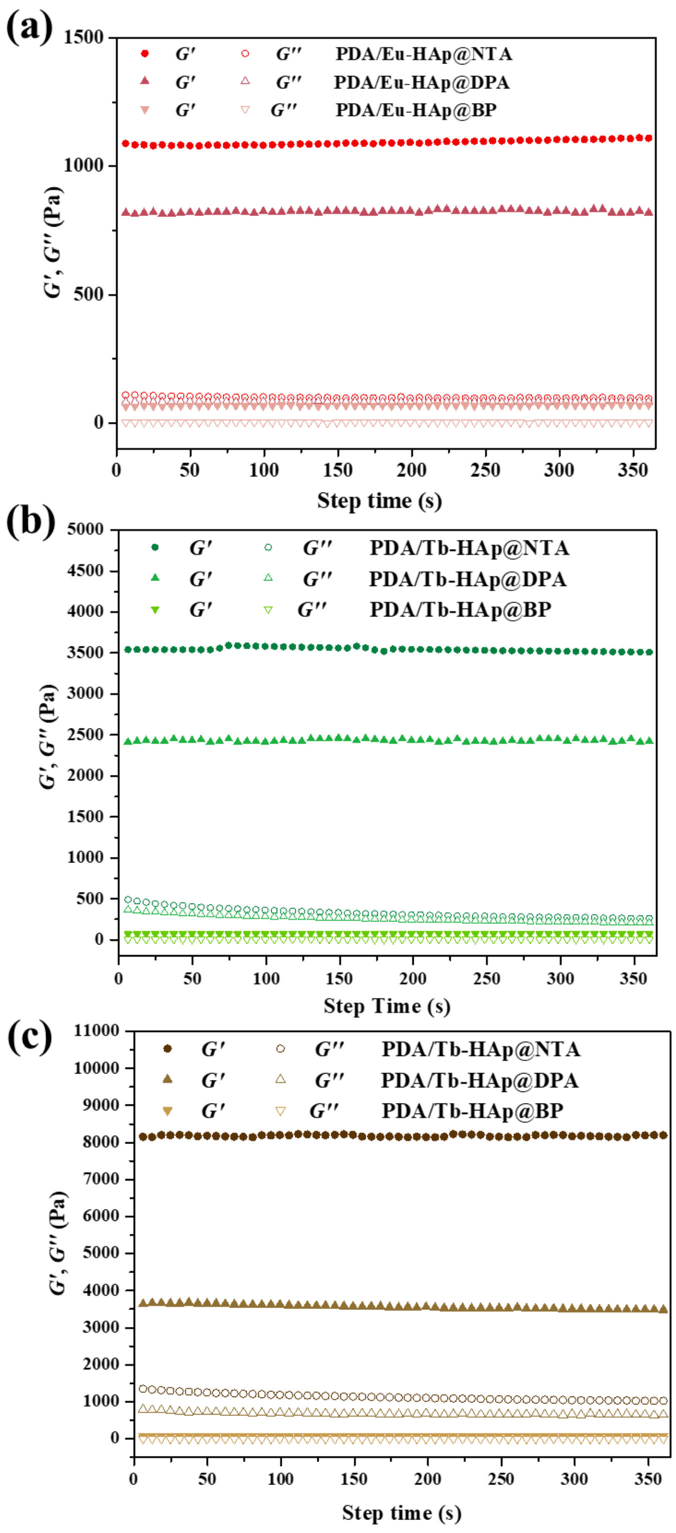

Figure S20. Oscillatory time sweeps of (a) PDA/Eu-HAp@L, (b) PDA/Tb-HAp@L, and (c) PDA/Dy-HAp@L hydrogels.

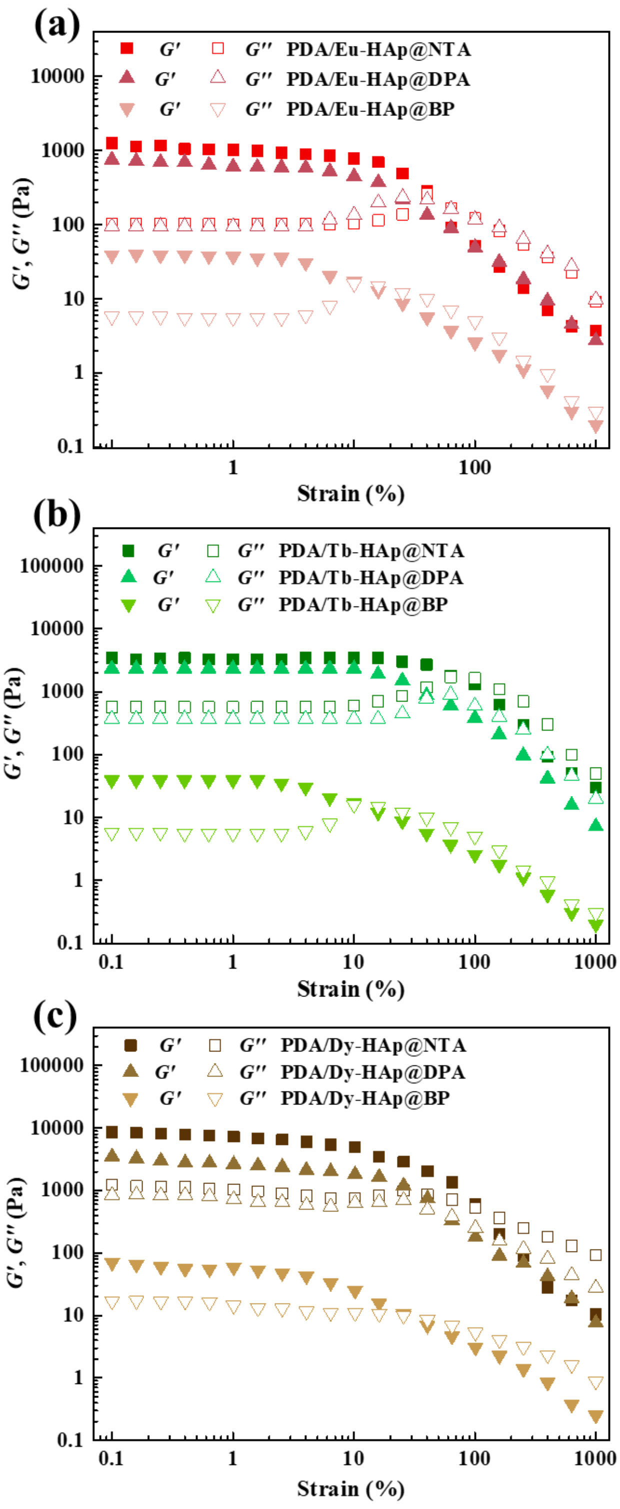

Figure S21. Oscillation strain sweeps of (a) PDA/Eu-HAp@L, (b) PDA/Tb-HAp@L, and (c) PDA/Dy-HAp@L hydrogels.

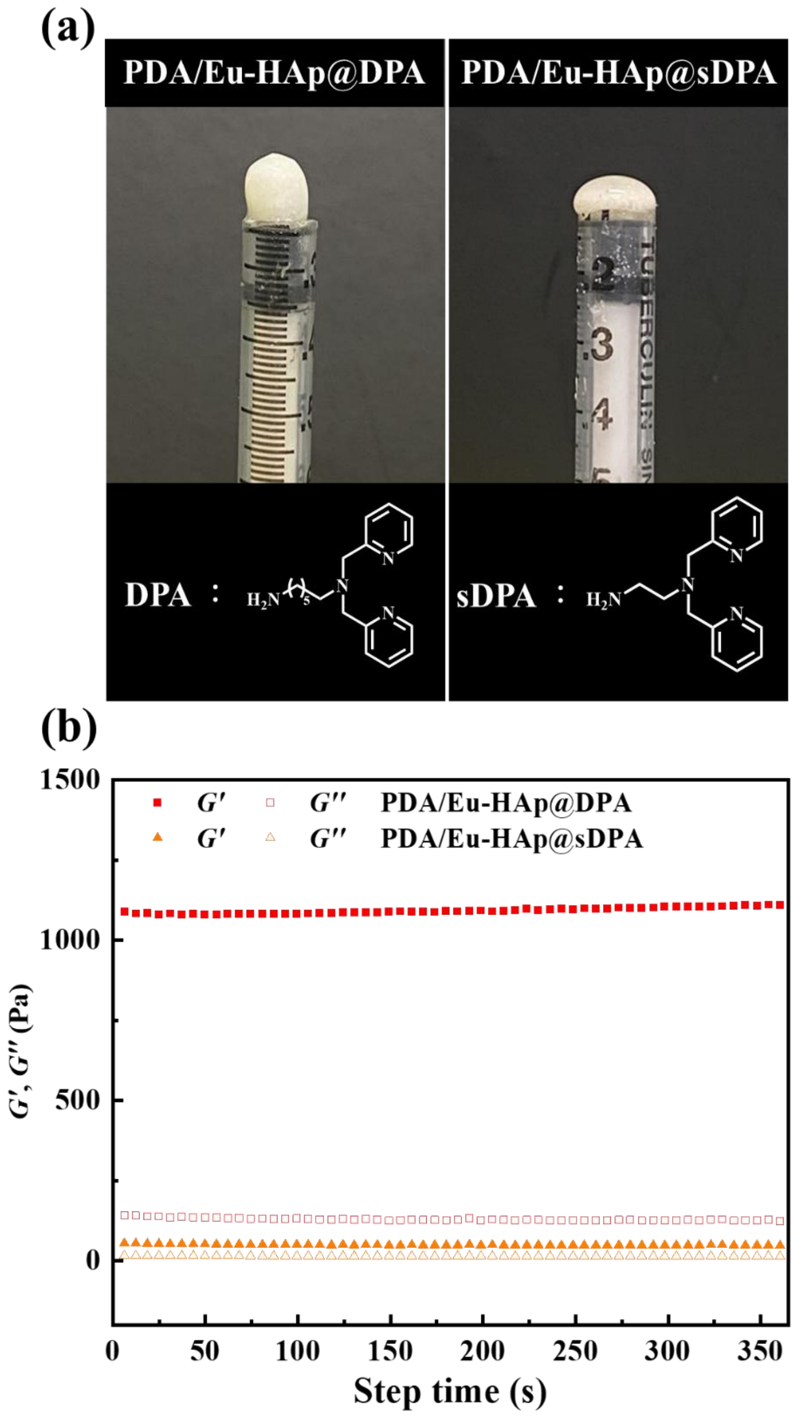

Figure S22. (a) Photos of PDA/Eu-HAp@DPA and PDA/Eu-HAp@sDPA hydrogels. (b) *G'* of PDA/Eu-HAp@DPA and PDA/Eu-HAp@sDPA hydrogels.

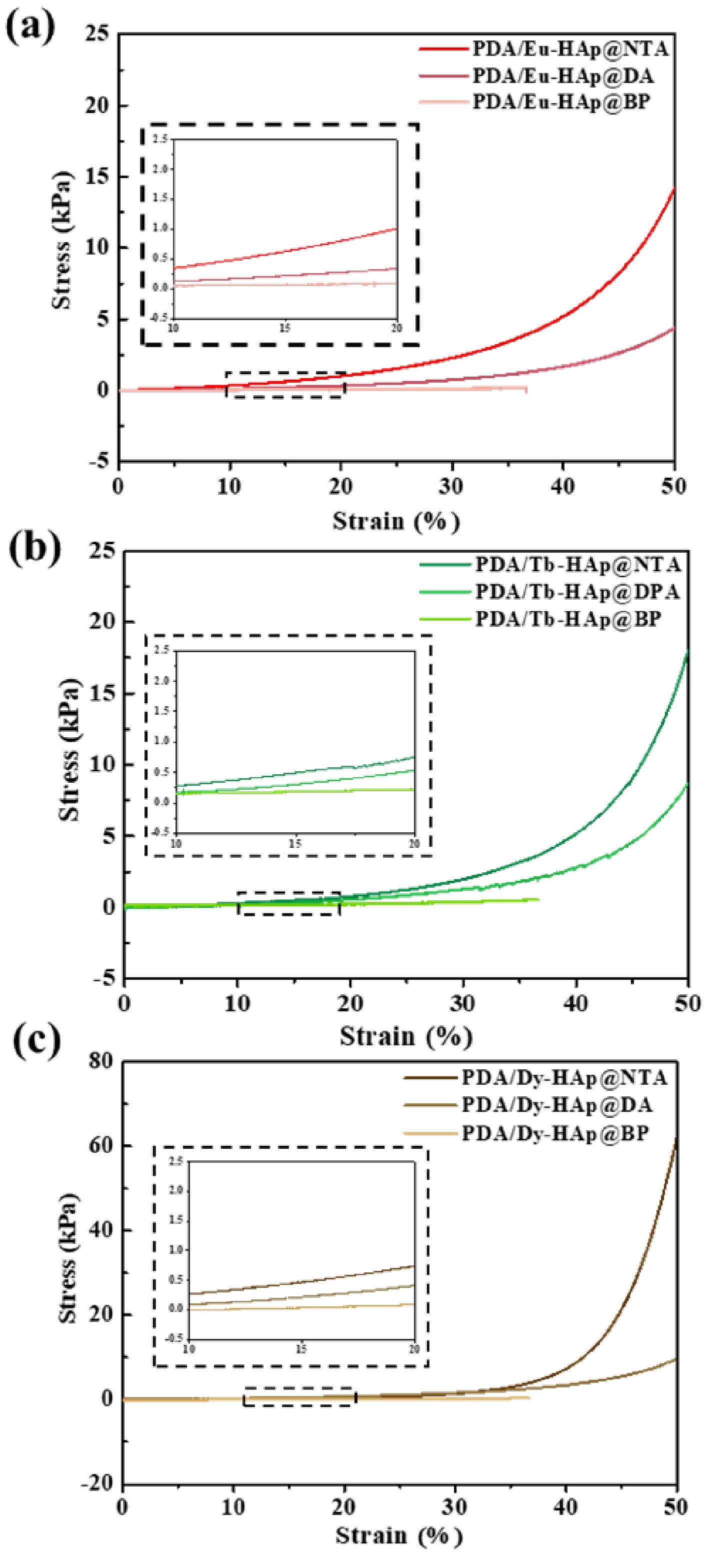

Figure S23. Compressive stress-strain curve of (a) PDA/Eu-HAp@L, (b) PDA/Tb-HAp@L, and (c) PDA/Dy-HAp@L hydrogels

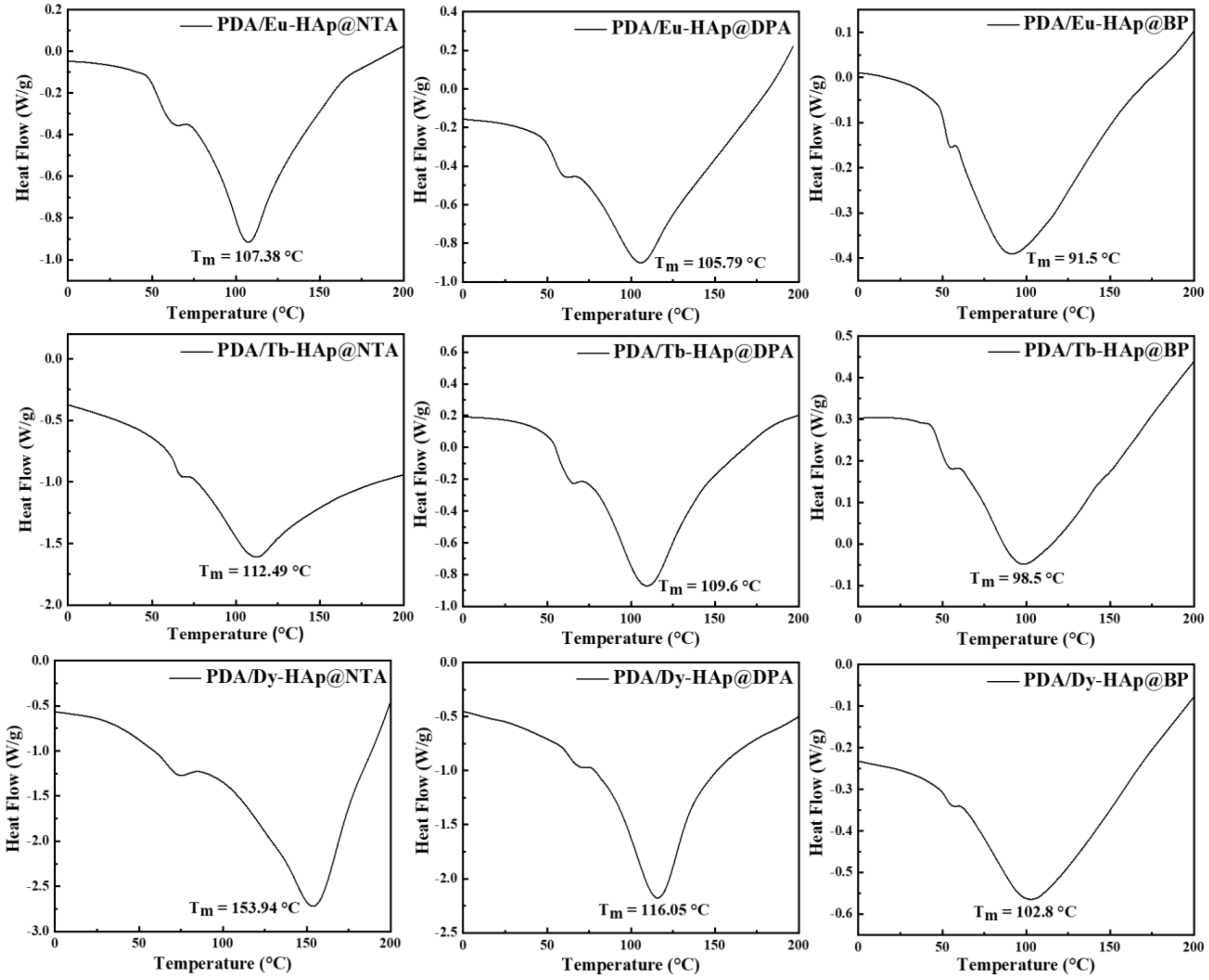

Figure S24. DSC data of PDA/Ln-HAp@L hydrogels.

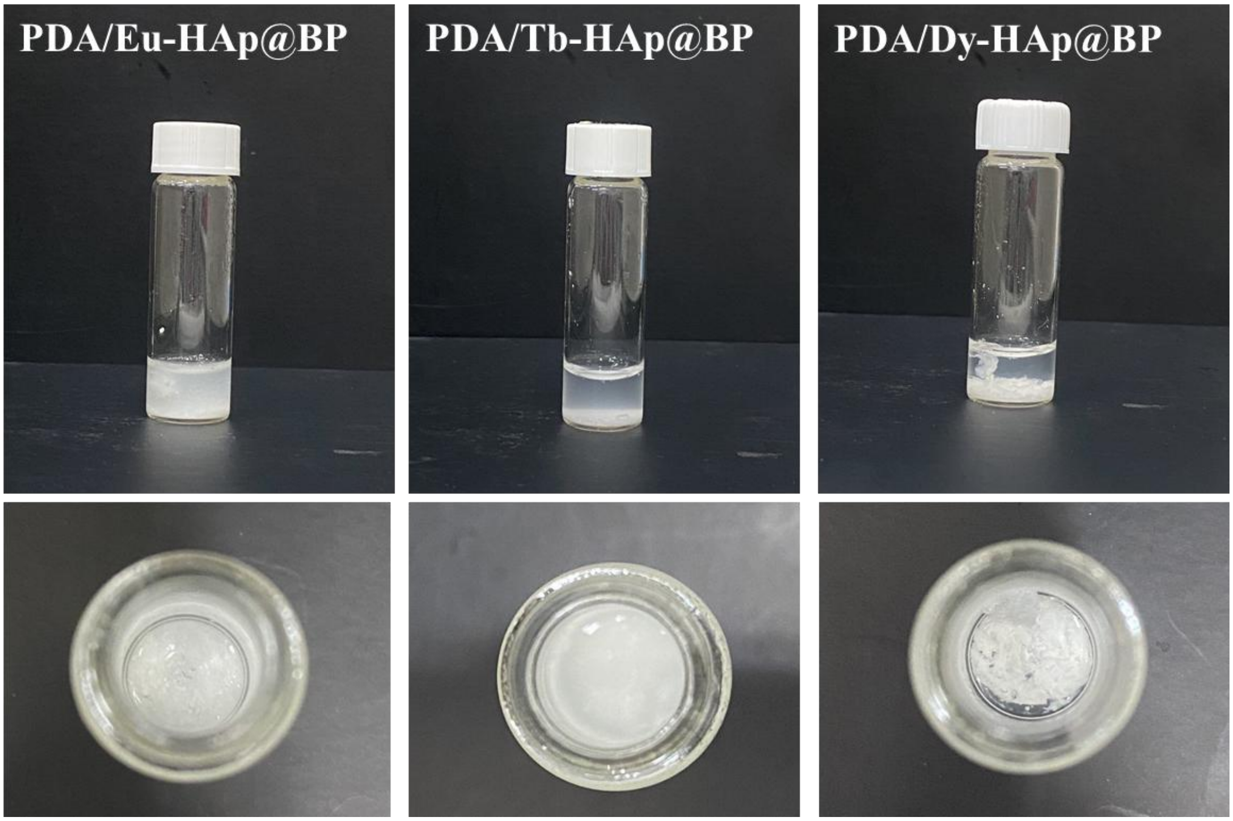

Figure S25. PDA/Ln-HAp@BP hydrogels were dissolved within seconds in water.

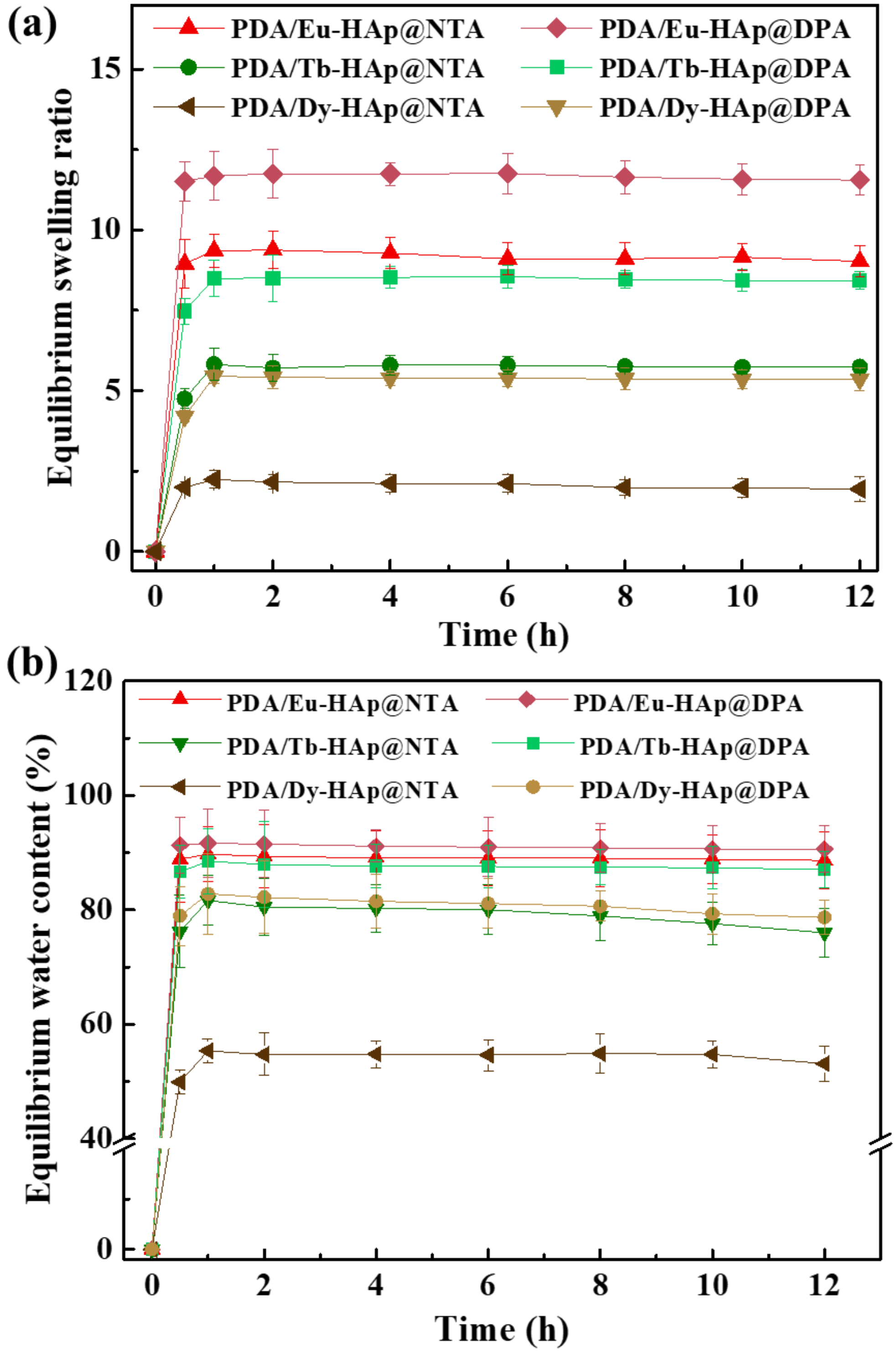

Figure S26. (a) Equilibrium swelling ratio and (b) equilibrium water content of PDA/Ln-HAp@L hydrogels at different time intervals.

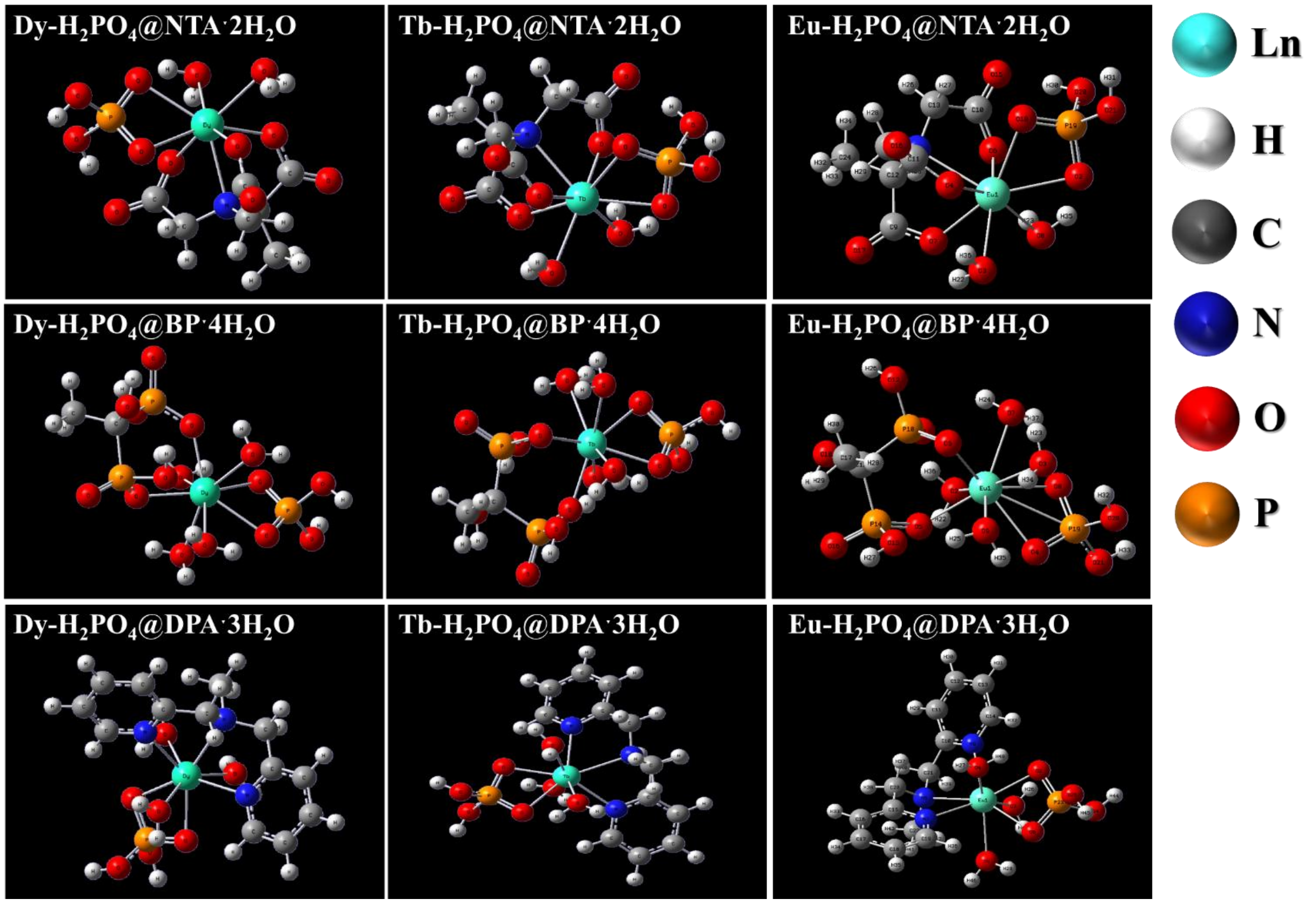

Figure S27. The structure of Ln-H_2PO_4@L·xH_2O.

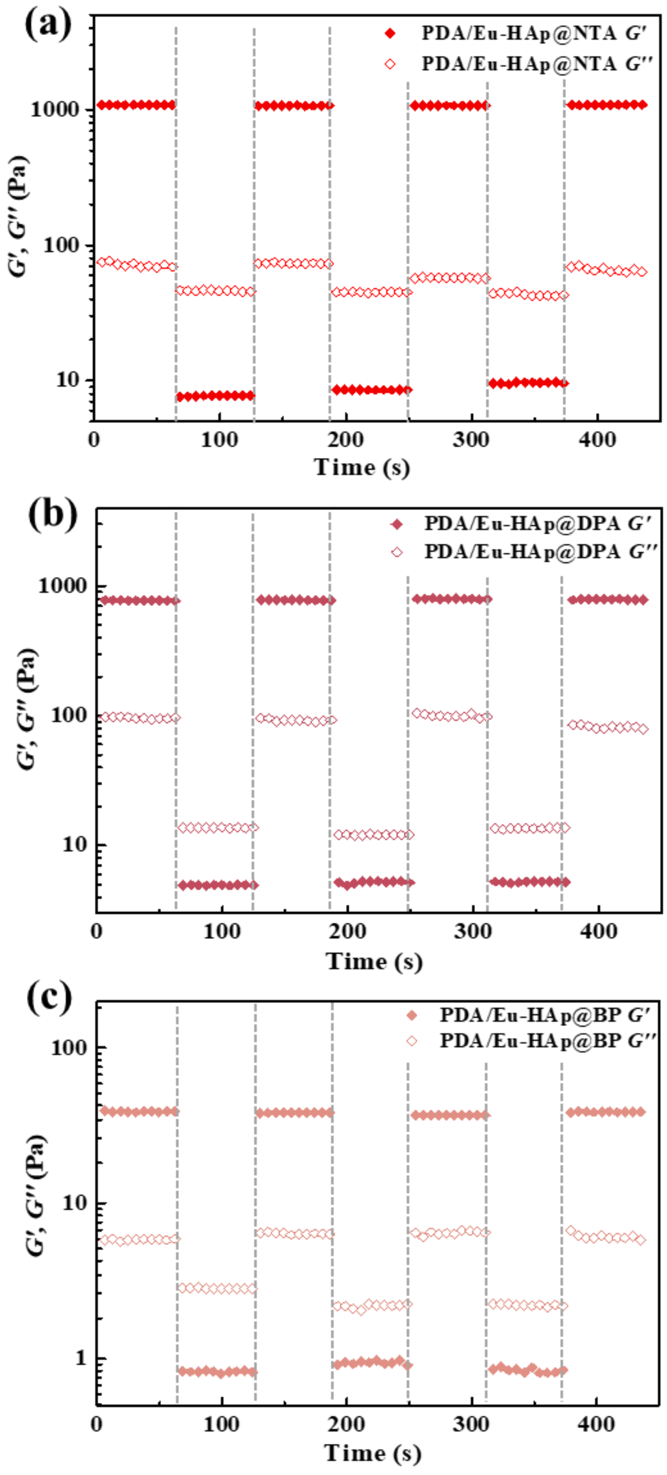

Figure S28. (a-c) *G′* and *G″* of PDA/Eu-HAp@L hydrogels were recorded under the cyclic strain time sweep changes between strains of 1% and 500%.

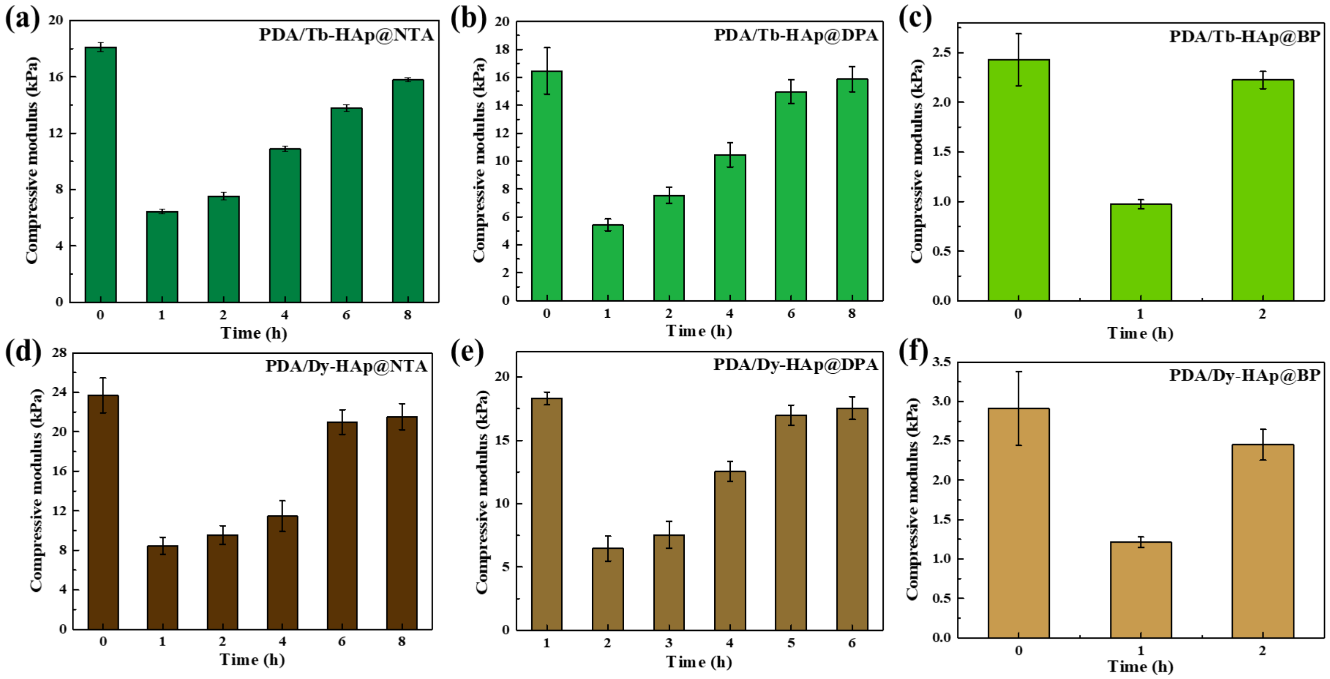

Figure S29. The compressive modulus of (a-c) PDA/Tb-HAp@L and (d-f) PDA/Dy-HAp@L hydrogels before and after healing for different time periods.

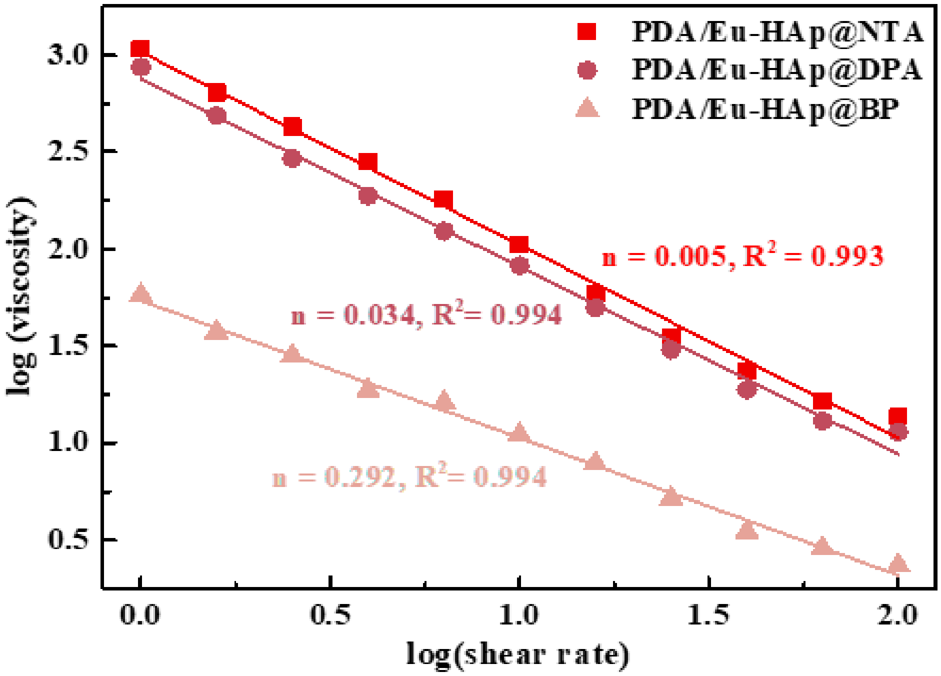

Figure S30. Shear stress-shear rate curves of PDA/Eu-HAp@L hydrogels.

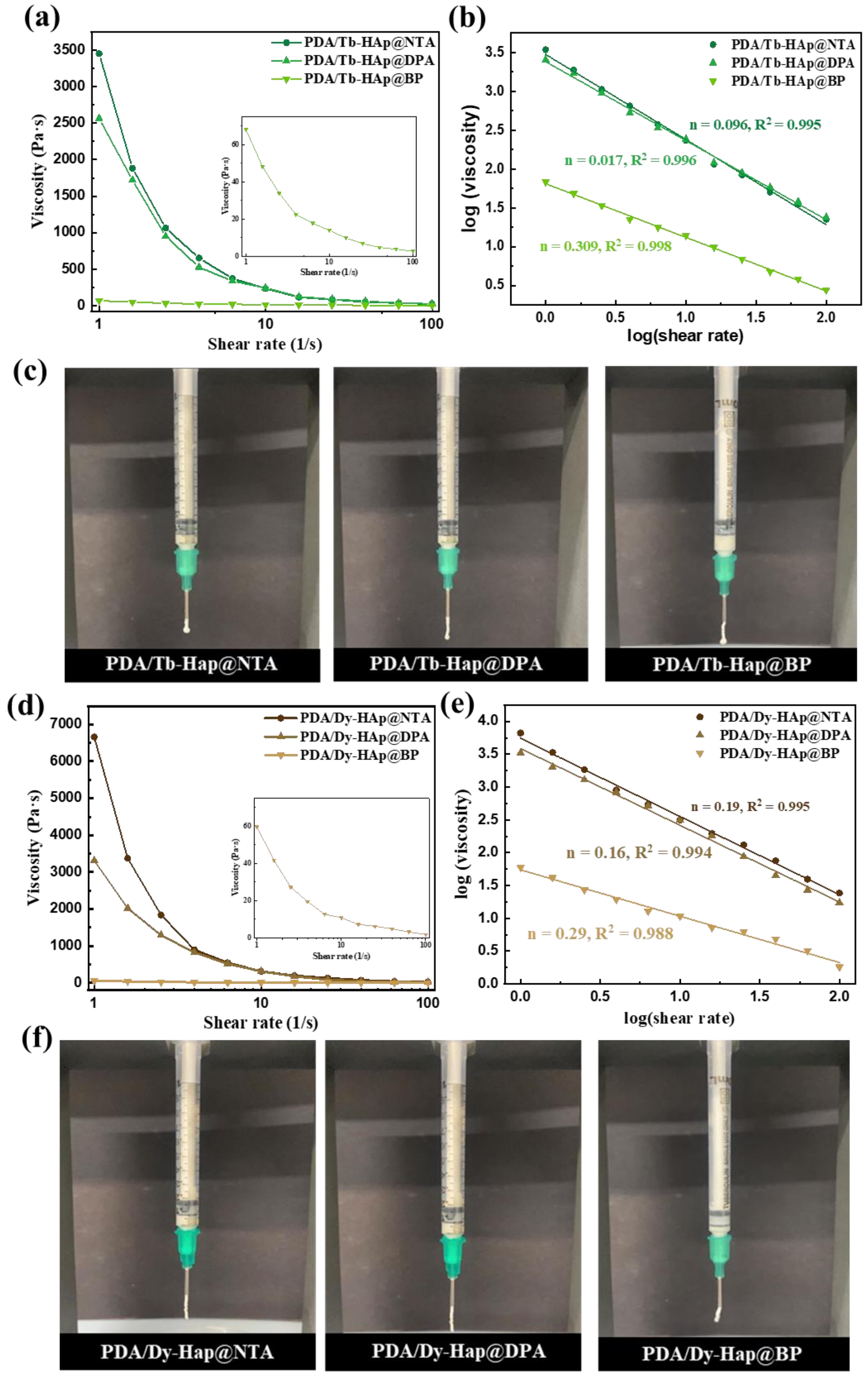

Figure S31. (a) Continuous flow sweeps, (b) shear stress-shear rate plots, and (c) demonstrations of the injectability of PDA/Tb-HAp@L hydrogels. (d) Continuous flow sweeps, (e) shear stress-shear rate curves, and (f) demonstrations of the injectability of PDA/Dy-HAp@L hydrogels.

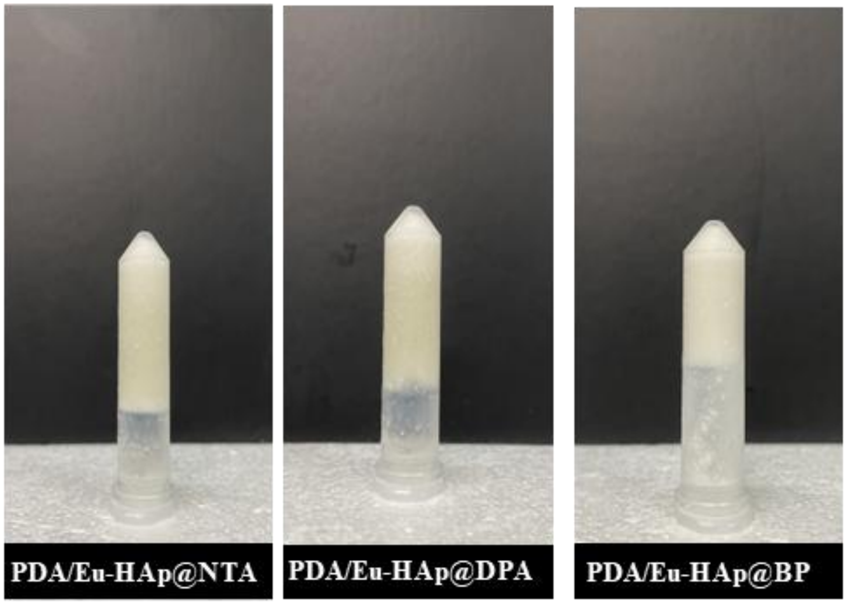

Figure S32. Demonstrations of inversion test of PDA/Eu-HAp@L hydrogel.

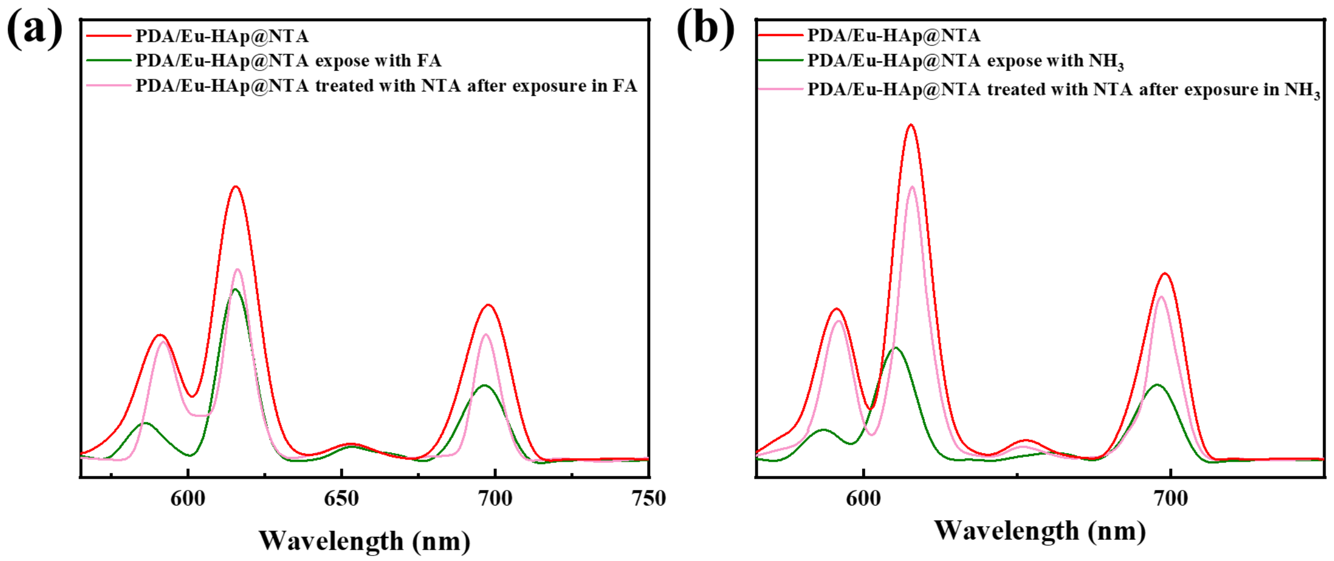

Figure S33. Luminescence spectra of PDA/Eu-HAp@NTA cryogel under UV excitation after exposure to (a) formaldehyde (FA) or (b) ammonia (NH_3), following immersion in the NTA solution (1.0 M) for 2 hrs, and freeze-dried again before measurement.

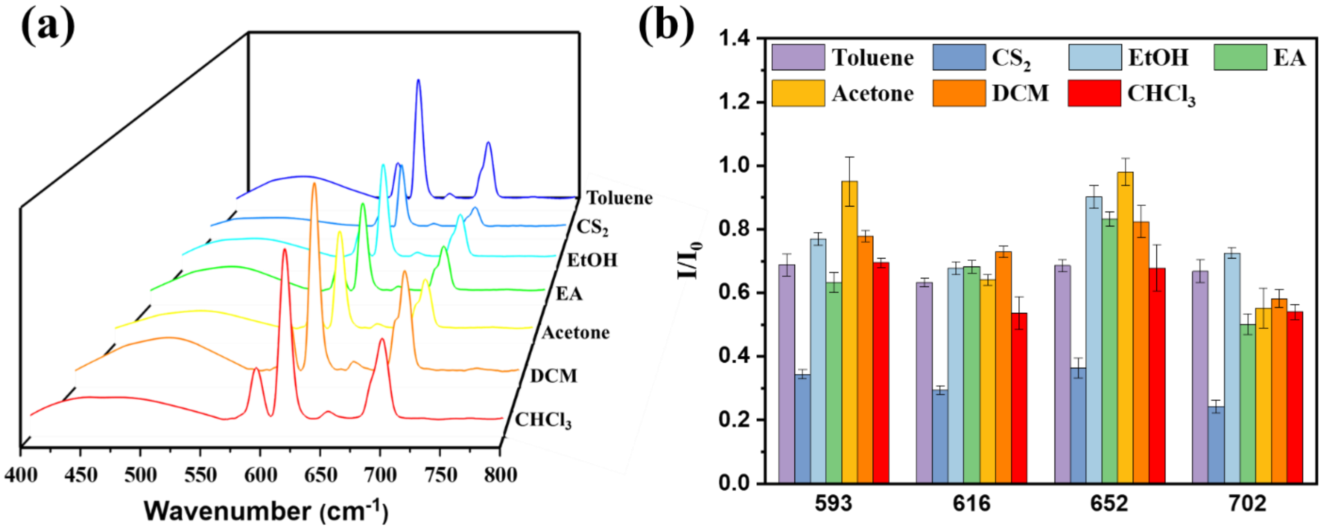

Figure S34. (a) Luminescent spectra and (b) luminescent intensity variety (I/I_0) of PDA/Eu-HAp@NTA lyophilized hydrogel in the presence of different VOCs in the gas phase (λ_{ex}= 360 nm).

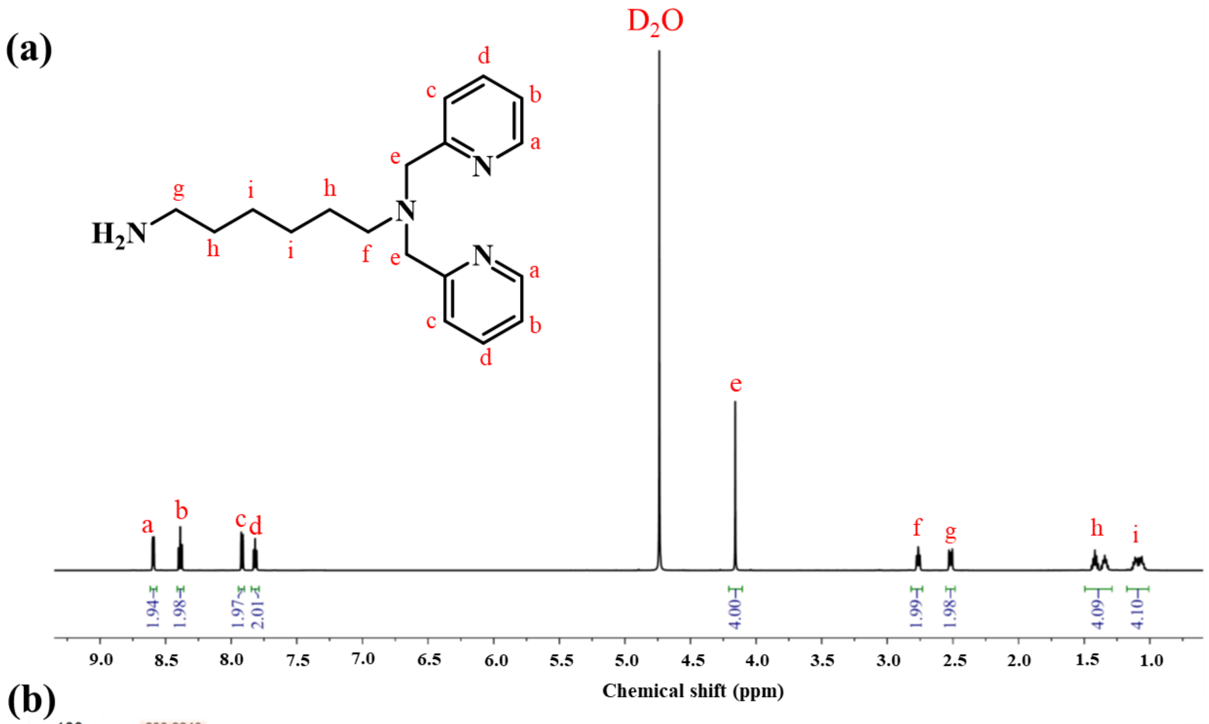

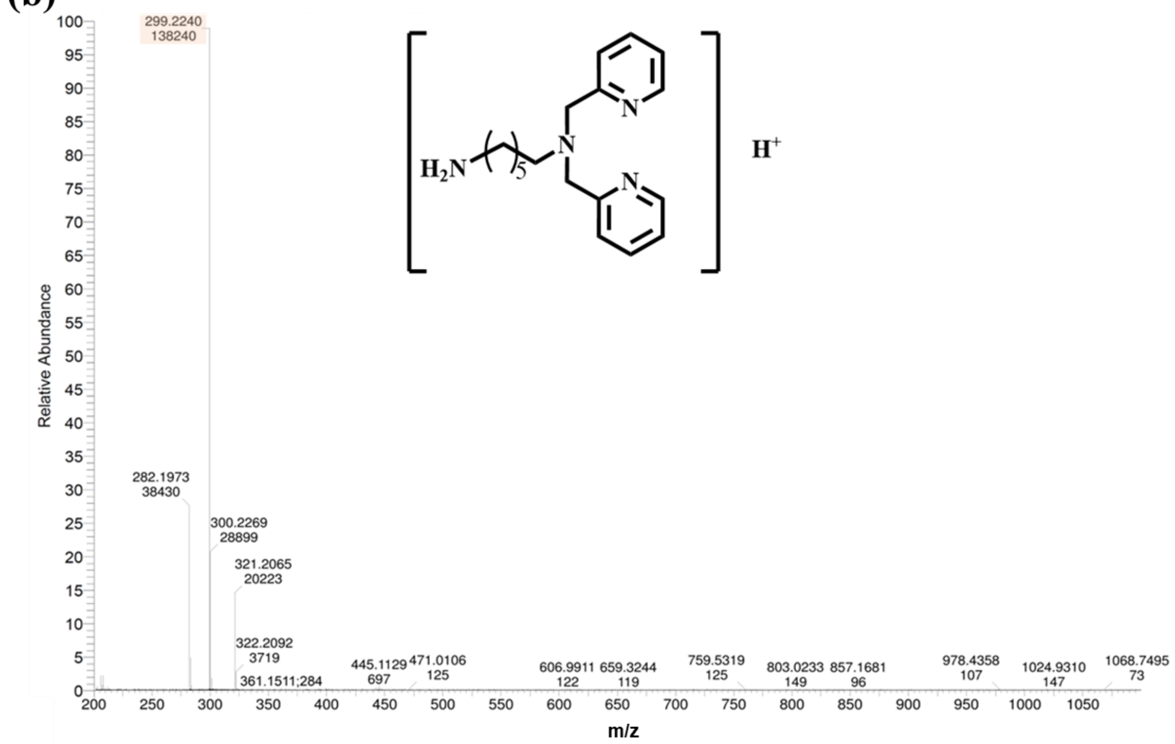

Figure S35. (a) ^{1}H NMR and (b) ESI mass spectra of DPA.

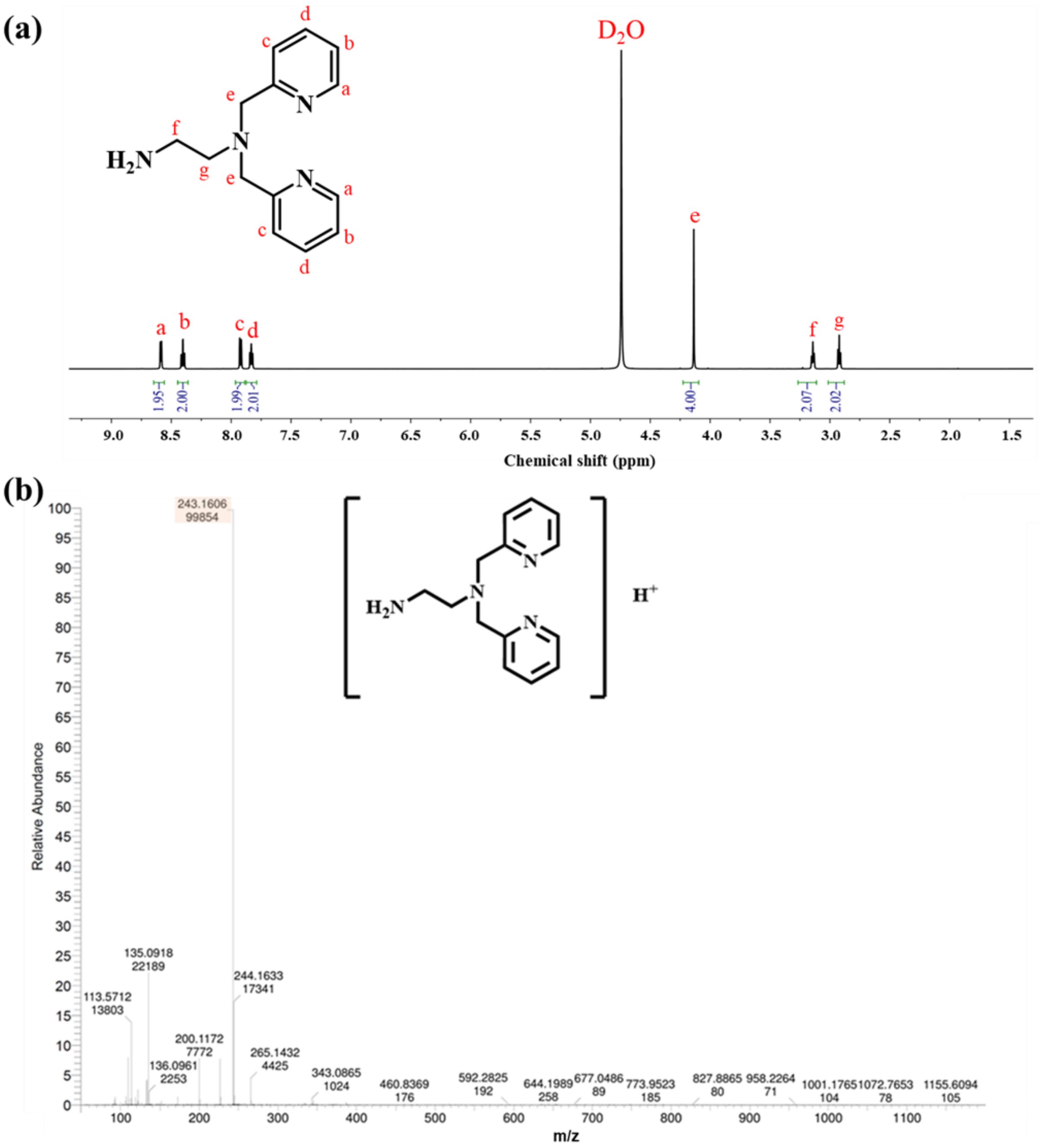

Figure S36. (a) ^{1}H NMR and (b) ESI mass spectra of sDPA.

Table S1. The PLQY values of Ln-HAp@L.

	**PLQY (%)**
Eu-HAp	0.62
Eu-HAp@NTA	1.91
Eu-HAp@DPA	0.96
Eu-HAp@BP	1.59
Tb-HAp	0.14
Tb-HAp@NTA	0.47
Tb-HAp@DPA	0.32
Tb-HAp@BP	0.37
Dy-HAp	0.031
Dy-HAp@NTA	0.62
Dy-HAp@DPA	0.19
Dy-HAp@BP	0.47

Table S2. Ln and amine amount of Ln-HAp and Ln-HAp@L.

	**Ln amount (mmole/g)**	**Amine amount (mmole/g)**
**Eu-HAp**	1.16	-
**Tb-HAp**	1.02	-
**Dy-HAp**	1.12	-
**Eu-HAp@NTA**	0.99	0.90
**Eu-HAp@DPA**	0.95	0.91
**Eu-HAp@BP**	0.95	0.87
**Tb-HAp@NTA**	0.92	0.88
**Tb-HAp@DPA**	0.91	0.87
**Tb-HAp@BP**	0.88	0.85
**Dy-HAp@NTA**	1.01	0.92
**Dy-HAp@DPA**	0.98	0.93
**Dy-HAp@BP**	1.05	0.91

Table S3. The PLQY values of Ln-HAp@L.

	**PLQY (%)** (wet hydrogel/lyophilized hydrogel)	**Reduction percentage of PLQY in the wet hydrogels (%)[a]**
PDA/Eu-HAp@NTA	1.15/1.56	26.3
PDA/Eu-HAp@DPA	0.53/0.69	23.2
PDA/Eu-HAp@BP	0.96/1.24	22.6
PDA/Tb-HAp@NTA	0.25/0.33	24.2
PDA/Tb-HAp@DPA	0.19/0.24	20.8
PDA/Tb-HAp@BP	0.20/0.26	23.1
PDA/Dy-HAp@NTA	0.36/0.46	21.7
PDA/Dy-HAp@DPA	0.11/0.13	15.4
PDA/Dy-HAp@BP	0.26/0.33	21.2

[a.] Reduction percentage of PLQY in the wet hydrogels (%) = $(PLQY_{lyophilized\ hydrogel} - PLQY_{wet\ hydrogel}) / PLQY_{lyophilized\ hydrogel}$

Table S4. MIP analyses of PDA/Ln-HAp@L hydrogels.

	**Total intrusion volume (mL/g)**	**Total pore area (m^2/g)**	**Average pore diameter (nm)**	**Porosity (%)**
**PDA/Eu-HAp@NTA**	2.812	7.357	1530.0	84.55
**PDA/Eu-HAp@DPA**	2.854	8.052	674.7	83.85
**PDA/Eu-HAp@BP**	3.468	8.830	2244.1	78.42
**PDA/Tb-HAp@NTA**	2.643	13.982	756.1	79.53
**PDA/Tb-HAp@DPA**	2.670	12.222	882.7	79.67
**PDA/Tb-HAp@BP**	3.231	7.821	657.7	87.86
**PDA/Dy-HAp@NTA**	2.455	16.915	580.5	76.78
**PDA/Dy-HAp@DPA**	2.640	12.939	816.2	76.89
**PDA/Dy-HAp@BP**	3.039	6.530	1861.3	84.56

Table S5. Micro-CT analyses of PDA/Ln-HAp@L hydrogels.

	**Pore size (μm)**	**Closed porosity (%)**	**Open porosity (%)**	**Total porosity (%)**
**PDA/Eu-HAp@NTA**	46.5	0.0234	66.58	66.60
**PDA/Eu-HAp@DPA**	54.0	0.0597	55.93	55.99
**PDA/Eu-HAp@BP**	54.3	0.706	60.53	61.24
**PDA/Tb-HAp@NTA**	44.3	0.0341	61.18	61.21
**PDA/Tb-HAp@DPA**	44.9	0.0267	78.09	78.12
**PDA/Tb-HAp@BP**	57.3	0.135	56.83	56.97
**PDA/Dy-HAp@NTA**	27.7	0.379	30.10	30.48
**PDA/Dy-HAp@DPA**	40.4	0.0687	68.87	68.94
**PDA/Dy-HAp@BP**	59.9	0.0937	70.82	70.91

Table S6. Rheological analyses of PDA/Ln-HAp@L hydrogels.

	**Storage modulus (Pa)**	**Loss modulus (Pa)**	**Crosslinking density ($mol \cdot m^{-3}$)**	**Flow point (%)**
**PDA/Eu-HAp@NTA**	1265.97 ± 39.49	103.80 ± 3.42	0.51 ± 0.016	41.81 ± 1.25
**PDA/Eu-HAp@DPA**	757.26 ± 46.19	95.15 ± 5.42	0.31 ± 0.019	23.80 ± 1.40
**PDA/Eu-HAp@BP**	39.02 ± 2.84	5.77 ± 0.39	0.02 ± 0.001	11.26 ± 1.01
**PDA/Tb-HAp@NTA**	3464.19 ± 169.74	572.91 ± 29.21	1.40 ± 0.069	68.98 ± 3.67
**PDA/Tb-HAp@DPA**	2335.95 ± 82.22	371.06 ± 12.45	0.94 ± 0.033	33.94 ± 1.07
**PDA/Tb-HAp@BP**	39.20 ± 1.62	5.77 ± 0.02	0.02 ± 0.001	11.17 ± 0.04
**PDA/Dy-HAp@NTA**	8588.26 ± 565.02	1226.95 ± 80.24	3.47 ± 0.228	99.52 ± 6.48
**PDA/Dy-HAp@DPA**	3497.40 ± 298.64	848.75 ± 47.55	1.41 ± 0.121	54.34 ± 3.38
**PDA/Dy-HAp@BP**	69.95 ± 4.08	16.97 ± 1.11	0.03 ± 0.002	22.85 ± 1.28

Table S7. Compression tests of PDA/Ln-HAp@L hydrogels.

	**Compressive modulus (Pa)**
**PDA/Eu-HAp@NTA**	13.57 ± 0.69
**PDA/Eu-HAp@DPA**	8.56 ± 1.25
**PDA/Eu-HAp@BP**	1.77 ± 0.47
**PDA/Tb-HAp@NTA**	18.10 ± 0.32
**PDA/Tb-HAp@DPA**	16.46 ± 1.66
**PDA/Tb-HAp@BP**	2.43 ± 0.26
**PDA/Dy-HAp@NTA**	23.69 ± 1.76
**PDA/Dy-HAp@DPA**	18.31 ± 0.50
**PDA/Dy-HAp@BP**	2.91 ± 0.47

Table S8. Optimized structures (xyz coordinates) from DFT calculations

Eu-H_2PO_4@BP · $4H_2O$

Eu	-0.6816630	-0.2885750	0.1331420	O	-4.9865080	0.3717010	0.6472030
O	-1.0664090	0.8276130	-2.1081300	O	-3.9343030	1.3981730	-1.4546210
O	-1.2134030	-1.9819890	1.9244700	H	-1.9160610	1.2700860	-2.2469390
O	-2.5381820	1.2873070	0.6345890	H	-1.4965890	-2.8105710	1.5200640
O	0.7970100	1.4397690	-0.3630320	H	-0.3939120	-2.7478830	-1.5760130
O	1.2573850	-1.2258070	1.0480920	H	0.5444930	1.2492750	2.3256360
O	-1.0811120	-2.5581260	-0.9235800	H	2.8343250	2.4780470	1.9943680
O	-3.0967070	-0.8663470	-0.5373250	H	4.3215130	-0.0033580	1.5828030
O	-0.3463640	0.8493150	2.3078080	H	5.1053420	1.0369740	0.3676750
P	2.2980800	-1.4186860	-0.0779450	H	5.1321300	-0.7396060	0.1860820
O	1.2660920	-1.2706880	-1.4039520	H	3.7707480	1.1379050	-1.8934500
O	3.1507580	-2.6278840	-0.1282870	H	-5.2033760	1.1652500	1.1520420
C	3.2478280	0.1570870	-0.2939250	H	-4.6326720	1.0015000	-1.9904260
P	2.2631580	1.6918910	0.0366460	H	-0.2944030	-2.1239460	2.2055240
O	2.1892040	1.8324680	1.6812280	H	-0.9891190	1.5684940	2.3427960
O	3.0201060	2.8243710	-0.5768930	H	-0.3913000	1.5143990	-1.9678650
C	4.5348650	0.1139930	0.5159000	H	-1.9380760	-2.4843430	-1.3653790
O	3.5165080	0.2196330	-1.7028370	H	1.7482020	-0.8132190	-2.1153910
P	-3.5895850	0.5067070	-0.1319820				

Tb-H_2PO_4@BP · $4H_2O$

Tb	0.6689280	0.2833380	0.1287760	O	1.0537990	2.5442510	-0.8844370
O	1.0444090	-0.7590610	-2.1200630	O	3.0661030	0.8716660	-0.5185730
O	1.2848990	1.8944970	1.9235890	O	0.3507450	-0.8415550	2.2859530
O	2.4910570	-1.3034680	0.5984860	P	-2.2875300	1.4188620	-0.0655310
O	-0.7869580	-1.4277270	-0.3710580	O	-1.2715640	1.2798360	-1.4032010
O	-1.2324140	1.2164050	1.0455570	O	-3.1407570	2.6283700	-0.0944140

C	-3.2415140	-0.1542910	-0.2837790
P	-2.2519090	-1.6893080	0.0277550
O	-2.1756160	-1.8468160	1.6703650
O	-3.0048110	-2.8180610	-0.5974230
C	-4.5211750	-0.1168620	0.5377400
O	-3.5230270	-0.2075550	-1.6905280
P	3.5532770	-0.5104530	-0.1398430
O	4.9432010	-0.3974500	0.6547330
O	3.9072180	-1.3734600	-1.4787330
H	1.8952340	-1.1950010	-2.2722480
H	1.5450860	2.7297940	1.5167710
H	0.3560810	2.7316010	-1.5270480
H	-0.5365630	-1.2488470	2.3074050
H	-2.8140730	-2.5018640	1.9773810
H	-4.2984720	-0.0072670	1.6035150
H	-5.0934390	-1.0385010	0.3878840
H	-5.1209670	0.7392270	0.2192050
H	-3.7730120	-1.1261140	-1.8857560
H	5.1553640	-1.2038510	1.1408810
H	4.6133280	-0.9688030	-1.9981940
H	0.3930280	2.0382440	2.2775690
H	0.9994610	-1.5563380	2.3047940
H	0.3743380	-1.4530810	-1.9908050
H	1.9045730	2.4736770	-1.3385620
H	-1.7619660	0.8316000	-2.1147150

Dy-H_2PO_4@BP · $4H_2O$

Dy	0.6642780	0.2819850	0.1301600
O	1.0382440	-0.7132520	-2.1255830
O	1.3018340	1.8676690	1.9175180
O	2.4690630	-1.3123650	0.5775560
O	-0.7798080	-1.4177190	-0.3810470
O	-1.2221550	1.2086260	1.0446470
O	1.0391940	2.5272900	-0.8830220
O	3.0566630	0.8779420	-0.4993080
O	0.3554780	-0.8451740	2.2737090
P	-2.2873880	1.4171840	-0.0562050
O	-1.2844060	1.2891040	-1.4029620
O	-3.1437680	2.6247380	-0.0663340
C	-3.2400640	-0.1565290	-0.2772680
P	-2.2430200	-1.6896950	0.0177330
O	-2.1633860	-1.8617020	1.6585760
O	-2.9913020	-2.8162560	-0.6168010
C	-4.5138490	-0.1277240	0.5535620
O	-3.5317300	-0.2020720	-1.6822310
P	3.5384340	-0.5112850	-0.1415440
O	4.9244990	-0.4166080	0.6619070
O	3.8961090	-1.3534190	-1.4926920
H	1.8895910	-1.1456210	-2.2852750
H	1.5727890	2.7010850	1.5139300
H	0.3358380	2.7054740	-1.5225380
H	-0.5305930	-1.2549550	2.2963470
H	-2.7985490	-2.5219590	1.9611900
H	-4.2838960	-0.0241820	1.6183760
H	-5.0850280	-1.0497710	0.4019490
H	-5.1180110	0.7288070	0.2446420
H	-3.7765970	-1.1211330	-1.8818470

H	5.1327590	-1.2324320	1.1338180	H	0.3699810	-1.4110240	-2.0073250
H	4.6076760	-0.9441570	-2.0009790	H	1.8875890	2.4574670	-1.3415220
H	0.4161070	2.0230070	2.2814250	H	-1.7809290	0.8452910	-2.1130030
H	1.0057940	-1.5588830	2.2812940				

Eu-H_2PO_4@DPA · $3H_2O$

Eu	-0.3226750	-0.2591880	0.2327710	O	-3.7401090	-1.8599310	-1.4819610
O	-2.5106600	0.1829990	-0.7002780	H	-1.9083410	1.6113210	2.1393000
O	-1.2587860	0.9019440	2.2253600	H	0.1683270	-0.9314410	-2.9078020
O	-0.3998020	-0.5761410	-2.2154210	H	-0.9152960	-2.3324610	2.1360560
N	1.9025370	0.8343200	1.0638680	H	1.9566900	4.7870430	0.2247200
O	-2.0424460	-1.9182360	0.5104710	H	0.4683080	5.8417570	-1.4806180
N	1.7753320	-1.4996850	-0.4552840	H	-1.4322350	4.4954310	-2.4201660
N	-0.1200220	2.1953180	-0.3384620	H	-1.7391580	2.1508630	-1.6211710
O	-0.1506470	-1.7440080	2.2592630	H	5.0687340	-0.8671920	-0.7502520
C	0.8878300	2.9220610	0.1866340	H	5.0635990	-3.2642570	-1.4475840
C	1.1309350	4.2320890	-0.2093990	H	2.8850130	-4.5123280	-1.4747580
C	0.2976010	4.8187870	-1.1589950	H	0.8122180	-3.2997600	-0.8136170
C	-0.7543420	4.0751990	-1.6852860	H	2.5296090	1.2205220	-0.9006090
C	-0.9256270	2.7680390	-1.2516580	H	3.8587820	0.9974890	0.2379860
C	2.9415430	-0.8250480	-0.4351440	H	1.1385650	2.3837290	2.2251020
C	4.1426020	-1.4322540	-0.7839670	H	2.6481380	2.7979650	1.4121880
C	4.1369140	-2.7704320	-1.1710440	H	2.6298050	-0.8281340	2.1437250
C	2.9310490	-3.4674260	-1.1877280	H	1.6915210	0.3453120	3.1033630
C	1.7752730	-2.7933540	-0.8198680	H	3.3609170	0.7175490	2.6167860
C	2.8618480	0.6260380	-0.0388870	H	-5.1276020	-0.5049930	0.4880660
C	1.6895460	2.2757210	1.2831210	H	-4.1709460	-2.7104350	-1.3180990
C	2.4264990	0.2333670	2.3026980	H	0.4901490	-2.1857360	2.8268520
P	-3.1401700	-1.1212570	-0.2046010	H	-1.5053460	0.3871780	3.0046900
O	-4.3244690	-0.9140630	0.8392050	H	-1.2800190	-0.4186570	-2.5865650

Tb-H_2PO_4@DPA · $3H_2O$

Tb	-0.3394640	-0.1672880	0.2796390	O	-3.7217690	-1.4602860	-1.6035740
O	-2.4895450	0.4483450	-0.5450410	H	-1.6741170	1.8131850	2.2188670
O	-1.0908430	1.0456220	2.2749360	H	0.0744320	-0.8810370	-2.8099750
O	-0.4385940	-0.3872370	-2.1607340	H	-1.0802080	-2.2122660	2.1150260
N	2.0015730	0.6500420	1.0078830	H	2.4740100	4.5630120	0.0929860
O	-2.0753140	-1.7935870	0.4024950	H	1.0219050	5.7680180	-1.5425620
N	1.5226210	-1.6705030	-0.4402000	H	-1.0714470	4.6430630	-2.3542930
N	0.0856240	2.2233030	-0.3170650	H	-1.5964090	2.3528790	-1.5091370
O	-0.3107980	-1.6444610	2.2951690	H	4.8511360	-1.4714690	-0.9029080
C	1.1973580	2.8336420	0.1417380	H	4.5050620	-3.8609460	-1.5377980
C	1.5668990	4.1032920	-0.2866380	H	2.1872220	-4.8191520	-1.4326120
C	0.7536290	4.7742350	-1.1969400	H	0.3176800	-3.3338460	-0.7067070
C	-0.4052690	4.1528790	-1.6527880	H	2.5968040	0.9243490	-0.9854500
C	-0.6994910	2.8772290	-1.1926640	H	3.9306390	0.5530860	0.1089310
C	2.7644980	-1.1502720	-0.4908520	H	1.4938130	2.2983880	2.1735710
C	3.8598040	-1.9129490	-0.8794140	H	2.9954970	2.5142110	1.2749810
C	3.6636510	-3.2466620	-1.2315340	H	2.5646690	-1.0719020	2.0938690
C	2.3793700	-3.7833350	-1.1746760	H	1.8112120	0.2214060	3.0633890
C	1.3374620	-2.9601280	-0.7714520	H	3.4929540	0.3814480	2.5098580
C	2.8852640	0.3062060	-0.1245150	H	-5.1324960	-0.3162410	0.5089770
C	1.9756030	2.1103310	1.2065440	H	-4.1961410	-2.3018950	-1.5529990
C	2.4961430	0.0085950	2.2390490	H	0.3019940	-2.1291800	2.8587820
P	-3.1488810	-0.8958910	-0.2265090	H	-1.3402290	0.5517940	3.0668470
O	-4.3547300	-0.8088230	0.8060530	H	-1.2951490	-0.1696520	-2.5544380

Dy-H_2PO_4@DPA · $3H_2O$

Dy	-0.3491700	-0.0930100	0.3087450	O	-2.4194700	0.7602960	-0.4492770

O	-0.8932780	1.2040340	2.2991070	H	-1.3634260	2.0457820	2.2445450
O	-0.4646590	-0.2529090	-2.1296880	H	-0.0465170	-0.8644850	-2.7460410
N	2.0836130	0.3928420	0.9702500	H	-1.3230430	-2.0541520	2.1076000
O	-2.2058470	-1.5700320	0.3378000	H	3.0578450	4.2029580	0.0040770
N	1.2440570	-1.8476300	-0.4294870	H	1.7232290	5.5924940	-1.5843800
N	0.3602960	2.2095660	-0.3044410	H	-0.5326170	4.7626530	-2.3114710
O	-0.5170680	-1.5508970	2.3173470	H	-1.3309640	2.5639790	-1.4361440
C	1.5604910	2.6634790	0.1102120	H	4.5513860	-2.1315120	-0.9894230
C	2.0832510	3.8704540	-0.3393140	H	3.8474360	-4.4523380	-1.5817300
C	1.3354130	4.6447850	-1.2231710	H	1.4210240	-5.0673030	-1.3983620
C	0.0864200	4.1867120	-1.6323080	H	-0.1960320	-3.3192400	-0.6391430
C	-0.3606540	2.9630510	-1.1548070	H	2.6621910	0.5626170	-1.0394500
C	2.5453360	-1.5107320	-0.5225960	H	3.9569990	0.0148630	0.0281560
C	3.5081880	-2.4258220	-0.9317670	H	1.8549690	2.1032900	2.1347960
C	3.1122010	-3.7208420	-1.2600470	H	3.3377510	2.1010060	1.1812350
C	1.7666090	-4.0673160	-1.1596390	H	2.4201730	-1.3834030	2.0632810
C	0.8650110	-3.1000650	-0.7381200	H	1.8831600	0.0130300	3.0343720
C	2.8818360	-0.0828420	-0.1781790	H	3.5565780	-0.0746380	2.4416480
C	2.2696670	1.8437570	1.1533310	H	-5.1300290	0.1539790	0.5116780
C	2.5106360	-0.3031310	2.1974340	H	-4.3275570	-1.7527390	-1.6844500
P	-3.1910810	-0.5451760	-0.2398980	H	0.0355280	-2.0877380	2.8959200
O	-4.4108240	-0.4381430	0.7725310	H	-1.2047380	0.7499300	3.0928310
O	-3.7710590	-0.9615820	-1.6671870	H	-1.2864380	0.0587420	-2.5337220

Eu-H_2PO_4@NTA · $2H_2O$

Eu	-0.1545560	-0.8076150	0.2386690	O	-0.5111440	1.2588280	1.3408680
O	-2.6619290	-0.9652020	0.6765090	O	1.9579510	-1.0186900	1.2235930
O	0.8186650	-3.1860730	0.1248820	O	-0.9632040	-1.2035160	2.6742280
O	0.8280520	-1.2448260	-1.7945070	C	2.9092490	-0.1447780	1.1439370
N	1.4707410	1.1027820	-0.4332990	C	-0.5117550	2.2534680	0.5316520

C	1.5237550	-0.4060230	-2.4915920	C	3.5644010	2.2297100	0.4510680
C	2.4242660	1.2525360	0.6944210	H	1.8202100	1.6171360	1.5341510
C	0.5737170	2.2562510	-0.5490130	H	1.1063400	3.2181740	-0.5362950
C	2.1402190	0.7615000	-1.6990420	H	0.0359310	2.1881000	-1.4991890
O	-1.3686020	3.1502010	0.5118200	H	2.2346380	1.6297690	-2.3603830
O	1.8034810	-0.5013920	-3.6797020	H	3.1592310	0.4255170	-1.4768440
O	4.0879420	-0.3314790	1.4237860	H	-2.7937060	2.1570930	0.4999210
O	-1.7452670	0.3976230	-1.1949950	H	-4.5761120	0.3756780	-1.7440090
P	-2.9767990	0.1433150	-0.3279390	H	4.2493560	1.8778660	-0.3245200
O	-3.5205540	1.4998030	0.3168470	H	4.1486620	2.3281980	1.3686130
O	-4.2620920	-0.3381720	-1.1776110	H	3.1889320	3.2194740	0.1696600
H	1.5876530	-2.8802700	0.6428660	H	-1.8748450	-1.0875630	2.3366330
H	-0.7315320	-0.3456190	3.0520810	H	1.0895730	-3.0657930	-0.8009890

Tb-H_2PO_4@NTA · $2H_2O$

Tb	0.1510410	-0.7748910	-0.2350280	O	-1.7713430	-0.5339320	3.6766450
O	2.6255270	-0.9586830	-0.6707920	O	-4.0730660	-0.3662450	-1.4141590
O	-0.7010190	-3.1621410	-0.1403270	O	1.7208550	0.4237110	1.1873750
O	-0.7943230	-1.2442370	1.7799360	P	2.9513460	0.1555760	0.3236280
N	-1.4667730	1.1044690	0.4418280	O	3.5033160	1.5036740	-0.3325530
O	0.4962810	1.2585440	-1.3469010	O	4.2349480	-0.3266510	1.1748470
O	-1.9358860	-1.0267380	-1.2010640	H	-1.4847610	-2.8992750	-0.6585480
O	0.9052600	-1.2337620	-2.6374330	H	0.6677630	-0.3919090	-3.0459540
C	-2.8986170	-0.1654090	-1.1262780	C	-3.5871010	2.1973950	-0.4233790
C	0.4978800	2.2611760	-0.5471060	H	-1.8432880	1.6136820	-1.5213220
C	-1.4934480	-0.4195110	2.4897340	H	-1.1248290	3.2248210	0.5156210
C	-2.4336680	1.2384100	-0.6764510	H	-0.0386890	2.2190440	1.4874630
C	-0.5817530	2.2689480	0.5394880	H	-2.1915950	1.6218410	2.3863680
C	-2.1160220	0.7580330	1.7170210	H	-3.1416200	0.4315160	1.5126500
O	1.3513730	3.1610770	-0.5414430	H	2.7797720	2.1619770	-0.5226140

H	4.5582300	0.3914420	1.7305430	H	-3.2248130	3.1916530	-0.1403880
H	-4.2628400	1.8331210	0.3545430	H	1.8257550	-1.1121670	-2.3281790
H	-4.1776900	2.2901110	-1.3374390	H	-0.9808580	-3.0640520	0.7856580

Dy-H_2PO_4@NTA · $2H_2O$

Dy	0.1488560	-0.7596750	-0.2343450	P	2.9383570	0.1607790	0.3228640
O	2.6079560	-0.9560430	-0.6670600	O	3.4953140	1.5046570	-0.3381360
O	-0.6456690	-3.1494080	-0.1504900	O	4.2198960	-0.3222890	1.1763750
O	-0.7787780	-1.2450360	1.7709280	H	-1.4351020	-2.9075470	-0.6696390
N	-1.4640700	1.1055320	0.4464270	H	0.6397120	-0.4069580	-3.0398800
O	0.4892790	1.2580330	-1.3499010	C	-3.5975130	2.1821760	-0.4081100
O	-1.9246650	-1.0289620	-1.1921070	H	-1.8546910	1.6123470	-1.5142190
O	0.8791230	-1.2433100	-2.6214210	H	-1.1328730	3.2280170	0.5056290
C	-2.8934660	-0.1747970	-1.1161820	H	-0.0388970	2.2341010	1.4814200
C	0.4917130	2.2646190	-0.5548330	H	-2.1665500	1.6180160	2.4002560
C	-1.4776980	-0.4268660	2.4881650	H	-3.1319160	0.4365350	1.5321570
C	-2.4379910	1.2320540	-0.6666260	H	2.7736490	2.1633730	-0.5325230
C	-0.5847890	2.2751580	0.5348230	H	4.5462330	0.3971030	1.7285830
C	-2.1025150	0.7567380	1.7265940	H	-4.2688390	1.8114110	0.3705530
O	1.3438070	3.1657300	-0.5562390	H	-4.1911150	2.2729110	-1.3204140
O	-1.7534540	-0.5508830	3.6746280	H	-3.2414450	3.1782540	-0.1235560
O	-4.0664990	-0.3834030	-1.4043340	H	1.8035080	-1.1217380	-2.3248060
O	1.7075230	0.4359980	1.1836320	H	-0.9301570	-3.0628640	0.7752810

Eu-H_2PO_4@$4H_2O$

Eu	-0.7656350	-0.0219360	-0.0309440	O	-0.5381820	2.3669150	0.2159460
O	1.2292320	0.4714010	-1.0687380	O	-0.9667360	-0.6584090	-2.3341750
O	-3.1033690	0.5494910	0.6114240	P	2.1190800	-0.0178120	0.0966140
O	1.1037430	-0.5454090	1.1400740	O	3.0857400	-1.2076490	-0.2584170
O	-1.1421830	-2.0742110	1.1965280	O	2.9516720	1.2307980	0.5865910

H	-3.7203190	1.1432300	0.1583180	H	3.5918150	1.0984000	1.3020110
H	-1.8052180	-2.7696560	1.3066270	H	-1.6164350	-1.0510180	-2.9343750
H	0.2596690	2.7659460	-0.1658200	H	-0.3447210	-2.3516130	1.6775390
H	-0.1535660	-0.5035660	-2.8423770	H	-3.5841380	0.2263940	1.3879790
H	3.8459580	-1.0283870	-0.8316520	H	-0.9835520	3.0560110	0.7281600

Tb-H_2PO_4@$4H_2O$

Tb	0.7379980	-0.0110950	0.0171480	H	3.6749900	1.1109450	-0.1532300
O	-1.2174460	0.4517630	1.0795810	H	1.8019850	-2.7589920	-1.2193030
O	3.0586280	0.5104540	-0.5982360	H	-0.2426380	2.7652220	0.1704840
O	-1.1083500	-0.5293630	-1.1433120	H	0.2282600	-0.4994850	2.8232260
O	1.1365300	-2.0607720	-1.1481120	H	-3.8256790	-1.0663060	0.8501310
O	0.5427970	2.3510700	-0.2208940	H	-3.6148340	1.0968230	-1.2524610
O	1.0246380	-0.6354270	2.2839950	H	1.7051380	-1.0167270	2.8566780
P	-2.1187630	-0.0263190	-0.0827150	H	0.3500120	-2.3544920	-1.6373960
O	-3.0707510	-1.2295660	0.2651310	H	3.5439640	0.1624790	-1.3611220
O	-2.9659780	1.2227920	-0.5436760	H	0.9898470	3.0288930	-0.7467130

Dy-H_2PO_4@$4H_2O$

Dy	0.7253640	-0.0071510	0.0120370	H	3.6495930	1.1022400	-0.1547750
O	-1.2114710	0.4396170	1.0855300	H	1.7991030	-2.7516450	-1.1818120
O	3.0337410	0.4978820	-0.5952850	H	-0.2363060	2.7615640	0.1782370
O	-1.1092950	-0.5189850	-1.1458260	H	0.2625650	-0.5031790	2.8128710
O	1.1335550	-2.0518990	-1.1286730	H	-3.8167100	-1.0847200	0.8538730
O	0.5435740	2.3417900	-0.2183140	H	-3.6232130	1.1008280	-1.2277280
O	1.0506920	-0.6291170	2.2592520	H	1.7453880	-1.0050870	2.8182150
P	-2.1178390	-0.0296180	-0.0769650	H	0.3524110	-2.3517090	-1.6228930
O	-3.0640870	-1.2392060	0.2635010	H	3.5201000	0.1404100	-1.3531560
O	-2.9703320	1.2214730	-0.5216860	H	0.9896410	3.0151210	-0.7507780

Eu-H_2PO_4@3H_2O

Eu	-0.9091780	-0.0527450	0.0150570
O	-1.3383850	1.3835640	1.8876160
O	-1.1225310	-2.4377660	0.1956240
O	-1.5552150	1.0849640	-2.0036310
O	1.0056920	-0.2946140	1.1443290
O	0.9464040	0.3667800	-1.1796780
P	1.9508210	0.0625220	-0.0372250
O	2.8169560	1.3516610	0.1873480
O	2.8643140	-1.1914400	-0.2862600
H	-2.0602360	1.9666410	2.1680660
H	-1.7434610	-3.0925530	-0.1595310
H	-2.3565430	1.3868600	-2.4565030
H	3.5799470	1.3074590	0.7838020
H	3.5499030	-1.1427720	-0.9702050
H	-0.7992740	1.3365230	-2.5610080
H	-0.4478620	-2.9418070	0.6796570
H	-0.6444460	1.4595830	2.5627010

Tb-H_2PO_4@3H_2O

Tb	-0.8722260	0.0512030	-0.0191380
O	-1.3615280	-1.4355860	-1.7958920
O	-1.1585110	2.3909240	-0.2545480
O	-1.5763020	-1.0011830	1.9921970
O	1.0118220	0.2693710	-1.1507740
O	0.9511530	-0.3346570	1.1862910
P	1.9589250	-0.0590790	0.0385550
O	2.8229760	-1.3542200	-0.1541470
O	2.8721980	1.1999450	0.2570130
H	-2.0955970	-2.0280810	-2.0195710
H	-1.7987020	3.0317560	0.0921410
H	-2.3944910	-1.2768850	2.4316360
H	3.5861630	-1.3262930	-0.7514450
H	3.5578720	1.1689420	0.9419830
H	-0.8399220	-1.2473550	2.5776720
H	-0.5042840	2.9063150	-0.7547620
H	-0.6946630	-1.5471890	-2.4931400

Dy-H_2PO_4@3H_2O

Dy	-0.8553660	0.0499220	-0.0223540
O	-1.3674270	-1.4694660	-1.7437780
O	-1.1693750	2.3656130	-0.2948410
O	-1.5899220	-0.9441810	1.9910670
O	1.0151730	0.2512590	-1.1544470
O	0.9510810	-0.3142800	1.1908010
P	1.9618590	-0.0577740	0.0407590
O	2.8252010	-1.3562880	-0.1294370
O	2.8746560	1.2044320	0.2399740
H	-2.1051540	-2.0678290	-1.9381440
H	-1.8164520	3.0033510	0.0448820
H	-2.4166800	-1.2025470	2.4250840
H	3.5889960	-1.3390510	-0.7264190
H	3.5600840	1.1846870	0.9256460

H	-0.8640700	-1.1836910	2.5923710	H	-0.7121620	-1.6011720	-2.4485970
H	-0.5233740	2.8812880	-0.8055370				

Eu-H_2PO_4@$2H_2O$

Eu	0.9349200	0.0097310	-0.0628360	O	-2.7774590	1.2498020	-0.0815020
O	1.6057110	2.3081930	0.1438760	H	1.0548690	2.9678960	0.5986070
O	-0.8610910	0.2109580	1.1707850	H	2.6415110	-2.5513580	0.5229440
O	-0.9225000	-0.2438710	-1.1969180	H	-3.4510900	-1.3685720	0.8184060
O	1.8066710	-2.2236900	0.1499170	H	-3.5205930	1.2730350	-0.7055350
P	-1.8840510	-0.0301770	0.0141960	H	2.3846420	2.7951470	-0.1712630
O	-2.7348210	-1.3334460	0.1643390	H	1.3193850	-3.0201040	-0.1213890

Tb-H_2PO_4@$2H_2O$

Tb	-0.9070090	0.0000100	0.0010700	O	2.7552980	1.2925330	0.1163740
O	-1.6985210	2.2361280	0.0002430	H	-1.2003080	2.9720320	-0.3947590
O	0.8878430	0.2218070	-1.1839980	H	-2.4972720	-2.6377800	-0.3857330
O	0.8890120	-0.2218170	1.1844200	H	3.4852950	-1.3263740	-0.7566140
O	-1.6988090	-2.2360050	-0.0052850	H	3.4870030	1.3257550	0.7534230
P	1.8840760	-0.0000420	-0.0002680	H	-2.4982490	2.6391720	0.3766660
O	2.7552050	-1.2926070	-0.1177430	H	-1.2022230	-2.9731700	0.3894090

Dy-H_2PO_4@$2H_2O$

Dy	-0.8945730	-0.0000600	0.0006290	O	2.7529080	1.2927970	0.1190420
O	-1.6893430	2.2171850	0.0010290	H	-1.1996250	2.9524270	-0.4059450
O	0.8851060	0.2242200	-1.1830210	H	-2.4801440	-2.6201230	-0.3971470
O	0.8858370	-0.2239670	1.1832660	H	3.4836030	-1.3254850	-0.7583280
O	-1.6880410	-2.2177390	-0.0040180	H	3.4847840	1.3256190	0.7559840
P	1.8825670	0.0001990	-0.0001630	H	-2.4828740	2.6196990	0.3911310
O	2.7531160	-1.2922270	-0.1198120	H	-1.1991330	-2.9533130	0.4033250

BP

P	-1.7082720	-0.2120170	0.1653060	C	-0.0031300	0.6107560	0.0113880
P	1.5734600	-0.4197880	0.0781620	C	0.0275980	1.7515330	1.0184440
O	0.0614890	1.1748290	-1.3260320	H	0.9491530	2.3331820	0.9124670
O	-2.7007580	0.9373450	0.0869560	H	-0.8536320	2.3823440	0.8628210
O	-1.7734050	-1.0215780	-1.2971380	H	0.5580190	0.4779100	-1.8075820
O	1.7737770	-1.2713330	1.2934440	H	-0.0119690	1.3390660	2.0327690
O	1.7249900	-0.9160610	-1.3613000	H	2.7554640	1.2119950	-0.6841400
O	-1.7899350	-1.2416730	1.2582790	H	-1.6746830	-0.3284810	-1.9615910
O	2.7229720	0.8243850	0.1975730				

NTA

O	2.8216330	2.2018510	-0.1185520	C	0.6647520	-2.5367410	-0.2265680
O	-3.5144970	1.2388770	0.6422330	H	1.8881680	-0.2278850	-0.3242150
O	0.8118720	2.5651940	-1.0640160	H	-0.9784330	1.8287870	0.1432460
O	-2.9637770	-0.2182100	-0.9872190	H	-1.2364120	0.7189130	1.5057060
O	1.4770650	-2.3318140	-1.1611010	H	-1.3030730	-1.7122840	-0.1977630
N	-0.1355900	-0.0263630	-0.1622560	H	-0.4286500	-1.5339680	1.3180180
C	1.2064640	0.4752250	0.1677280	C	1.5445150	0.4443500	1.6768490
C	-1.2067070	0.7881830	0.3952670	H	2.5858280	0.7573330	1.7980390
C	-0.3295450	-1.4233340	0.2160950	H	0.9084380	1.1365220	2.2459320
C	1.6236820	1.8799780	-0.3994850	H	1.4103640	-0.5629850	2.0922180
C	-2.6863720	0.5585370	-0.0424850	O	0.5179750	-3.6230330	0.4174310

DPA

N	0.000331	-0.649853	-0.094287	C	2.433064	-0.325008	-0.283729
N	3.596869	-0.97883	-0.222809	C	-2.417433	-0.357742	-0.399965
N	-2.50053	0.909403	-0.823372	C	2.318543	1.044928	-0.02865
C	1.227973	-1.13868	-0.689673	C	-3.414701	-0.95534	0.373563
C	-1.177528	-1.115561	-0.803262	C	3.463694	1.758773	0.301661

C	-4.535065	-0.209577	0.728762
C	4.690227	-0.27918	0.089208
C	-3.576408	1.614377	-0.478239
C	4.682113	1.085644	0.362448
C	-4.620578	1.108792	0.295987
H	1.128592	-1.072059	-1.781016
H	1.435562	-2.198243	-0.453154
H	-1.346509	-2.200993	-0.6547
H	-1.016673	-0.9467	-1.873007
H	1.339072	1.509636	-0.089332
H	-3.305776	-1.988188	0.692314
H	3.408511	2.824422	0.507388
H	-5.323823	-0.650063	1.332263
H	5.620334	-0.843497	0.127798
H	-3.611104	2.639264	-0.843929
H	5.603088	1.600612	0.617649
H	-5.472988	1.73342	0.544169
C	-0.059953	-0.949115	1.322469
H	-0.109204	-2.037612	1.521286
H	0.828595	-0.553106	1.821659
H	-0.938055	-0.475793	1.77041

Table S9. ΔG of the Ln-H_2PO_4@L·xH_2O complexes calculated from the computational simulation.

	**ΔG** (kcal/mol)
**Eu-H_2PO_4@NTA · 2H_2O**	-82.99
**Eu-H_2PO_4@DPA · 3H_2O**	-29.11
**Eu-H_2PO_4@BP · 4H_2O**	-46.44
**Tb-H_2PO_4@NTA · 2H_2O**	-85.37
**Tb-H_2PO_4@DPA · 3H_2O**	-29.78
**Tb-H_2PO_4@BP · 4H_2O**	-48.22
**Dy-H_2PO_4@NTA · 2H_2O**	-86.92
**Dy-H_2PO_4@DPA · 3H_2O**	-30.72
**Dy-H_2PO_4@BP · 4H_2O**	-49.20

Table S10. Analysis of Ln-HAp@L

Ln-HAp@L		ΔG (kcal/mol) (by computational simulation using Ln-H_2PO_4@L·xH_2O)	Luminescence peaks (nm)	PLQY	Hydrodynamic size (nm)	Zeta potential (mV)
**Ln**	**L**					
**Eu^{3+} (r= 94.7 Å)**	**NTA**	-88.92	593, 616, 652, and 702	1.91	1185	11.96 ± 1.09
	**DPA**	-29.11	593, 616, 652, and 702	0.96	1038	11.82 ± 0.69
	**BP**	-46.44	593, 616, 652, and 702	1.59	953	10.52 ± 0.69
**Tb^{3+} (r= 92.3 Å)**	**NTA**	-85.37	489, 543, 588, and 620	0.47	1048	12.33 ± 0.75
	**DPA**	-29.78	489, 543, 588, and 620	0.32	1021	12.11 ± 0.65
	**BP**	-48.22	489, 543, 588, and 620	0.37	902	10.23 ± 0.86
**Dy^{3+} (r= 91.2 Å)**	**NTA**	-86.92	485, 572, 664, and 757	0.62	855	10.22 ± 0.82
	**DPA**	-30.72	485, 572, 664, and 757	0.19	890	9.66 ± 0.66
	**BP**	-49.20	485, 572, 664, and 757	0.47	823	8.96 ± 0.63

Table S11. Analysis of PDA/Ln-HAp@L

PDA/Ln-HAp@L			Gelation time (min)	Luminescence peaks (nm)	Pore size (μm) (by micro-CT)	Crosslinking density (mol/m³) (by rheological tests)
	**Ln**	**L**				
**PDA**	**Eu^{3+} (r= 94.7 Å)**	**NTA**	88.3 ± 7.5	593, 616, 652, and 702	46.5	0.51 ± 0.016
		**DPA**	88.5 ± 7.8	593, 616, 652, and 702	54.0	0.31 ± 0.019
		**BP**	99.0 ± 4.5	593, 616, 652, and 702	54.3	0.02 ± 0.001
	**Tb^{3+} (r= 92.3 Å)**	**NTA**	42.7 ± 3.4	489, 543, 588, and 620	44.3	1.40 ± 0.069
		**DPA**	54.4 ± 4.3	489, 543, 588, and 620	44.9	0.94 ± 0.033
		**BP**	100.0 ± 8.8	489, 543, 588, and 620	57.3	0.02 ± 0.001
	**Dy^{3+} (r= 91.2 Å)**	**NTA**	19.1 ± 1.3	485, 572, 664, and 757	27.7	3.47 ± 0.228
		**DPA**	35.2 ± 3.4	485, 572, 664, and 757	40.4	1.41 ± 0.120
		**BP**	114.5 ± 9.1	485, 572, 664, and 757	59.9	0.03 ± 0.002

Table S12. Analysis of PDA/Ln-HAp@L

PDA/Ln-HAp@L			Compressive modulus (kPa) (by compression tests)	T_m (°C) (by DSC)	Remaining weight after degradation tests (%)	Swelling ratio
	**Ln**	**L**				
**PDA**	**Eu^{3+} (r= 94.7 Å)**	**NTA**	13.57 ± 0.69	61.8	37 ± 1.9	9.37 ± 0.58
		**DPA**	8.56 ± 1.25	59.2	24 ± 1.6	11.75 ± 0.76
		**BP**	1.77 ± 0.47	55.0	-	-
	**Tb^{3+} (r= 92.3 Å)**	**NTA**	18.10 ± 0.32	66.1	52 ± 4.7	5.61 ± 0.43
		**DPA**	16.46 ± 1.66	64.8	37 ± 3.2	8.30 ± 0.71
		**BP**	2.43 ± 0.26	55.1	-	-
	**Dy^{3+} (r= 91.2 Å)**	**NTA**	23.69 ± 1.76	73.1	77 ± 4.1	2.16 ± 0.22
		**DPA**	18.31 ± 0.50	68.8	68 ± 4.0	5.12 ± 0.35
		**BP**	2.91 ± 0.47	56.2	-	-
